# Characterization of cancer-related fibroblasts in bladder cancer and construction of CAFs-based bladder cancer classification: insights from single-cell and multi-omics analysis

**DOI:** 10.3389/fimmu.2025.1580986

**Published:** 2025-09-11

**Authors:** Zhaokai Zhou, Yajun Chen, Zhan Wang, Shuai Yang, Zhengrui Li, Run Shi, Ruizhi Wang, Kui Liu, Xiaojuan Tang, Qi Li, Ran Xu

**Affiliations:** ^1^ Department of Urology, The Second Xiangya Hospital of Central South University, Changsha, China; ^2^ College of Traditional Chinese Medicine, Guangzhou University of Chinese Medicine, Guangzhou, China; ^3^ Department of Urology, The First Affiliated Hospital of Zhengzhou University, Zhengzhou, China; ^4^ Department of Pediatric Urology, Guangzhou Women and Children’s Medical Center, Guangzhou Medical University, Guangzhou, China; ^5^ Department of Oral and Maxillofacial-Head and Neck Oncology, Shanghai Ninth People’s Hospital, Shanghai Jiao Tong University School of Medicine, Shanghai, China; ^6^ Department of Oncology, The First Affiliated Hospital of Nanjing Medical University, Nanjing, China; ^7^ Department of Pediatric Surgery, The First Affiliated Hospital of Henan University of Science and Technology, Luoyang, China; ^8^ Department of Plastic and Reconstructive Surgery, The First Affiliated Hospital of Zhengzhou University, Zhengzhou, Henan, China; ^9^ Department of Pediatric Surgery, The First Affiliated Hospital of Zhengzhou University, Zhengzhou, China

**Keywords:** bladder cancer, single-cell RNA-seq, cancer-associated fibroblasts, molecular subtypes, immune microenvironment

## Abstract

**Background:**

Bladder cancer (BLCA) continues to be a significant cause of cancer mortality in the urinary tract, with therapeutic resistance representing a major barrier to improving patient outcomes. Within the tumor microenvironment (TME), cancer-associated fibroblasts (CAFs) are pivotal drivers of BLCA progression, contributing to immune evasion and therapy resistance. This study leverages single-cell analysis to delineate CAF subclusters and explore the immune characteristics of CAFs-based BLCA classification.

**Materials and methods:**

Signal-cell RNA sequencing (scRNA-seq) datasets were used to identify CAF subpopulations in BLCA, and bulk RNA-seq datasets were used to construct CAFs-based BLCA classification. Next, we comprehensively explored the distinct heterogeneity and characteristics for four CAFs-based BLCA subtypes. Moreover, machine learning algorithms were applied to identify novel potential targets for each subtype, and experimentally validate their effects.

**Results:**

This study identified CAFs closely associated with BLCA development based on scRNA-seq datasets. Through further systematic clustering and functional analysis of CAFs, we successfully identified 10 distinct CAF sub-clusters, including PSCA+ Pericyte, ISG15+ Pericyte, ACTA2+ Smooth muscle cell (SMC), ACTG2+ SMC, CCL21+ inflammatory Pericyte, CD74+ apCAF, STMN1+ pCAF, CXCL14+ mCAF, APOD+ iCAF, CFD+ iCAF. The study identified four pCAFs-based BLCA distinct subtypes with different molecular, functional, and immunologic characteristics. C3 exhibited an immune-rich subtype accompanied by poor clinical prognosis, cell death pathway enrichment, higher expression of MHC molecules and co-stimulatory/co-inhibitory molecules. Conversely, C4 subtype has a smaller number of patients and an optimal prognosis, associated with lower levels of cell death pathway enrichment, lower frequency of tumor mutations, and an “immune desert” TME. C1 is mainly enriched in metabolism-related pathways, and C2 is mainly enriched in the activation of genome instability pathways, accompanied by more frequent mutations and higher Atezolizumab response. Furthermore, this study identified potential target genes or prognostic markers for each subtype.

**Conclusion:**

Various heterogeneous CAF subgroups exist in BLCA, which is closely associated with the development of BLCA. This study identified a promising platform for understanding heterogeneity of CAFs-based BLCA subtypes, providing novel insights into the intricate molecular mechanisms of BLCA. Potential target genes for each subtype provide a basis for diagnosis and screening of BLCA patients.

## 1 Introduction

Bladder cancer (BLCA) represents a globally significant health burden with pronounced epidemiological impact, particularly among males (1, 2). BLCA not only affects the quality of life of patients, but also places a heavy financial burden on the healthcare system. The molecular mechanisms underlying the pathogenesis of BLCA have not been fully clarified to date. Emerging studies underscore the critical role of the tumor microenvironment (TME) in BLCA. The TME is mainly composed of cancer-associated fibroblasts (CAFs), immune cells, extracellular matrix, and numerous signaling molecules, interacting with each other during tumorigenesis to promote tumor progression and drug resistance. Of note, CAFs play the multi-faceted roles in driving BLCA progression, facilitating immune evasion, and conferring resistance to therapies (3–6). CAFs constitute a remarkably heterogeneous and functionally diverse cell population within the TME, originating from various sources and characterized by distinct molecular markers and biological activities (3, 7–9). Through single-cell RNA sequencing (scRNA-seq), Xu et al. identified a new CAF subgroup, MMP11+ myofibroblast (mCAF), which gradually accumulates during BLCA progression and is significantly associated with poor prognosis (10). Utilizing scRNA-seq and spatial transcriptomics, Zheng et al. also identify a previously unknown CAF subset in early-stage BLCA, which is associated with lymphovascular invasion, lymph node metastasis, and poor prognosis in early-stage BLCA (11). In short, CAFs play a crucial role in tumor progression, and their heterogeneity influences treatment response and prognosis.

The increasing importance of immunotherapy and targeted therapies has created an urgent need for novel molecular subtyping of BLCA to develop personalized treatment strategies (12, 13). To elucidate the molecular mechanisms of BLCA and guide individualized patient treatment, various gene expression-based classification systems have classified BLCA into molecular and clinical subtypes (13–15). These findings emphasize the critical role of molecular classifications in prognostic prediction for BLCA patients. Clinical decision-making and tailored therapeutics will benefit from these results, providing a valuable reference for further treatment. However, their specific roles, molecular diversity, and CAF-based classifications in BLCA have remained underexplored.

The bulk RNA-seq analysis intrinsically limits the resolution needed to accurately delineate and characterize these functionally distinct CAF subclusters in TME. Recent advances in high-resolution scRNA-seq and transcriptomic analysis could systematically unravel the heterogeneity of CAFs (16). Therefore, this study leverages scRNA-seq data to comprehensively define molecularly distinct CAF subpopulations within BLCA. The novel pCAF-based classification system was subsequently developed and rigorously validated using different datasets of bulk RNA-seq. Finally, machine learning algorithms were applied to identify novel potential targets for each subtype and experimentally validate their effects.

## 2 Materials and methods

### 2.1 Data sources and single-cell RNA-seq processing

The single-cell RNA-seq datasets, including GSE130001, GSE129845, GSE135337, GSE190888, and GSE222315 were downloaded from the Gene Expression Omnibus (GEO) database (https://www.ncbi.nlm.nih.gov/geo/). The R package Seurat (version 4.4.0) is used for single-cell data preprocessing. Potential doublets were predicted and removed using DoubletFinder (17). Low-quality cells (datasets with fewer than 1000 cells, cells with fewer than 500 genes, or more than 20% mitochondrial content) were removed. Normalization was performed using the LogNormalize method with a scale factor set to 1e5. For downstream analysis, the top 2000 highly variable genes were identified using the FindVariableFeatures function. The unwanted sources of variation were regressed out using the ScaleData function. Dimensionality reduction was performed using principal component analysis (PCA) with the first 30 components. The Harmony algorithm was used for batch correction before clustering analysis (18). The FindNeighbors function was used to construct a shared nearest neighbor (SNN) graph based on edge weights between cells. Subsequently, cell clusters were identified using the FindClusters function and visualized using the uniform manifold approximation and projection (UMAP) algorithm (19). In the initial round of annotation, each cluster was annotated based on known markers collected from established literature (11, 20–22), including Epithelial cells (EPCAM, CD24, KRT18, KRT8, KRT19, CLDN4), Endothelial cells (VWF, PECAM1, CDH5, ENG, CLDN5, ACKR1, PLVAP), Stromal cell (COL1A1, COL1A2, COL3A1, MYH11, ACTA2, DCN), T cells (CD2, CD3D, CD3E, TRAC, TRBC1, CD4, CD8A, CD8B, IL7R), NK cells (PRF1, KLRF1, KLRD1, FGFBP2, NKG7, XCL2), B cells (CD19, CD79A, CD79B, MS4A1), Plasma cells (TNFRSF17, MZB1, IGHG1, IGHA1), Mast cells (TPSAB1, TPSB2, MS4A2), and Myeloid cells (CD14, CD68, CD163, LYZ, S100A8, FCGR3A).

### 2.2 Cell type distribution

To explore the heterogeneity of the TME in each sample, this study demonstrated the composition of the broad cell types in each sample. Next, we further investigated the association between the proportion of cell types and tumor grade to explore potential clinical associations. In addition, we further compared the cell proportion profiles in non-muscle invasive bladder cancer (NMIBC) samples and normal samples. The cell proportion profile further confirmed the importance of CAFs (containing mural cells) in tumorigenesis and development. Subsequently, we clustered all the fibroblasts (containing mural cells) into sub-clusters. Likewise, the top 2000 highly variable genes were identified using the FindVariableFeatures function. The unwanted sources of variation were regressed out using the ScaleData function. Dimensionality reduction was performed using PCA with the first 30 components. The Harmony algorithm was used for batch correction before clustering analysis (18). The FindNeighbors function was used to construct an SNN graph based on edge weights between cells. Subsequently, cell clusters were identified using the FindClusters function and visualized using the UMAP algorithm (19). Based on unsupervised clustering analysis, we chose the medium clustering resolution parameter (RNA_snn_res.0.2) for cellular clustering of fibroblasts (containing mural cells). In the second round of annotation, each cluster was annotated based on known markers collected from established literature (11, 22, 23), including Mural cell (CALD1, NDUFA4L2, COX4I2, RGS5, NOTCH3, DES, MYH11), inflammatory CAF (iCAF) (CCL2, COL14A1, CXCL14, CXCL1, CXCL12, CXCL2, CXCL8, CCL13, IL6, IL11, IL8, CCL5, CCL22, CLEC3B, COL14A1, LY6C, CCL17, CXCL1, CXCL2, CXCL12, CXCL14, LIF, HGF, APOD, IGF1, C3, C7, ITM2A, MGP, CCL11), mCAF (AOC3, COL12A1, COL15A1, COL1A2, COL1A1, COL8A1, FAP, MYL9, TAGLN, MYLK, TPM1, TPM2, POSTN, HOPX, PDGFA, COL5A1, VIM, COL6A1, αSMA, TGFβ, TAGN, CTHRC1,THY1, CTGF, CTA2, FAP, COL12A1, FBLN1, SERPINF1, VCAN, COL11A1, COL10A1, ACTC2), vascular CAF (vCAF) (VEGFA, FGF, PDGF, PDGFα, PDFGRβ, ANG2, CLIC3, ACTA2, ANG1, MMP1, MMP2, MMP3, MMP9, MMP11, MMP14, PDPN, IMF1), antigen-presenting CAF (apCAF) (CD74, HLA-DRA, HLA-DRA1, HLA-DPA1, HLA-DQA1, CD52, IGFBP3, SLP1, SAA3, FSP1), proliferating CAF (pCAF) (NUSAP1, TOP2A, CENPF, PTTG1, SCG2, STMN1, PLAU, TUBA1B, IFI27, IGFBP2). To identify the subpopulations most relevant to tumor grading, we again explored the proportional profile of the CAF sub-clusters.

### 2.3 Enrichment analysis and deconvolution algorithm

Gene Ontology (GO) and Kyoto Encyclopedia of Genes and Genomes (KEGG) were used to analyze the biological process and pathway enrichment of each CAF sub-clusters through the “clusterProfiler” R package. Gene set variation analysis (GSVA) was performed on hallmark pathways obtained in the Molecular Signatures Database (MSigDB, version 7.0).

Bulk RNA-seq data and clinical information about BLCA were sourced from the TCGA database (https://xenabrowser.net/datapages/). The deconvolution algorithm serves as a tool to infer TME composition (different cell populations) and gene expression in bulk RNA data based on scRNA-seq data. In this study, we analyzed the relationship between CAF sub-clusters and survival by deconvolution algorithms such as Multi-subject single cell deconvolution (MuSiC), Convolutional pose machine (CPM), Dampened weighted least squares (DWLS), Bayesprism, and InstaPrism. Survival analysis was performed for each CAF sub-cluster using the optimal cutoff value.

### 2.4 Construction and verification of STMN1+ pCAF-based subtypes

This study performed unsupervised consensus clustering (k-means algorithm, 1,000 iterations) using 1,215 marker genes in the STMN1+ pCAF (adjusted p-value < 0.05, mean log_2_ fold-change > 1) via the “FindMarkers” function. Subsequently, we applied the Nearest Template Prediction (NTP) algorithm to classify TCGA-BLCA tumors into four subtypes. NTP computes subtype probabilities by projecting bulk transcriptomes onto predefined gene signatures through a cosine similarity metric, inherently mitigating technical batch effects. The reproducibility of this subtyping was rigorously validated in four independent cohorts (GSE13507, GSE48075, E−MTAB−4321, and IMvigor210 cohort), and a Meta-data cohort (including GSE13507, GSE19423, and GSE37815). IMvigor210 cohort that was additionally downloaded as external datasets using the “IMvigor210CoreBiologies” R package and the GEO cohorts were used to confirm the stability of STMN1+ pCAF-derived subtypes.

### 2.5 Association of four subtypes with clinicopathological characteristics

Four distinct molecular subtypes (C1, C2, C3, C4) were delineated through consensus clustering. To characterize their clinical relevance, we evaluated each subtype association with clinicopathological parameters: age, sex, TNM stage, histological grade, and growth pattern.

### 2.6 Cell death-related modalities and functional enrichment analysis for four subtypes

To investigate the cell death patterns of each subtype, we downloaded 56 pathways of seven cell death types including apoptosis (n = 12), autophagy (n = 19), necrosis (n = 10), lysosome-dependent cell death (n = 11), pyroptosis (n = 2), necroptosis (n = 1), and ferroptosis (n = 1) from MSigDB (https://www.gsea-msigdb.org/gsea/msigdb/human/search.jsp) and then assessed their enrichment in the four subtypes (24). What’s more, we used the online tool Metascape to construct a network of statistically significant enrichment profiles in the four subtypes to explore the functional differences and the associated pathways. The input genes were screened specifically for each subgroup and have been pre-screened by the COSG algorithm. Meanwhile, KEGG pathway enrichment analyses were performed for the four pCAF-based BLCA subtypes.

### 2.7 Immune microenvironment and genomic alteration analysis

This study quantified the relative abundance of 22 immune cell types across samples from the TCGA-BLCA cohort using CIBERSORT analysis, an algorithm widely used to assess immune cell infiltration. Moreover, unique scores for gene sets in specific samples were calculated by single-sample gene set enrichment analysis (ssGSEA), a method commonly used to assess immune cell infiltration abundance. Based on the R language package “GSVA”, we used ssGSEA to obtain the infiltration status of 28 immune cells within each sample from the TCGA-BLCA data, which enabled the characterization of immune cell properties among different subtypes. Meanwhile, we analyzed the expression levels of 27 immune checkpoints in the BLCA cohort and compared the differential expression among the four groups. To assess the predictive power of cancer immunotherapy risk scores, we included inflammatory genes associated with immune checkpoint blockade (ICB) response. Differences in mutation patterns among the four groups were examined using the “maftools” software package. Additionally, tumor neoantigen load, an indicator of genomic instability, was evaluated.

### 2.10 Machine learning-driven identification of hub genes in four subtypes​

To further identify pivotal genes associated with four subtypes, we used two machine learning algorithms, namely Random Forest (RF) and Least Absolute Shrinkage and Selection Operator (LASSO) regression analyses. The RF models were analyzed using the ‘randomForest’ R package (version 4.7-1.1), and genes with MeanDecreaseGini values greater than 0.6 were selected as candidate genes. LASSO regression using the “glmnet” R package (version 4.1-8). The genes cross-identified by two algorithms were considered as potential hub genes. Thereafter, the hub genes were analyzed for single-gene GSEA enrichment using the “GSEABase” R package (version 1.60.0). The relationship between the three hub genes and 22 immune cells was analyzed using the “corrplot” R package (version 0.92).

### 2.11 Survival analysis of hub genes

The prognostic role of hub genes was evaluated in the TCGA-BLCA cohort and patients were categorized into high and low expression groups using the median expression threshold for each gene to plot the Kaplan-Meier survival curve. Furthermore, differences in stage and grade between the two groups were also compared. Staging and grading differences between the two groups were also compared.

### 2.12 Protein extraction and western blot analysis

Protein extraction and Western blot analysis refer to the steps of our previous studies (14). Among them, primary antibodies included: FAT4 (1:1000; PA5-72970, Invitrogen, China), RPL37P1 (1:1000; ab228542, Abcam, China), FGFR1 (1:1000; R381166, Zenbio, China), RNASEH1 (1:1000; 82771-1-RR, Proteintech, China), AHNAK2 (1:1000; 680021, Zenbio, China), SLC9B2 (1:1000; 24065-1-AP, Proteintech, China), MN1 (1:1000; 24697-1-AP, Proteintech, China), TTLL3 (1:1000; PA5-70598, Invitrogen, China), FABP6 (1:1000; 126828, Zenbio, China), TBC1D3 (1:1000; DF3346, Affinity, China).

### 2.14 Statistical analysis

All data processing was done using R 4.2.2 software. The “survival” R package was utilized to assess disease-specific survival (DSS), progression-free survival (PFS), and overall survival (OS) among subtypes using the Kaplan-Meier method and log-rank test. Specifically, statistical analyses were performed as follows: 1) Continuous variables: Kruskal-Wallis test for multi-group comparisons; Wilcoxon rank-sum or Student’s t-test for pairwise comparisons; 2) Categorical variables: Fisher’s exact or Chi-square test; 3) Pathway enrichment: Mann-Whitney-Wilcoxon Gene Set Test. Statistical significance was defined as two-sided P< 0.05.

## 3 Results

### 3.1 Single-cell atlas reveals CAFs heterogeneity in BLCA

**Figure 1:**
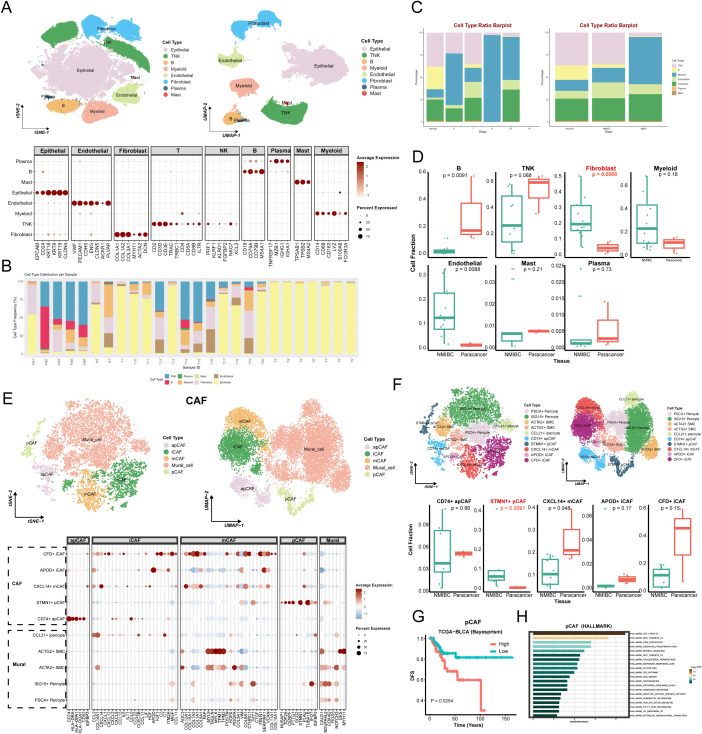
Cellular atlas and identification of CAFs in BLCA. **(A)** The tSNE (left) and UMAP (right) graphic in BLCA and paracancer tissues, color-coded by cell types. Bubble diagram for expression of marker genes in each cell type (bottom). **(B)** The distribution plots of cell types in every samples. **(C)** The distribution plots of cell types in different tumor stages (left) and tissues (right). **(D)** The relative proportions of 7 cell types in NMIBC and paracancer tissues. **(E)** The CAFs’ tSNE (left) and UMAP (right) graphic, color-coded by cell types. Bubble diagram for expression of marker genes in each cell type (bottom). **(F)** The tSNE (left) and UMAP (right) graphic in BLCA and paracancer tissues, color-coded by CAFs sub-cluster. The relative proportions of 5 CAFs in NMIBC and paracancer tissues. **(G)** The survival curve based on pCAF in TCGA BLCA cohort (Bayesprism). **(H)** Pathway enrichment analysis based on pCAF marker genes (HAllMARK). Adj, adjacent; N, normal; RT, recurrent tumor; T, tumor.

In this study, T-SNE and UMAP dimensionality reduction resolved seven major cell clusters within BLCA. The cell types in the clusters were identified by characterized genes and cell type markers in the established literature (11, 20–22), including epithelial cells, fibroblasts, endothelial cells, myeloid cells, TNK cells, plasma cells, and mural cells. **Figure 1A** presents the expression of significant markers in all cell types. Analysis of cell type distribution revealed pronounced inter-patient heterogeneity (**Figure 1B**), corroborating significant heterogeneity of TME among individuals. Of note, the proportion of the seven cell subgroups varied among staging (**Figure 1C**), implicating that changes in cell type may drive tumor progression. Since there were only two cases of muscle-invasive bladder cancer (MIBC) samples, in order to reduce the error caused by too small amount of samples, we compared the cellular composition between NMIBC and paracancerous tissues. Numerous studies have pointed out that CAFs significantly impact tumor progression (20). Likewise, in our study, the proportion of CAFs was significantly higher in tumor tissues compared with paracancerous tissues (**Figure 1D**), further demonstrating that CAFs were intimately associated with tumor progression. The percentage of endothelial cells was also higher in the tumor tissues compared to the paracancerous tissues. Interestingly, T/NK and B cells were higher in the paracancerous tissues, which may suggest that the tumor margin region is the primary place for adaptive immune responses.

Unsupervised clustering of CAFs identified five general clusters: comprising apCAF, iCAF, mCAF, pCAF, and Mural cells (**Figure 1E**). Specifically, this study annotated each of the sub-clusters based on their characteristic genes and functional enrichment, including PSCA+ Pericyte, ISG15+ Pericyte, ACTA2+ Smooth muscle cell (SMC), ACTG2+ SMC, CCL21+ inflammatory Pericyte (iPericyte), CD74+ apCAF, STMN1+ pCAF, CXCL14+ mCAF, APOD+ iCAF, CFD+ iCAF (**Figure 1F**). Heatmap showed each cluster-specific gene expression pattern. Among sub-clusters, STMN1+ pCAFs were significantly enriched in NMIBC samples compared to paracancerous tissues (**Figure 1F** and **Supplementary Figure S1A**). Deconvolution analysis showed that patients with high STMN1+ pCAFs abundance exhibited significantly reduced overall survival (**Figure 1G** and **Supplementary Figure S1B**), suggesting a strong association between STMN1+ pCAFs and aggressive progression of BLCA. GSEA analysis revealed the enrichment of Hallmark_e2f_targets, Hallmark_g2m_checkpoint, Cytochrome-c oxidase activity, Cytochrome complex, Glutathione metabolism, Oxidative phosphorylation, P53 signaling pathway (**Figure 1H** and **Supplementary Figures S1C, D**). These pathways collectively explain pCAF-driven proliferative characteristics, which may potentially contribute to BLCA progression.

### 3.2 Identification and validation of STMN1+ pCAFs-based BLCA heterogeneity subtypes

**Figure 2:**
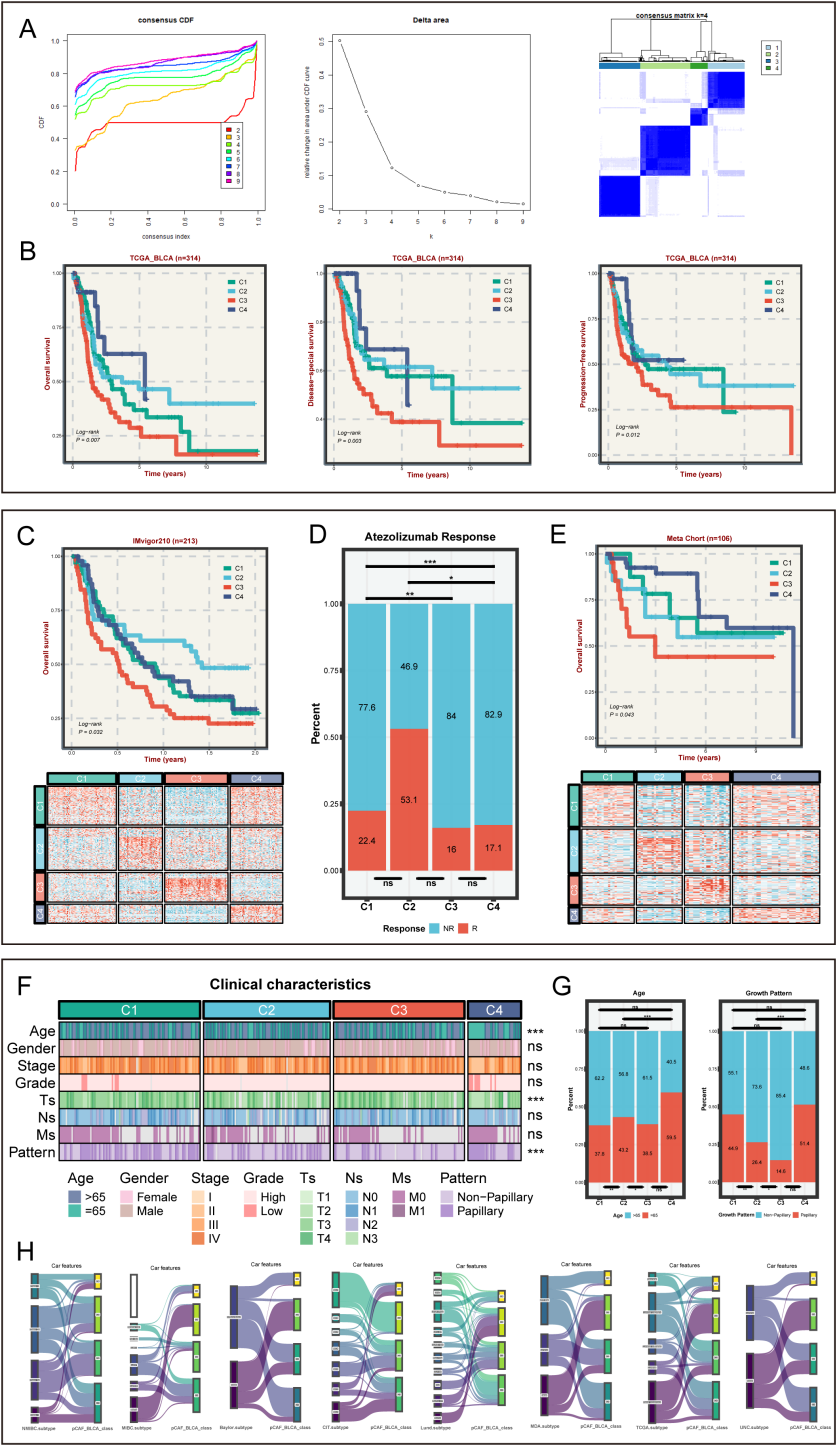
The identification and verification of pCAFs-based subtypes in bulk cohorts and clinical characteristics for four subtypes. **(A)** Identifying four heterogeneity subtypes through consensus clustering. **(B)** OS, DSS, and PFS among four subtypes in TCGA-BLCA cohort. **(C)** OS for four subtypes and NTP results in IMvigor210-cohort. **(D)** The distribution plots of immunotherapy sensitivity among four subtypes in IMvigor210-cohort. **(E)** OS for four subtypes and NTP results in GEO Meta cohort. **(F)** The distinct clinical characteristics among four BLCA subtypes. **(G)** The distribution plots of age and tumor growth pattern among four subtypes in TCGA cohort. **(H)** Sankey diagram of pCAF-based BLCA classifications and previously described consensus molecular classifications, comprising NMIBC_class, MIBC_class, CIT.subtype, Lund.subtype, MDA.subtype, TCGA.subtype, and UNC.subtype. nsP > 0.05, *P < 0.05, **P < 0.01, ***P < 0.001.

Firstly, we obtained 1215 STMN1+ pCAF-specific expressed genes with adj_pval < 0.05 and log2FC > 1 (**Supplementary Table S1**), further screened 85 STMN1+ pCAF-specific prognosis-related genes (**Supplementary Table S2**), and finally obtained the expression of specific prognosis-related genes in TCGA-BLCA for clustering analysis. Consensus clustering was performed to define tumor subtypes, with sample similarity quantified by proximity metrics. The optimal cluster number (k=4) was determined by convergence of the delta area plot and consensus cumulative distribution function (CDF) curve (**Figure 2A**), resulting in the identification of four pCAF-based BLCA subtypes in the TCGA cohort. A total of 98 patients were assigned to C1 (31.2%), 88 to C2 (28.0%), 91 to C3 (29.0%), and 37 to C4 (11.8%). **Figure 2B** showed that the survival analysis was statistically significant (n=314, OS, P = 0.007; DSS, P = 0.003; PFS, P = 0.012). C3 subtype demonstrated poor survival outcomes. Conversely, C4 was characterized by superior survival outcomes and a lower recurrence rate. Next, we extracted the characteristic genes for each subtype, namely 90 genes for C1, 86 for C2, 54 for C3, and 51 for C4 (**Supplementary Table S3**). Subsequently, we verified the classification capabilities of these characteristic genes in multiple external cohorts using NTP. Similarly, in the IMvigor210 cohort, four subtypes can be clearly distinguished. C3 displayed the worst prognosis and C4 showed superior prognosis (n=213, P = 0.032, **Figure 2C**), in agreement with the aforementioned results, validating the accuracy and consistency of the classification. C3 with the worst prognosis had the highest proportion of non-responders to atezolizumab in the IMvigor210 cohort. Interestingly, C2 exhibited the most favorable response to atezolizumab in the IMvigor210 cohort, suggesting that C2 may be more sensitive to chemotherapy (**Figure 2D**). Likewise, in the meta-cohort (n=106, P = 0.043, **Figure 2E**), GSE13507 (n=81, P = 0.019, **Supplementary Figure S2A**), GSE48075 (n=39, P = 0.016, **Supplementary Figure S2B**), and E−MTAB−4321 (n=219, P < 0.01, **Supplementary Figure S2C**), the survival analysis showed a comparable trend with the training cohort, confirming the rationality of the subtyping. Collectively, the robustness and generalization of pCAF-based BLCA classification were validated by large-scale RNA-seq data.

### 3.3 Comparisons of clinicopathological characteristics across four pCAF subtypes

Clinical characteristics from the TCGA-BLCA cohort were analyzed to assess the clinical significance of the four subtypes. Age, Ts, and growth pattern varied significantly across the four subtypes (P < 0.05, **Figures 2F, G** and **Supplementary Figure S2D**). The C3 subtype with the worst prognosis had the highest proportion of patients greater than 65 years of age and was associated with a higher proportion of non-papillary growth patterns (**Figure 2F**). Moreover, the proportion of C3 subtype at stage T1/T2 was the lowest (**Figure 2G**). These findings may be related to the worst prognosis of C3 subtype. To evaluate the correlation between our classification and other BLCA consensus classifiers, the pCAF-based BLCA classification of 314 samples was compared with various classifiers (**Figure 2H**). As expected, CAFs-based BLCA subtypes have limited overlap with other classifications, indicating the novelty of our subtypes and their complementary role to previous BLCA classifications.

### 3.4 Identification of cell death-related modalities among four BLCA subtypes

**Figure 3:**
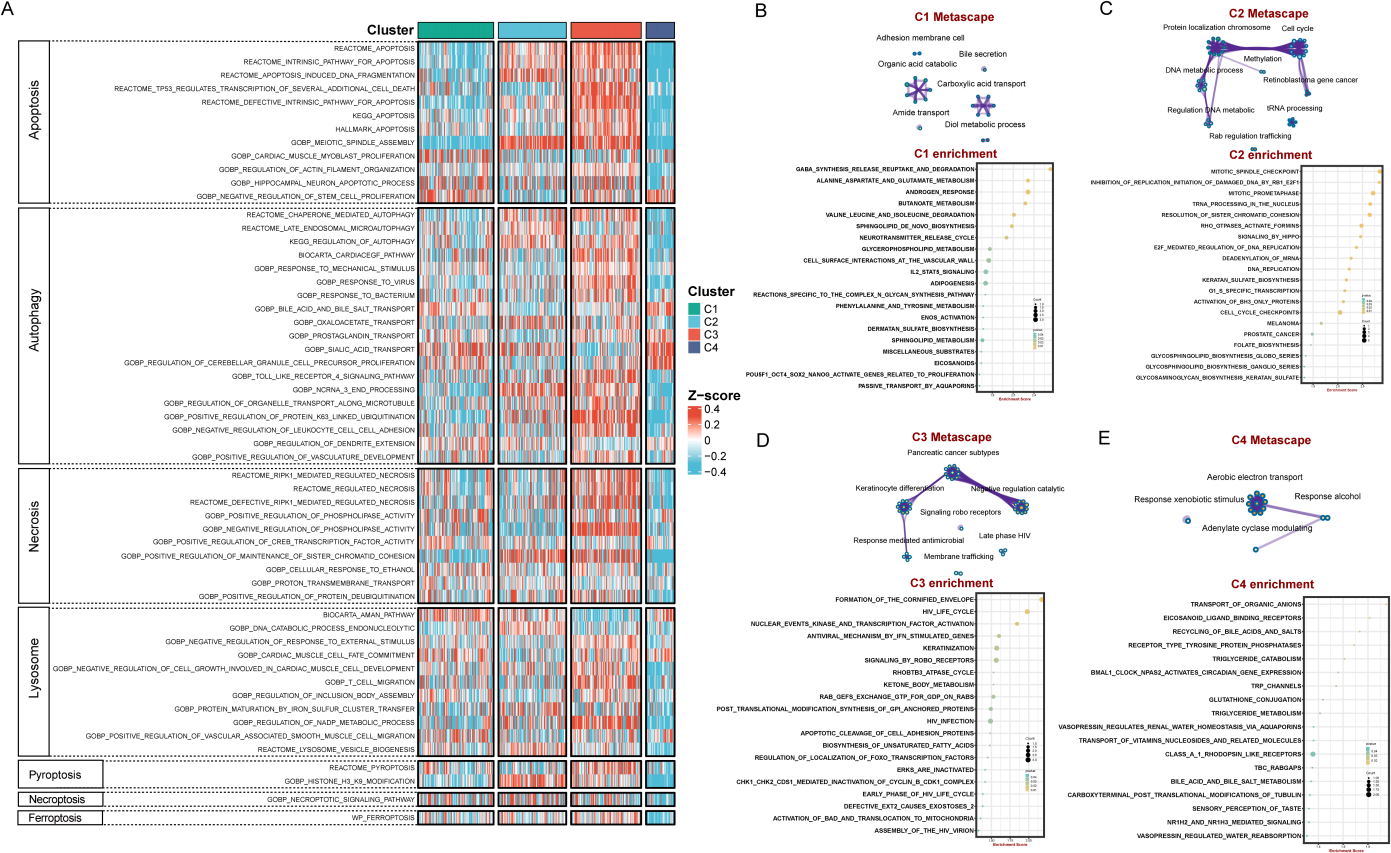
Distinctive cell death-related modalities and biological features among four BLCA subtypes. **(A)** Heatmap demonstrated differences in cell death signaling pathways among the four subtypes. Enrichment map network and KEGG enrichment analysis in C1 subtype **(B)**, C2 subtype **(C)**, C3 subtype **(D)**, C4 subtype **(E)**.

As is well documented, cell death significantly affects tumor cell proliferation, invasion, metastasis, and the efficacy of chemotherapy, thereby influencing the prognosis of BLCA (25–27). The results showed that most of cell death-related pathways were significantly up-regulated in the C3 subtype, while most of these pathways were only weakly enriched in C4 (**Figure 3A**), suggesting that these cell death-related pathways are closely associated with prognosis of BLCA. The expression levels of C1 and C2 in the cell death-related pathways were located between C3 and C4. The hypothesis can be drawn that the enrichment of stronger cell death pathways may promote tumor malignancy (28).

### 3.5 Distinctive biological features of four subtypes

Functional enrichment analyses revealed that C1 is mainly enriched in molecular metabolism-related pathways such as gaba_synthesis_release_reuptake_and_degradation, alanine_aspartate_and_glutamate_metabolism, and butanoate_metabolism (**Figure 3B**). C2 are mainly enriched in cell cycle and DNA repair pathways, exhibiting concerted activation of genome instability pathways, including mitotic_spindle_checkpoint, inhibition_of_replication_initiation_of_damaged_DNA_by_rb1_e2f1, mitotic_prometaphase, trna_processing_in_the_nucleus, and resolution_of_sister_chromatid_cohesion (**Figure 3C**). C3 subtype identified unique enrichment in keratinization and immune-modulatory pathways such as formation_of_the_cornified_envelope and antiviral_mechanism_by_ifn_stimulated_genes (**Figure 3D**). C4 subtype are mainly enriched in substance transport and metabolism pathways, including transport_of_organic_anions, eicosanoid_ligand_binding_receptors, recycling_of_bile_acids_and_salts, and triglyceride_catabolism (**Figure 3E**). Overall, the C1-C4 subtypes exhibit different bio-functional states.

### 3.6 Immune landscape for the four pCAF-based BLCA heterogeneity subtypes

**Figure 4:**
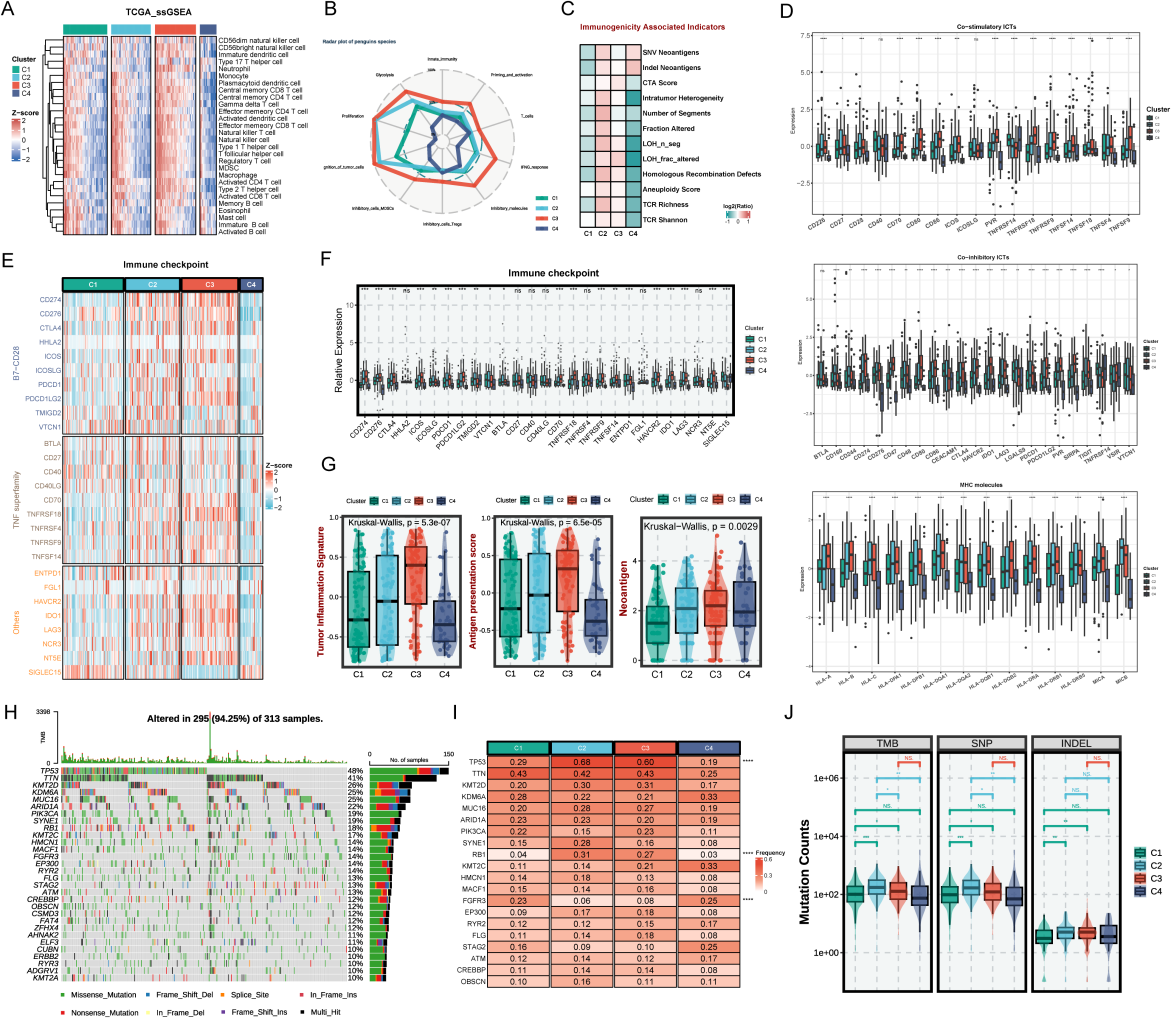
The immune landscape across distinct BLCA subtypes. **(A)** Heatmap of immune cell infiltration by SsGSEA in four subtypes. **(B)** Radar plot of immune gene expression levels. **(C)** Comparison of immune indicators in the four subtypes. **(D)** The relative expression of co-stimulatory and co-inhibitory immune check points and specific MHC molecules. **(E)** Heatmap of immune checkpoints of four BLCA subtypes. **(F)** The relative expression of immune checkpoints of the four subtypes. **(G)** The tumor inflammation signature (TIS) score, antigen presentation score (APS), and neoantigen in BLCA subtypes. **(H)** Mutation landscape in TCGA BLCA cohort. **(I)** Expression heatmap of the top 20 highly mutated genes in the four subtypes. **(J)** Tumor mutational burden, single nucleotide polymorphism, and insertion-deletion in BLCA subtypes. ns P > 0.05, *P < 0.05, **P < 0.01, ***P < 0.001, ****P < 0.0001.

Considering that the tumor immune microenvironment plays a key role in BLCA progression, we comprehensively explored the infiltration levels of immune cell subsets and the expression of various immune-related markers. Cibersort and ssGSEA was performed to assess the infiltration abundance of different immune cells across four subtypes (**Figure 4A**, **Supplementary Figures S2E, F**). The degree of infiltration of immune cells, including natural killer cells, CD8+ cells, CD4+ cells, neutrophil, regulatory T cells, and myeloid-derived suppressor cells (MDSCs), is the highest in the C3 subtype. The C4 subtype had the lowest degree of immune infiltration among the four subtypes. Immuno-radargrams further demonstrated that C3 is an immune-infiltrating tumor and C4 is an “immune-desert” tumor (**Figure 4B**). We further compared the immunogenicity-associated indicators of the four subtypes to further explore the characteristics of the immune microenvironment (**Figure 4C**). C2 subtype had higher immunogenicity and higher genomic instability, such as single-nucleotide variant (SNV) neoantigens, indel neoantigens, loss of heterozygosity (LOH) segments, LOH fraction alterations, and homologous recombination defects, accompanied by a higher T-cell receptor (TCR) richness (TCR richness and Shannon). The C1 and C4 subtypes have lower levels of these indicators, which may reflect poor survivor outcomes. Nevertheless, C4 had higher SNV neoantigens and Indel neoantigens, suggesting that C4, as an immune-desert tumor, has higher neoantigens at the same time, but fails to elicit an effective immune response.

Next, we explore the characteristics of the immune molecules of the four subtypes. C2 and C3 showed increased activation of co-stimulatory immune checkpoint inhibitors (ICIs) such as CD226, CD27, CD28, and TNFRSF14, and co-inhibitory receptors such as CD160, CD244, CD48, CD274, CD276, LAG3, LGALS9, and CTLA4, MHC molecules like HLA-A/B/C and HLA-DQA1/2 (**Figure 4D**). However, expressions of immune checkpoints were scarce in the C1 and C4 subtypes, suggesting that ICI therapy might not be effective (**Figures 4E, F**). Furthermore, antigen presentation efficiency was further assessed using the antigen processing and presenting machinery score (APS) (**Figure 4G**). It has been demonstrated in numerous studies that tumor inflammation signatures (TIS), APS, and neoantigen score are all predictive biomarkers for tumor immunotherapy, where higher values generally correlate with better immunotherapy outcomes (24). Similarly, higher TIS, APS score, score, and neoantigen score further demonstrate that the C2 and C3 subtypes could achieve greater clinical efficacy with immunotherapy (**Figure 4G**).

Moreover, this study further explored the gene mutations in the four subtypes (**Figures 4H–J**). **Figure 4I** shows that C2 and C3 have significantly higher TP53 and RB1 mutation frequencies than C1 and C4. TP53 mutations could cause DNA damage repair defects and RB1 mutations could cause chromosome segregation errors, thereby promoting the development of multiple tumors. It was demonstrated that RB1 and TP53 co-mutations are strongly associated with genomic biomarkers of ICI response in urothelial BLCA (29). The higher mutation frequencies tend to portend a poor prognosis. However, studies have shown that high tumor mutational load (TMB) is associated with longer survival after ICI therapy (30). C2 and C3 have higher single-nucleotide polymorphism (SNP), insertion-deletion (INDEL), and TMB compared to C1 and C4, which further demonstrates the suitability of C2 and C3 for immunotherapy.

### 3.7 Identification and validation of hub genes for C1 subtypes

**Figure 5:**
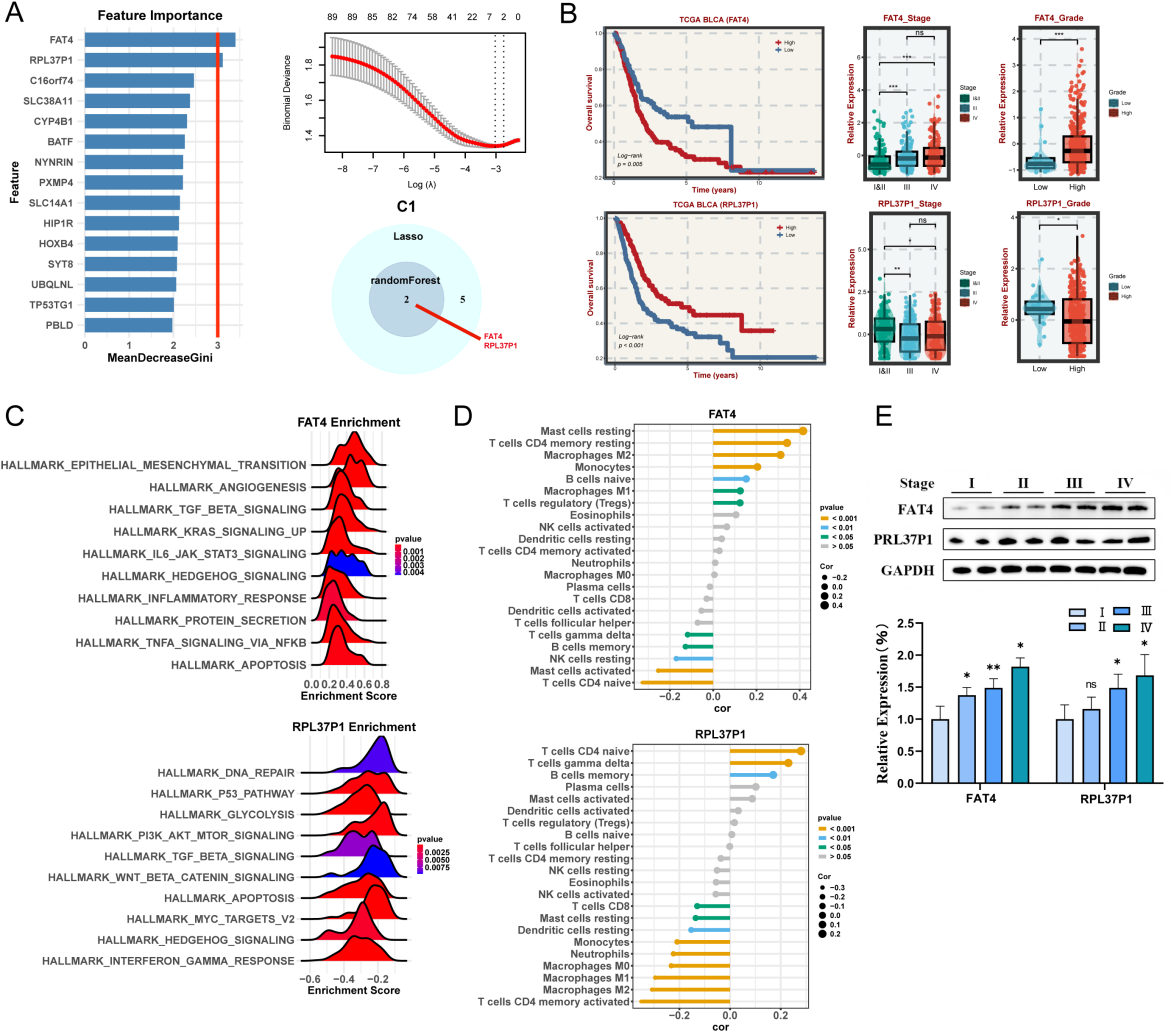
Machine learning algorithms to obtain hub targets of C1 subtype. **(A)** The identification of potential targets using the randomForest (left) and Lasso (right) algorithm. Venn diagram for identifying hub genes of C1 subtype. **(B)** Survival analysis in TCGA BLCA cohort, respectively, for the grouping of the median expression of FAT4 and RPL37P1. The relative expression of different stages (left) and grades (right) for FAT4 and RPL37P1, respectively. **(C)** GSEA enrichment analysis for FAT4 and RPL37P1 based on hallmark gene set. **(D)** Correlation between 22 infiltrating immune cells and FAT4 and RPL37P1, respectively. **(E)** WB and the relative expression of FAT4 and RPL37P1 proteins in different tumor stages. *P < 0.05, **P < 0.01, ***P < 0.001.

To explore the molecular mechanisms and prognosis-related markers of the four subtypes, machine-learning algorithms were used to identify hub genes. For C1 subtype, the randomForest algorithm analysis screened two potential hub genes using a node size threshold of 3. LASSO regression analysis finally screened five target genes. By taking the intersection of the genes identified by the two algorithms, we identified two key genes, FAT4 and RPL37P1, that are closely associated with the malignant progression of BLCA (**Figure 5A**). The survival curves illustrated that the high-expression FAT4 had a poor prognosis and the high-expression RPL37P1 had a favorable prognosis (**Figure 5B**). Moreover, significant differences were observed in the expression levels of hub genes across different stages, with higher expression levels of FAT4 in patients at higher stages and higher expression levels of RPL37P1 in patients at lower stages (**Figure 5B**). Likewise, high-grade BLCA patients had higher gene expression levels of FAT4 and lower gene expression levels of RPL37P1, further corroborating that FAT4 gene was positively correlated with the degree of BLCA malignancy and RPL37P1 gene was protective (**Figure 5B**). Next, their functions and correlations with immune cells were examined. FAT4 is predominantly enriched in pathways associated with driving malignant phenotypic transformation and microenvironmental remodeling such as HALLMARK_EPITHELIAL_MESENCHYMAL_TRANSITION, HALLMARK_ANGIOGENESIS, HALLMARK_TGF_BETA_SIGNALING, and HALLMARK_KRAS_SIGNALING_UP, while RPL37P1 is predominantly enriched in pathways related to metabolic reprogramming and genomic destabilization, such as HALLMARK_DNA_REPAIR, HALLMARK_P53_PATHWAY, and HALLMARK_GLYCOLYSIS (**Figure 5C**). Moreover, FAT4 was significantly positively correlated with mast cells resting, T cells CD4 memory resting, Macrophages M2, and monocytes. RPL37P1 was significantly positively correlated with T cells CD4 naive, T cells gamma delta, and B cells memory, closely related to immune response (**Figure 5D**). The results of Western blot analysis showed that higher stages were correlated with higher gene expression levels of FAT4. However, the relationship between RPL37P1 expression level and staging was not significant (**Figure 5E**).

### 3.8 Identification and validation of hub genes for C2 subtypes

**Figure 6:**
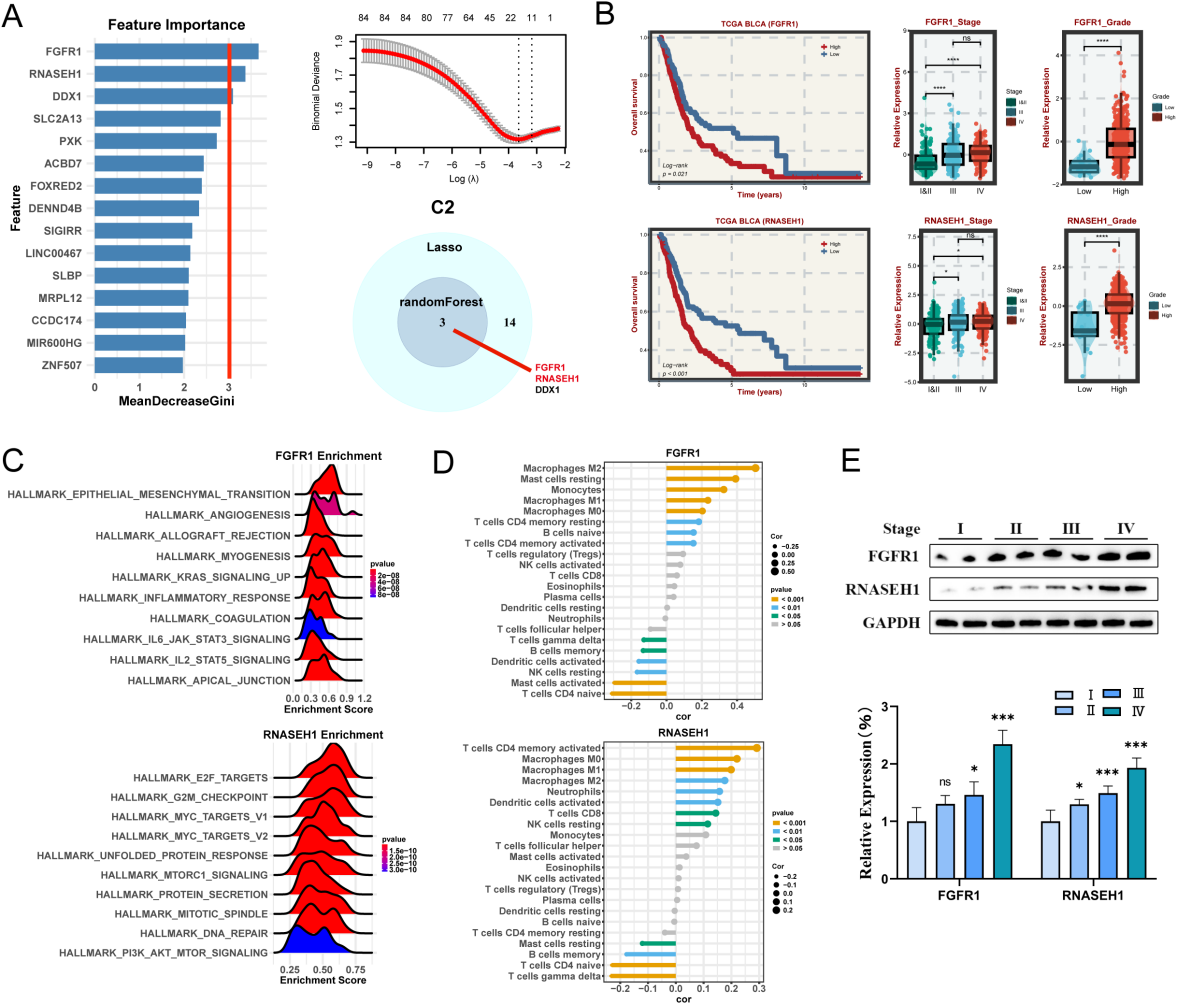
Machine learning algorithms to obtain hub targets of C2 subtype. **(A)** The identification of potential targets using the randomForest and Lasso algorithm. Venn diagram for identifying hub genes of C2 subtype. **(B)** Survival analysis in TCGA BLCA cohort, respectively, for the grouping of the median expression of FGFR1 and RNASEH1. The relative expression of different stages and grades for FGFR1 and RNASEH1. **(C)** GSEA enrichment analysis for FGFR1 and RNASEH1 based on hallmark gene set. **(D)** Correlation between 22 infiltrating immune cells and FGFR1 and RNASEH1, respectively. **(E)** WB and the relative expression of FGFR1 and RNASEH1 proteins in different tumor stages. *P < 0.05, **P < 0.01, ***P < 0.001.

For C2 subtype, randomForest algorithm analysis screened three potential hub genes and LASSO regression analysis screened 14 target genes, finally identifying three key genes, namely FGFR1, RNASEH1, DDX1 (**Figure 6A**). Among them, the two hub genes FGFR1 and RNASEH1, are associated with survival in BLCA patients. The KM curves illustrated that the high-expression FAT4 and RNASEH1 had poor prognosis (**Figure 6B**) and patients with advanced stages and grades have higher levels of gene expression of FGFR1 and RNASEH1 (**Figure 6C**), indicating that these genes were malignant and protumorigenic. Likewise, FGFR1 was also predominantly enriched in pathways associated with malignant phenotypic transformation and microenvironmental remodeling, while RNASEH1 was predominantly enriched in pathways related to genome stability (**Figure 6C**). Moreover, FAT4 and RNASEH1 were significantly positively correlated with macrophages (**Figure 6D**). The results of western blot analysis showed that higher stages were correlated with higher gene expression levels (**Figure 6E**).

### 3.9 Identification and validation of hub genes for C3 subtypes

**Figure 7:**
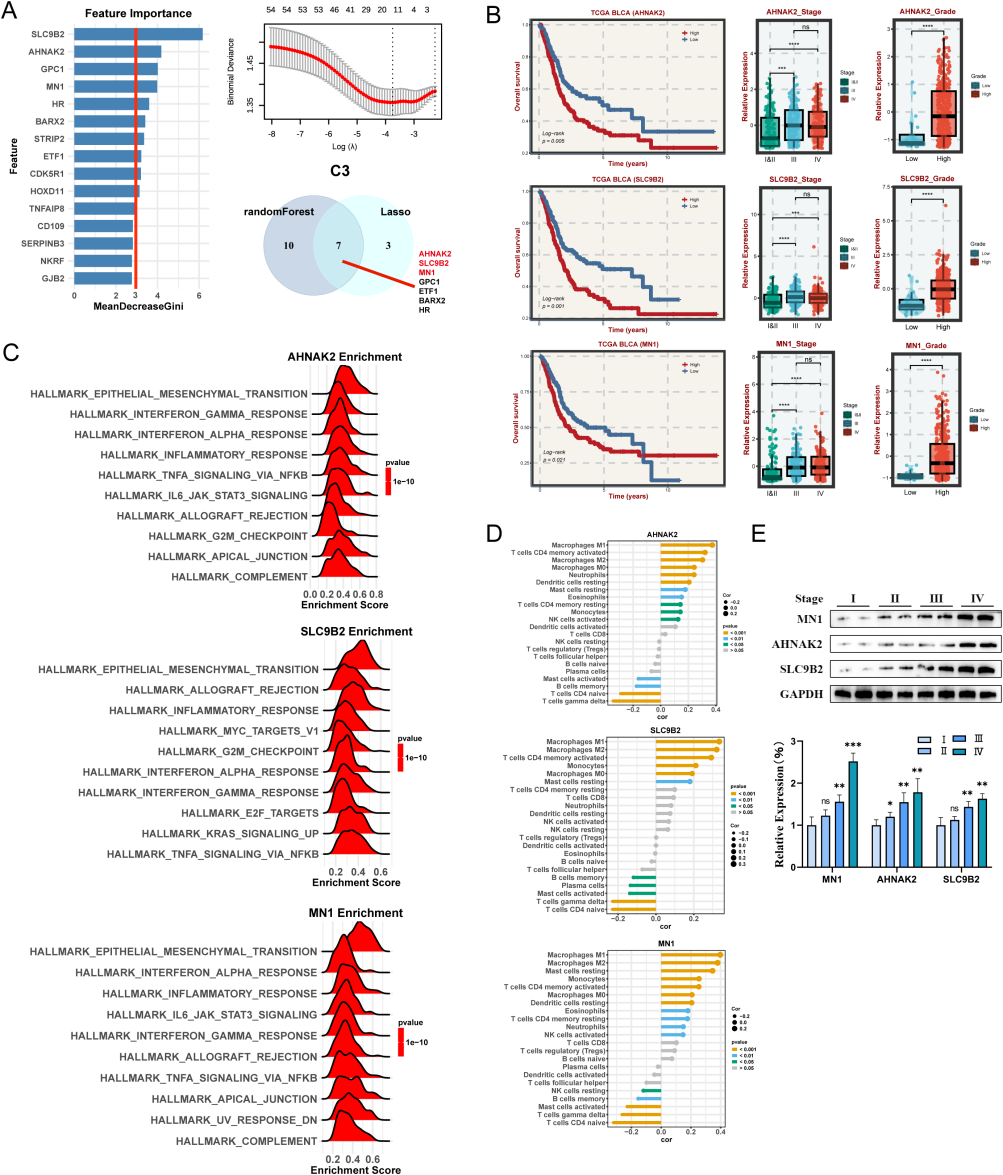
Machine learning algorithms to obtain hub targets of C3 subtype. **(A)** The identification of potential targets using the randomForest and Lasso algorithm in C3 subtype. **(B)** Survival analysis for AHANK2, SLC9B2, and MN1. The relative expression of different stages and grades for AHANK2, SLC9B2, and MN1, respectively. **(C)** GSEA enrichment analysis for AHANK2, SLC9B2, and MN1 based on hallmark gene set. **(D)** Correlation between 22 infiltrating immune cells and AHANK2, SLC9B2, and MN1, respectively. **(E)** WB and the relative expression of AHANK2, SLC9B2, and MN1 proteins in different tumor stages. ns P > 0.05, *P < 0.05, **P < 0.01, ***P < 0.001, ****P < 0.0001.

Similarly, three key genes (AHNAK2, SLC9B2, and MN1) in C3 subtype were identified (**Figure 7A**). The high-expression of these genes is strongly associated with a poor prognosis (**Figure 7B**). What’s more, these genes are expressed at higher levels in advanced and highly graded patients (**Figure 7B**). Interestingly, They are all predominantly enriched in HALLMARK_EPITHELIAL_MESENCHYMAL_TRANSITION and are closely related to Macrophages (**Figures 7C, D**). Western blot analysis demonstrated a progressive increase in the expression of these genes with staging (**Figure 7E**).

### 3.10 Identification and validation of hub genes for C4 subtypes

**Figure 8:**
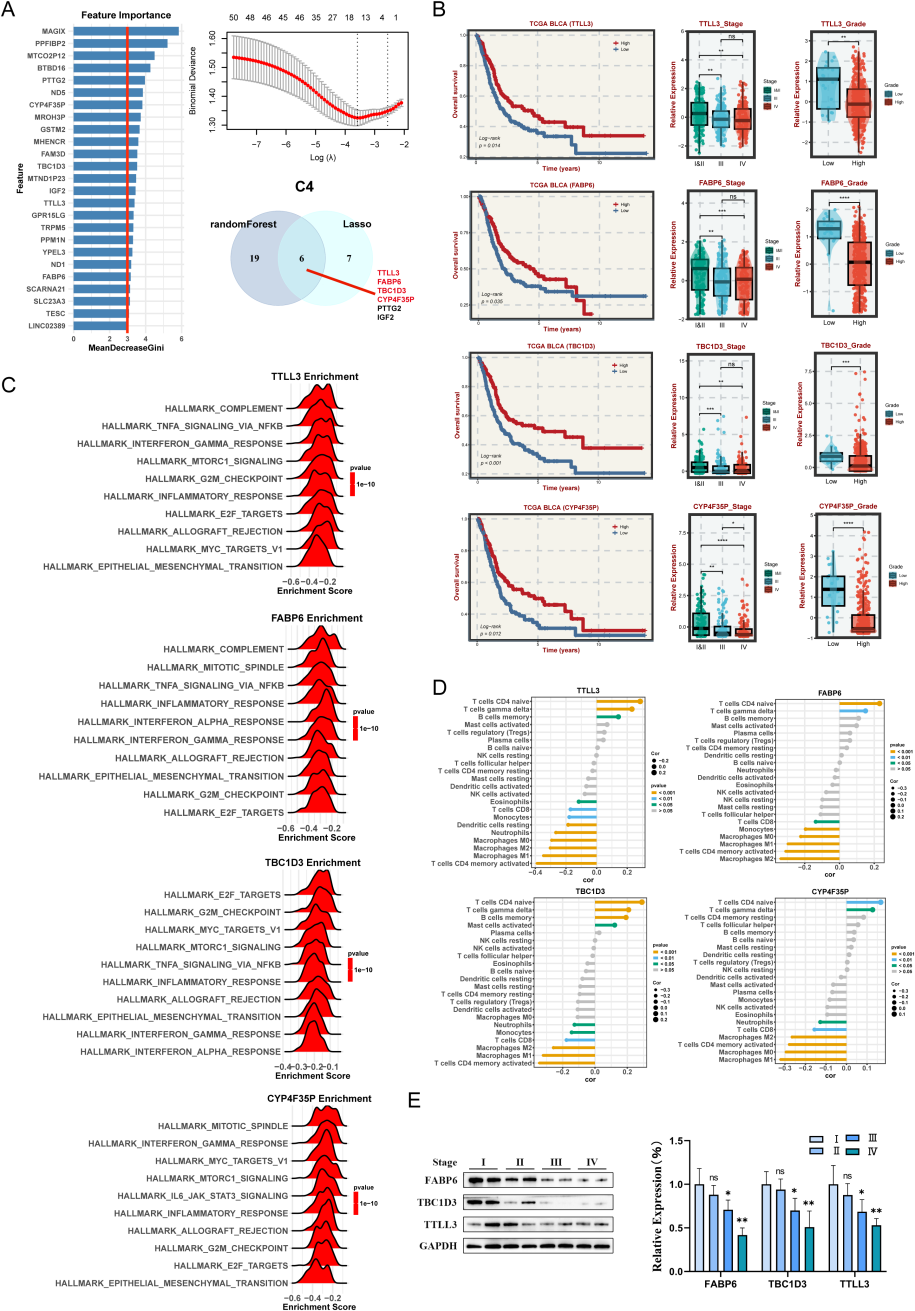
Machine learning algorithms to obtain hub targets of C4 subtype. **(A)** The identification of potential targets. **(B)** Survival analysis in TCGA-BLCA cohort for the TTLL3, FABP6, TBC1D3, and CYP4F35P. The relative expression of different stages and grades for TTLL3, FABP6, TBC1D3, and CYP4F35P. **(C)** GSEA enrichment analysis for TTLL3, FABP6, TBC1D3, and CYP4F35P. **(D)** Correlation between 22 infiltrating immune cells and TTLL3, FABP6, TBC1D3, and CYP4F35P, respectively. **(E)** WB and the relative expression of TTLL3, FABP6, TBC1D3, and CYP4F35P proteins in different tumor stages. ns P > 0.05, *P < 0.05, **P < 0.01, ***P < 0.001, ****P < 0.0001.

In C4 subtype, this study illustrated that the high expression of four key genes (TTLL3, FABP6, TBC1D3, and CYP4F35P) are associated with the favorable prognosis in the TCGA cohort (**Figures 8A, B**). These genes are more lowly expressed in patients with high staging and advanced grade (**Figure 8B**). Enrichment analysis indicated that Each gene has a different enrichment function and are all closely related to T cells CD4 naive and T cells gamma delta (**Figures 8C, D**). To further validate the aforementioned results, the expression levels of three hub genes (No CYP4F35P antibody) were quantified across the four different stages of BLCA tissues (**Figure 8E**). Taken together, we identified the corresponding hub genes for each subtype. The specific role of these genes remains to be validated in further clinical practice.

## 4 Discussion

CAFs have been shown to have various cellular communications with tumor cells and immune cells, which not only secrete cytokines and mediate extracellular matrix remodeling to indirectly affect tumor cells and immune cells, but also promote tumor cell proliferation, invasion, and metastasis through direct cell-to-cell interactions (3, 7, 8, 31), further promoting immune escape, chemotherapy resistance, and tumor recurrence. This study identified CAFs closely associated with BLCA development based on scRNA-seq datasets. Through further systematic clustering and functional analysis of CAFs, we successfully identified 10 distinct CAF sub-clusters. The novel pCAF-based BLCA classification system was subsequently developed and rigorously validated using different datasets of tumor bulk RNA-seq data with excellent robustness and generalizability.

The four CAF-based BLCA subtypes have different molecular, functional and immunological characteristics. The C3 subtype exhibits a malignant phenotype with poor clinical prognosis, with a higher enrichment of the cell death pathway, a significant immune infiltration, and higher expression of MHC molecules and co-stimulatory/co-inhibitory molecules, suggesting that C3 is an immune-enriched phenotype and may be sensitive to immunotherapy. The higher TIS, APS, neoantigen score, and TMB further implied that C3 was potentially responsive to immunotherapy. Moreover, some studies indicate that cell death signals play an important role in the immune environment, and targeting cell death signaling pathways may enhance the efficacy of tumor immunotherapy (28, 32). Therefore, it is possible that cell death inducers and immunotherapy used together will achieve better results for C3. In contrast, the C4 subtype has a smaller population and a better prognosis, as characterized by a lower enrichment of cell death pathways, a lower frequency of tumor mutations, and an ‘immune desert’ TME. C4 had lower expression levels of immune molecules, APS, and TMB, further suggesting resistance to immunotherapy. C1 subtype was mainly enriched in metabolism-related pathways, whereas C2 subtype was mainly enriched in genome instability pathway activation, with a higher frequency of mutations and better response to atezolizumab treatment. The expression levels of cell death-related pathways and the degree of immune infiltration of C1 and C2 were intermediate between C3 and C4. Collectively, CAF-based BLCA classification effectively distinguished independent BLCA groups with low overlap with existing transcriptional classification methods, demonstrating unprecedented predictive value. Besides, this study identified two potential key genes (FAT4 and RPL37P1) as significantly associated with C1, two potential target genes (FGFR1 and RNASEH1) for C2, three potential target genes (AHNAK2, SLC9B2, and MN1) for C3, and four potential target genes (TTLL3, FABP6, TBC1D3 and CYP4F35P) for C4. These hub genes play critical roles in BLCA patients and provide potential molecular targets and clinical strategies for designing targeted therapeutic regimens for BLCA patients.

FAT4 (FAT atypical cadherin 4) is a member of the cadherin family and is involved in cell adhesion, epithelial-mesenchymal transition (EMT), and the regulation of the TME (33–36). It has been verified that FAT4 could affect tumor metastasis and regulate TMB and microsatellite instability to influence the immunotherapy response through calcium signaling pathways and chemokine signaling pathways (34). Additionally, the interaction between FAT4 and CAFs could reshape the TME and promote BLCA progression (34, 35). Likewise, in our study, FAT4 shows that high expression is associated with poor prognosis. RPL37P1 (Ribosomal Protein L37 pseudogene 1) is a pseudogene related to ribosome biogenesis (37). Its parent gene, RPL37, plays a crucial role as a ribosomal protein in protein translation and participates in regulating the tumor suppression (38). RPL37 can bind and inhibit the E3 ubiquitin ligase activity of MDM2, thereby stabilizing p53 and inducing cell cycle arrest or apoptosis (38), which is closely related to the prognosis of patients in various cancers. Moreover, DNA damage (such as exposure to ultraviolet rays or treatment with cisplatin) can trigger the proteasomal degradation of RPL37, and then activate p53 pathway through a L11-dependent manner, indicating that RPL37 and its pseudogene may be involved in the DNA damage stress response (39). In summary, FAT4 and RPL37P1 play important roles in reprogramming the TME and have the potential to become a cancer prognostic marker and therapeutic target for C1 subtype.

FGFR1 (fibroblast growth factor receptor 1) is a transmembrane receptor with tyrosine kinase activity, belonging to the FGFR family (FGFR1-4) (40). FGFR1 regulates cell proliferation, differentiation, and migration by activating downstream signaling pathways (such as MAPK/PLCγ and PI3K/AKT) (41, 42). Overexpression or abnormal splicing of FGFR1 can promote EMT, enhance invasion and metastasis ability, and drive cell proliferation through MAPK/ERK or COX-2 pathways, which is closely related to tumor malignancy progression (43–45). It has been found that FGFR1 overexpression is an independent factor for poor prognosis in patients with MIBC, and is associated with shorter recurrence time and overall survival (43). Moreover, the abnormal activation of FGFR1 can be further strengthened by collaborating with molecules like TTYH3, thereby reinforcing downstream oncogenic signals (46). Although pan-FGFR inhibitors like erdafitinib are effective for BLCA with FGFR3 mutations/fusions, their efficacy is limited for FGFR1-dependent tumors (44, 47). RNASEH1 (Ribonuclease H1) is a nucleolytic endonuclease that specifically degrades RNA-DNA hybrids (R-loops), which plays a crucial role in maintaining genomic stability, regulating DNA replication and repair (48–50). RNASEH1 could prevent replication stress and DNA damage by removing R-loops, and dysfunction of RNASEH1 is closely related to the progression of various cancers, including gastric cancer, B-cell lymphoma, and melanoma (51–54). Some cancer cells may rely on the telomerase-independent ‘Alternative Lengthening of Telomeres’ pathway to maintain telomere length (49). RNASEH1 regulates the balance of hybrid structures between telomere DNA and long non-coding RNA TERRA, affecting homologous recombination efficiency. Abnormal expression of RNASEH1 may lead to telomere dysfunction or excessive recombination, promoting the immortalization and invasiveness of cancer cells. A pan-cancer analysis revealed that overexpression of RNASEH1 is associated with the regulation of the TME and poor prognosis, suggesting that RNASEH1 may promote BLCA immune escape by inhibiting anti-tumor immunity (48). Our study also showed that high expressions of FGFR1 and RNASEH1 related to poor prognosis. In conclusion, FGFR1 and RNASEH1 may affect BLCA progression through multiple mechanisms and may become prognostic markers or therapeutic targets for BLCA.

AHNAK2 is a large molecular nuclear protein with a molecular weight exceeding 600 kDa, belonging to the AHNAK protein family, regulating tumor progression by activating signaling pathways such as ERK, MAPK, Wnt, and MEK, as well as promoting EMT (55). AHNAK2 is abnormally highly expressed in numerous cancers and is closely associated with poor prognosis (55, 56). Several researchers have revealed that high expression of AHNAK2 is significantly correlated with the malignancy of BLCA (56–58). For patients who underwent radical cystectomy and have high AHNAK2 expression in tumor tissues, their disease-free survival and cancer-specific survival were significantly shortened, and multivariate analysis confirmed that AHNAK2 was an independent poor prognostic factor (56). From the molecular mechanism perspective, the expression of AHNAK2 in the tumor hypoxic microenvironment is regulated by HIF-1α, which may drive tumor progression by inducing EMT and enhancing tumor stem cell characteristics (59). It is noteworthy that the level of AHNAK2 protein in the urine of BLCA patients is significantly elevated, especially in MIBC, making it a potential non-invasive diagnostic marker (58). SLC9B2 (also known as NHA2) is a unique Na/H antiporter, belonging to the SLC9B subfamily, with 14 transmembrane segments and forming a unique lipid-sensitive dimer structure (60, 61). Nevertheless, there are relatively few direct studies on SLC9B2 in BLCA. In terms of physiological functions, SLC9B2 is highly expressed in the distal convoluted tubules of the kidney, participating in blood pressure homeostasis and electrolyte balance by regulating the WNK4-NCC signaling pathway (62). Interestingly, SLC9B2 exhibits abnormal expression in various pathological conditions. In polycystic kidney disease, SLC9B2 is significantly upregulated and positively correlated with cyst size, regulated by the polycystin-1/Ca2+/NFAT signaling axis, which can be induced by vasopressin and methylxanthine drugs (63). What’s more, SLC9B2 is related to the pathogenesis of diabetes and hypertension, and is crucial for sperm motility and fertility (64, 65). The MN1 (Meningioma 1) gene is an important transcriptional co-regulatory factor that is initially discovered in meningiomas and has been confirmed to be involved in the occurrence and development of various malignant tumors in recent years (66, 67). MN1 enhances mRNA stability through m^6^A methylation modification mediated by METTL14, promoting tumor progression and chemotherapy resistance in osteosarcoma (68). In astrocytoma and other nervous system tumors, MN1 gene rearrangements (such as MN1-BEND2 and MN1-CXXC5) drive tumor occurrence *via* activating the PDGFRα signaling pathway (69). However, the high expression of MN1 in low-grade gliomas actually predicts a better prognosis, and this tissue-specific difference suggests that the regulatory network of MN1 is highly complex (66). Of note, MN1 exerts a pro-tumor effect through the XIST/miR-15a-5p/MN1/FZD2 signaling axis, and its significantly high expression is observed in female patients with poor prognosis, which may be an important molecular basis for the gender difference in BLCA (70). The expression regulation of MN1 may involve epigenetic regulation of long non-coding RNAs, and the activation of its downstream effector molecule FZD2 may affect the remodeling of TME (70). In C3, high expression of AHNAK2, SLC9B2, and MN1 is correlated with poor survival and may be potential therapeutic targets. These findings provide a novel perspective for understanding the molecular mechanism underlying the poor prognosis of BLCA.

TTLL3 is a tubulin glycine ligase that regulates the assembly of cilia, which plays a crucial role in maintaining the structure and function of cilia (71). Studies have shown that TTLL3 is expressed in colon tissues, and its absence or decreased levels of its expression lead to the deficiency of tubulin glycosylation and a reduction in primary cilia, thereby promoting the development of colorectal cancer (72). FABP6 (Fatty Acid Binding Protein 6) is an intracellular lipid transporter protein, which precisely regulates metabolic pathways, signal transduction, and gene expression (73). It is abnormally expressed in various cancers and is closely related to the occurrence and progression of cancer. In colorectal cancer, FABP6 is negatively correlated with immune infiltration, and its downregulation can enhance tumor immunogenicity and promote the recruitment of CD8+ T cells (74). FABP6 is regulated by the transcription factor REST and participates in gastric cancer progression by influencing autophagy and the Akt/mTOR pathways (75). Knockdown of FABP6 can significantly inhibit the proliferation and motility of BLCA cells, while downregulating the expression of cell cycle proteins such as CDK2 and CDK4 and blocking the AKT-mTOR signaling pathway, enhancing the therapeutic effect of cisplatin (76). Interestingly, some researchers indicate that FABP6 has been identified as a protective gene in the prognostic model of BLCA, and its expression level is related to the survival period of patients (77). TBC1D3, a hominoid-specific oncogene belonging to the TBC1 domain protein family, is encoded by a cluster of paralogues located on chromosome 17q12 (78). TBC1D3 enhances cell migration ability in breast cancer by activating the TNFα/NF-κB signaling pathway and upregulating the expression of OLR1 (79). The oncogenic mechanism of TBC1D3 involves the regulation of multiple key signaling pathways, including maintaining the stability of EGFR through interaction with the microtubule network, delaying EGFR degradation, and enhancing Ras activity to promote cell proliferation, as well as affecting gene expression through epigenetic regulation (80, 81). However, there are relatively few direct studies on TBC1D3 in BLCA. CYP4F35P is an lncRNA derived from a pseudogene and belongs to the cytochrome P450 family (82, 83). In recent years, it has been found to have regulatory effects in various cancers. In laryngeal squamous cell carcinoma and tongue squamous cell carcinoma, CYP4F35P shows differential expression patterns and forms a co-expression network with genes such as MUC21 and CEACAM1, suggesting that it may affect tumor progression by regulating cell adhesion and signal transduction processes (83, 84). In BLCA, CYP4F35P has been identified as one of the seven key prognostic-related lncRNAs, and its expression characteristics are significantly correlated with the survival rate of patients (82). The risk scoring model based on it can effectively predict the prognosis of BLCA patients (82). These findings suggest that TTLL3, FABP6, TBC1D3, and CYP4F35P may become potential biomarkers for the diagnosis and prognosis assessment of BLCA. Future research needs to further verify the specific functional mechanism of these genes in BLCA and their potential value as a diagnostic marker and therapeutic target.

## 5 Limitations and conclusions

Several limitations need to be noted. Firstly, CAF clustering and CAF-based BLCA classification were constructed from retrospective data from public databases. And the inconsistent number of patient stage distribution and the lack of prior treatment history in these public databases further limited our study. Thus, future validation in more prospective, multi-center BLCA cohorts is needed. Secondly, we only initially explored the key genes for each subtype, and subsequent studies will need to delve deeper into their potential mechanisms of action in the development of BLCA. In conclusion, this study identified a promising platform for understanding CAF heterogeneity and BLCA classification, which could provide novel insights into the complex molecular mechanisms of BLCA. Each heterogeneous subtype possesses different unique molecular, functional and immunological characteristics, implying a different therapeutic strategy, further facilitating personalized medicine for BLCA patients.

## Data Availability

The original contributions presented in the study are included in the article/**Supplementary Material**. Further inquiries can be directed to the corresponding author.

## Supplementary material

Supplementary Figure 1 Identification of CAFs in BLCA.

Supplementary Figure 2 The verification of CAFs-based BLCA subtypes in bulk cohorts and the immune landscape across distinct BLCA subtypes.

Supplementary Table 1 STMN1+ pCAF-specific expressed genes.

Supplementary Table 2 STMN1+ pCAF-specific prognosis-related genes.

Supplementary Table 3 Marker_genes of four subtype.
