# Supplementary figures and images for: Characterization of cancer-related fibroblasts in bladder cancer and construction of CAFs-based bladder cancer classification: insights from single-cell and multi-omics analysis
Source: Front Immunol. 2025 Sep 11;16:1580986. doi: 10.3389/fimmu.2025.1580986 (PMC12461746; doi:10.3389/fimmu.2025.1580986)

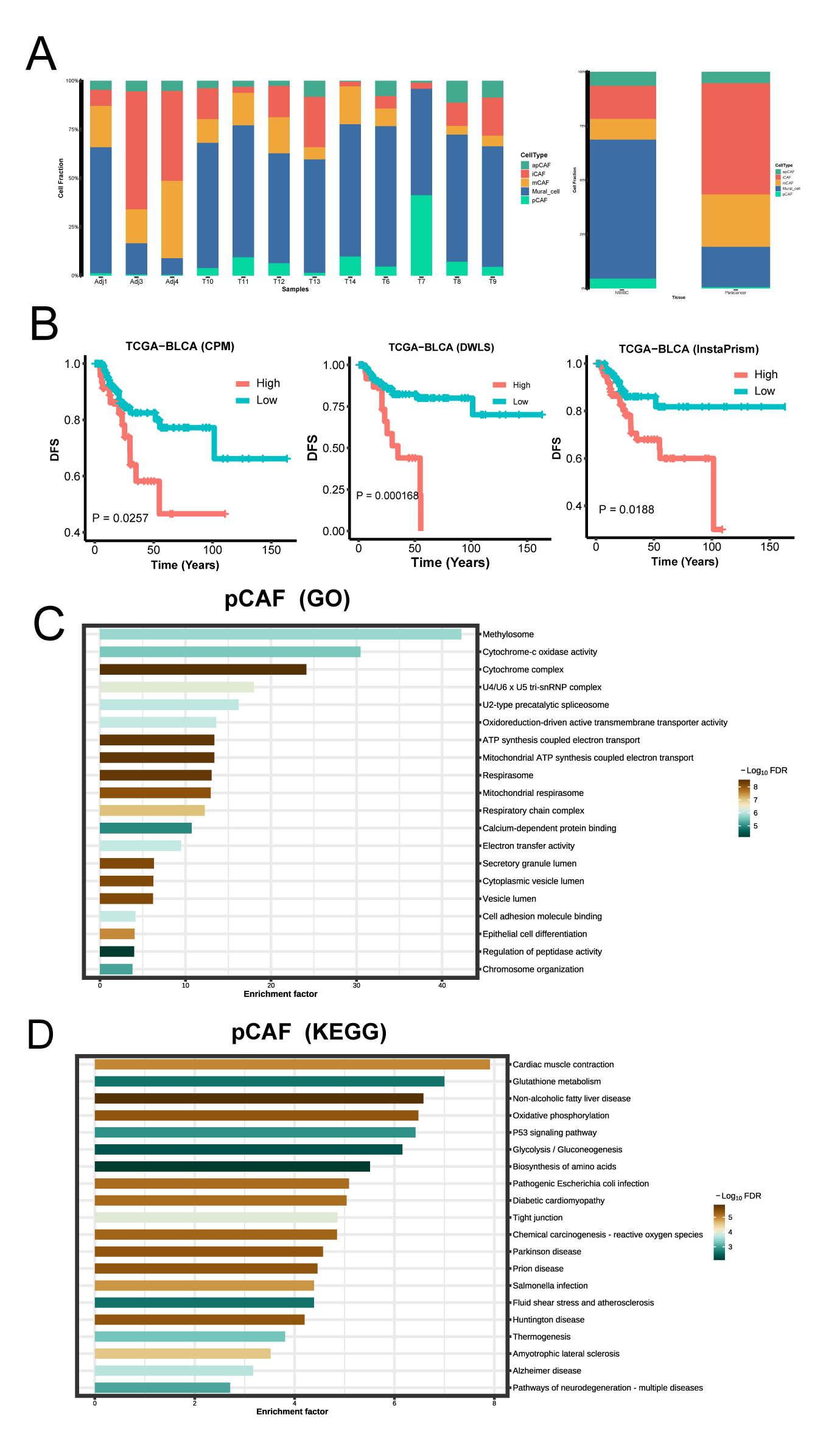

Supplement: Supplementary Figure 1 — Identification of CAFs in BLCA. [file Image1.tif]

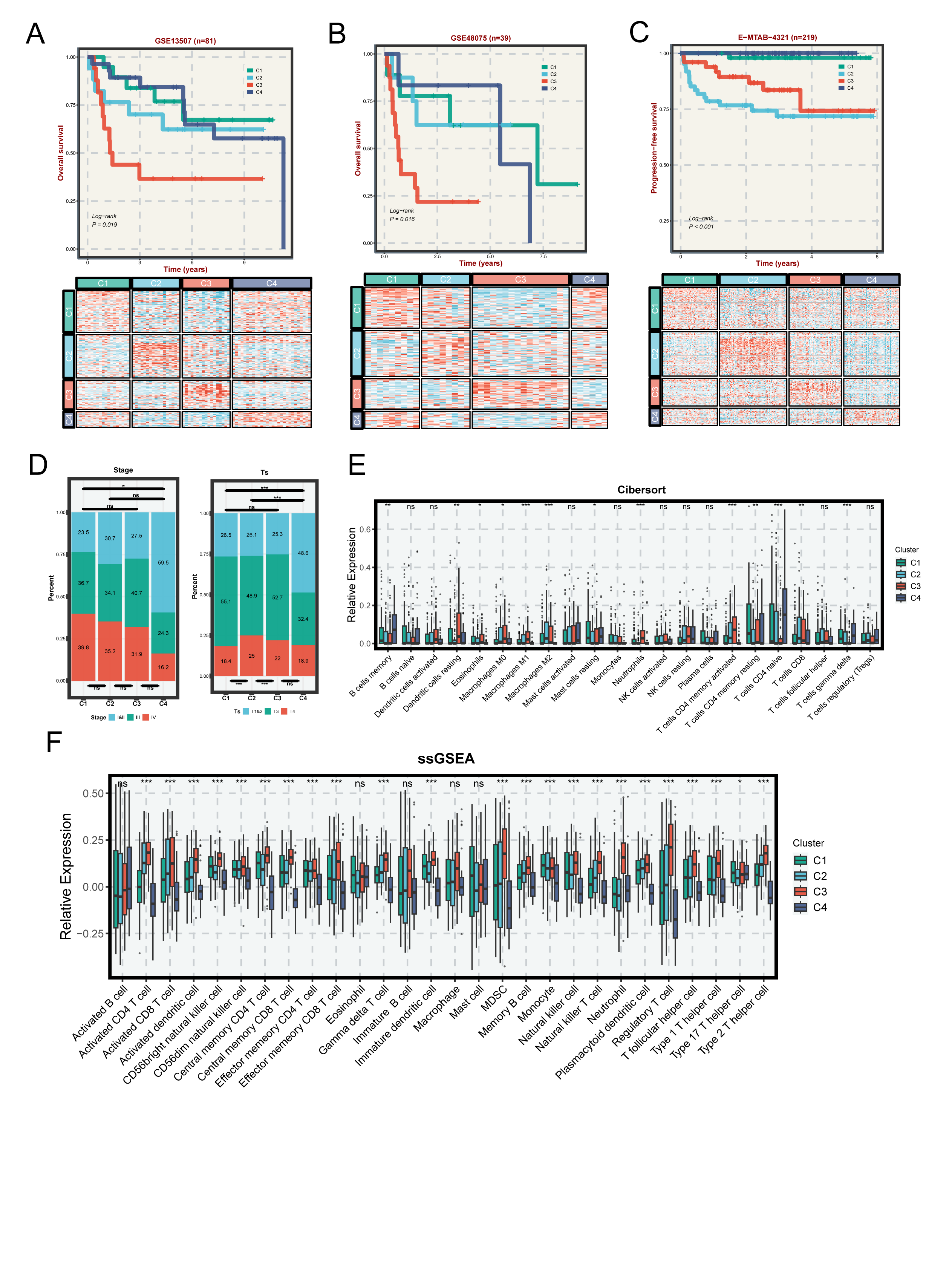

Supplement: Supplementary Figure 2 — The verification of CAFs-based BLCA subtypes in bulk cohorts and the immune landscape across distinct BLCA subtypes. [file Image2.tif]
