# Supplementary material for: Characterization of cancer-related fibroblasts in bladder cancer and construction of CAFs-based bladder cancer classification: insights from single-cell and multi-omics analysis
Source: Front Immunol. 2025 Sep 11;16:1580986. doi: 10.3389/fimmu.2025.1580986 (PMC12461746; doi:10.3389/fimmu.2025.1580986)
Supplement: Supplementary Table 1 — STMN1+ pCAF-specific expressed genes. [file Table1.docx]

**Characterization of cancer-related fibroblasts (CAFs) in bladder cancer and construction of CAFs-based bladder cancer classification: insights from single-cell and multi-omics analysis**

**Running title：**Subtypes of cancer-related fibroblasts in bladder cancer

Zhaokai Zhou^1^, Yajun Chen^2^, Zhan Wang^3^, Shuai Yang^4^, Zhengrui Li^5^, Run Shi^6^, Ruizhi Wang^3^, Kui Liu^7^, Xiaojuan Tang^8^, Qi Li^9^, Ran Xu^1,^**^*^**

^1^Department of Urology, The Second Xiangya Hospital of Central South University, Changsha, 410011, China;

^2^College of Traditional Chinese Medicine, Guangzhou University of Chinese Medicine, Guangzhou 510000, China;

^3^Department of Urology, The First Affiliated Hospital of Zhengzhou University, Henan 450052, China;

^4^Department of Pediatric Urology, Guangzhou Women and Children's Medical Center, Guangzhou Medical University, Guangzhou 510623, PR China.

^5^Department of Oral and Maxillofacial-Head and Neck Oncology, Shanghai Ninth People’s Hospital, Shanghai Jiao Tong University School of Medicine, Shanghai, China;

^6^Department of Oncology, The First Affiliated Hospital of Nanjing Medical University, Nanjing, China;

^7^Department of Pediatric Surgery, The First Affiliated Hospital of Henan University of Science and Technology, Luoyang, 471000, China;

^8^Department of Plastic and Reconstructive Surgery, the First Affiliated Hospital of Zhengzhou University, Zhengzhou, Henan, 450052, China;

^9^Department of Pediatric Surgery, The First Affiliated Hospital of Zhengzhou University, Henan 450052, China.

***Correspondence**

Department of Urology, The Second Xiangya Hospital of Central South University, Changsha, 410011, China; Ran Xu (xuran@csu.edu.cn)

**Supplementary Figures**

Fig S1. Cellular atlas of BLCA cancer tissues, and validation of BLCA molecular subtypes in bulk cohorts, and the heatmap of different subtypes. ..................................................................................................................................page 3

**Supplementary Tables**

Table S1. Summary of datasets in this study.............................................................................page 4

Table S2. Specific pathways of each BCIS subgroup. .............................................................page 5

Table S3. Signature genes of each subgroup for NTP analysis...............................................page 23

Table S4. Clinical data summary and statistical analysis of four subgroups in TCGA BLCA cohort.......................................................................................................................................page 73

Table S5. Cell death signaling pathways.................................................................................page 74


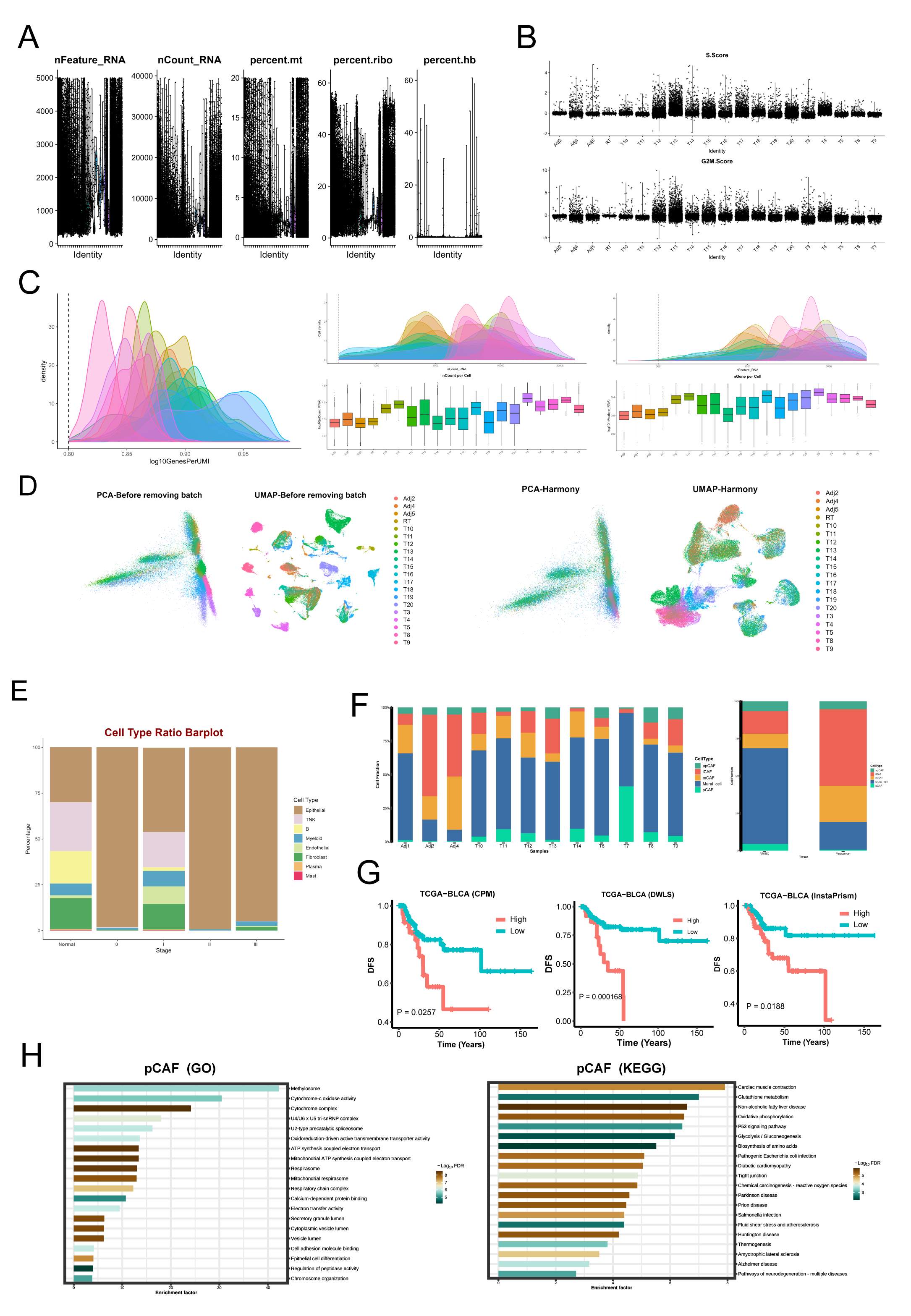


**Fig S1. Cellular atlas of BLCA cancer tissues, and validation of BLCA molecular subtypes in bulk cohorts, and the heatmap of different subtypes.** (**A**) The volcano plot showing high variable genes. (**B**) PCA showed significant separation of BLCA cells from different individuals. (**C**) PCA identified the top 50 PCs at P < 0.05. (**D**) UMAP expression plots of epithelial-cell subset markers, including EPCAM, KRT8, and KRT18. (**E**) UMAP plot of 6033 high-quality normal cells showing cell clusters, color-coded by the clusters. (**F**) UMAP plot of normal cells showing cell types, color-coded by the associated cell types. (**G**) Heatmap showing inferred CNVs for epithelial cells from 7 BLCA patients. The top-down view (BC1 to BC7). Red: amplification; blue: deletion. (**H**) The verification of OS among four subtypes in GEO cohorts, including GSE48276, GSE48075, GSE31684, and Meta Chort (GSE70691, GSE48276, GSE48075, GSE31684). (**I**) Heatmap showing 22 immune cell infiltration of four subtypes by Cibersort algorithm.


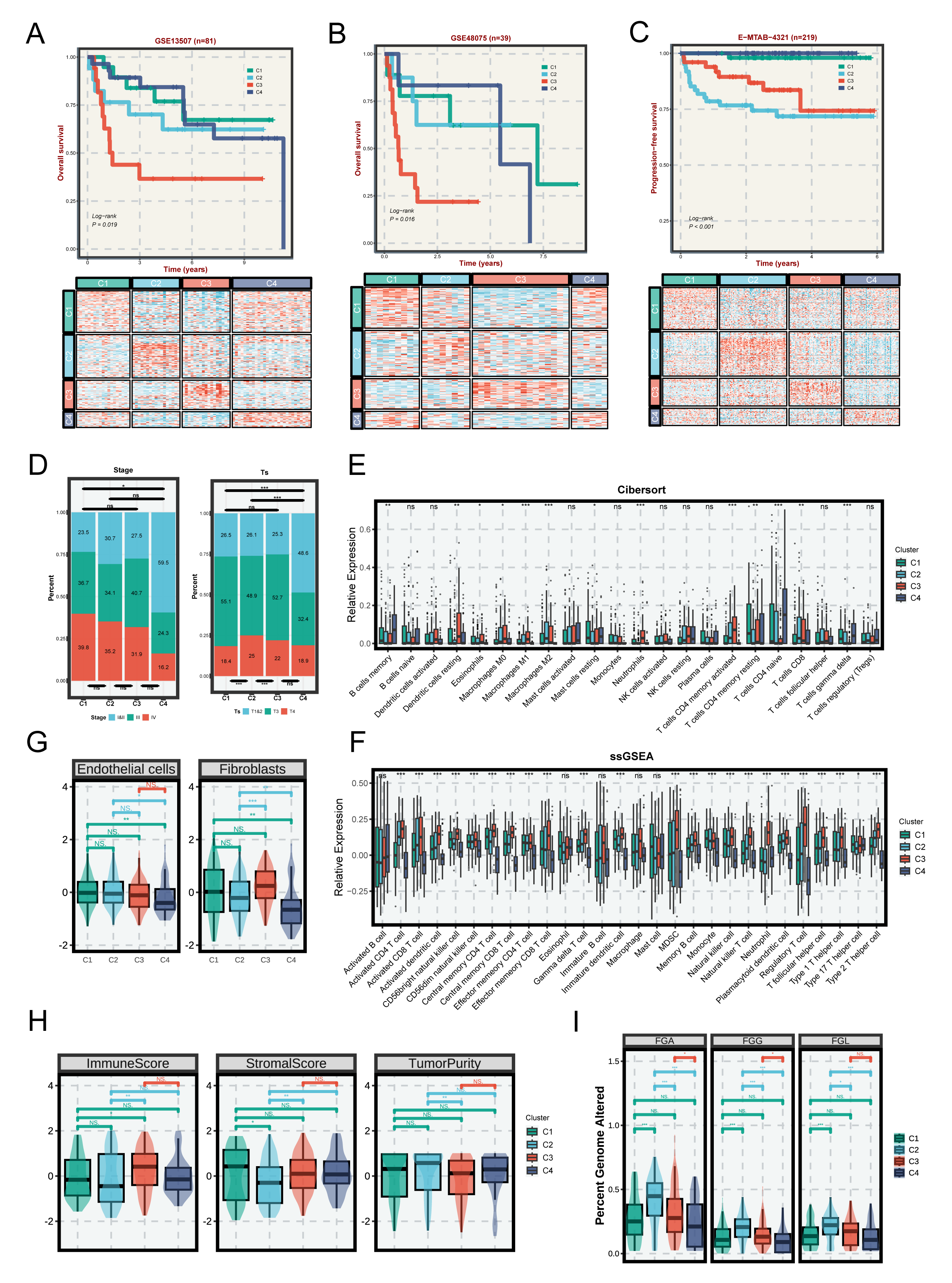


| **Table S1. Summary of datasets in this study** | | | | | | | | |
| --- | --- | --- | --- | --- | --- | --- | --- | --- |
| **Datasets** | **Platform** | **Number of  samples** | **Technology** | **PMID** | **Survival** | **Survival samples** | **Gene numbers** | **Source** |
| GSE135337 | Illumina NovaSeq 6000 | 8 | scRNA-seq | 34480339 | No |  | 21489 | [https://www.ncbi.nlm.nih.gov/geo/](https://www.ncbi.nlm.nih.gov/geo/) |
| TCGA_BLCA | Illumina | 418 | RNA-seq |  | Yes | 418 | 60484 | https://xena.ucsc.edu/ |
| E-MTAB-4321 | Illumina HiSeq 2000 | 460 | RNA-seq | 27321955 | Yes | 460 | 38272 | https://www.ebi.ac.uk/biostudies/arrayexpress |
| GSE13507 | Illumina human-6 v2.0 expression beadchip | 165 | Microarray | 20421545 | Yes | 165 | 24741 | [https://www.ncbi.nlm.nih.gov/geo/](https://www.ncbi.nlm.nih.gov/geo/) |
| GSE70691 | Illumina HumanHT-12 WG-DASL V4.0 R2 expression beadchip | 49 | Microarray | 26343003 | Yes | 49 | 21040 | [https://www.ncbi.nlm.nih.gov/geo/](https://www.ncbi.nlm.nih.gov/geo/) |
| GSE31684 | Affymetrix Human Genome U133 Plus 2.0 Array | 93 | Microarray | 24486590 | Yes | 93 | 23370 | [https://www.ncbi.nlm.nih.gov/geo/](https://www.ncbi.nlm.nih.gov/geo/) |
| GSE48075 | Illumina HumanHT-12 V3.0 expression beadchip | 73 | Microarray | 32546765 | Yes | 73 | 25058 | [https://www.ncbi.nlm.nih.gov/geo/](https://www.ncbi.nlm.nih.gov/geo/) |
| GSE48276 | Illumina HumanHT-12 WG-DASL V4.0 R2 expression beadchip | 73 | Microarray | 24525232 | Yes | 73 | 20883 | [https://www.ncbi.nlm.nih.gov/geo/](https://www.ncbi.nlm.nih.gov/geo/) |

Table S1: Summary of datasets in this study.

| **Table S2. Specific pathways of each BCIS subgroup** | | |
| --- | --- | --- |
| **cluster** | **path** | |
| BCIS1 | gocc cytosolic large ribosomal subunit | |
| BCIS1 | kegg ribosome |  |
| BCIS1 | gocc polysomal ribosome | |
| BCIS1 | gocc cytosolic ribosome | |
| BCIS1 | gocc cytosolic small ribosomal subunit | |
| BCIS1 | gobp cytoplasmic translation | |
| BCIS1 | gomf structural constituent of ribosome | |
| BCIS1 | gocc polysome |  |
| BCIS1 | gocc large ribosomal subunit | |
| BCIS1 | gocc ribosomal subunit | |
| BCIS1 | gocc small ribosomal subunit | |
| BCIS1 | gomf rrna binding | |
| BCIS1 | gobp ribosomal small subunit biogenesis | |
| BCIS1 | gobp ribosomal large subunit biogenesis | |
| BCIS1 | gobp ribosome assembly | |
| BCIS1 | gobp positive regulation of signal transduction by p53 class mediator | |
| BCIS1 | gobp translational elongation | |
| BCIS1 | gobp regulation of ubiquitin protein transferase activity | |
| BCIS1 | gobp regulation of signal transduction by p53 class mediator | |
| BCIS1 | gobp proteoglycan metabolic process | |
| BCIS1 | kegg abc transporters | |
| BCIS1 | gobp negative regulation of protein modification by small protein conjugation or removal | |
| BCIS1 | gobp regulation of intrinsic apoptotic signaling pathway in response to dna damage | |
| BCIS2 | gomf atp dependent protein folding chaperone | |
| BCIS2 | gobp positive regulation of telomere maintenance via telomere lengthening | |
| BCIS2 | gocc precatalytic spliceosome | |
| BCIS2 | gobp telomere maintenance via telomere lengthening | |
| BCIS2 | gobp rna templated dna biosynthetic process | |
| BCIS2 | gobp regulation of telomere maintenance via telomere lengthening | |
| BCIS2 | gobp mitochondrial gene expression | |
| BCIS2 | gobp mitochondrial translation | |
| BCIS2 | gobp positive regulation of dna biosynthetic process | |
| BCIS2 | gocc spliceosomal snrnp complex | |
| BCIS2 | hallmark myc targets v1 | |
| BCIS2 | gocc chaperone complex | |
| BCIS2 | gocc u2 type catalytic step 2 spliceosome | |
| BCIS2 | gomf protein folding chaperone | |
| BCIS2 | gocc sm like protein family complex | |
| BCIS2 | hallmark oxidative phosphorylation | |
| BCIS2 | gocc endopeptidase complex | |
| BCIS2 | gobp chaperone mediated protein folding | |
| BCIS2 | gobp positive regulation of telomere maintenance | |
| BCIS2 | gobp protein localization to chromosome | |
| BCIS2 | kegg pyruvate metabolism | |
| BCIS2 | gobp telomere maintenance | |
| BCIS2 | gobp telomere organization | |
| BCIS2 | kegg spliceosome | |
| BCIS2 | gobp nadh dehydrogenase complex assembly | |
| BCIS2 | gocc peptidase complex | |
| BCIS2 | gobp regulation of telomere maintenance | |
| BCIS2 | gobp positive regulation of chromosome organization | |
| BCIS2 | gocc u2 type spliceosomal complex | |
| BCIS2 | gocc methyltransferase complex | |
| BCIS2 | hallmark e2f targets | |
| BCIS2 | gocc catalytic step 2 spliceosome | |
| BCIS2 | gobp atp synthesis coupled electron transport | |
| BCIS2 | gocc oxidoreductase complex | |
| BCIS2 | gocc chromosome telomeric region | |
| BCIS2 | gomf unfolded protein binding | |
| BCIS2 | gomf single stranded dna binding | |
| BCIS2 | gobp respiratory electron transport chain | |
| BCIS2 | gocc pigment granule | |
| BCIS2 | gobp mitochondrial electron transport nadh to ubiquinone | |
| BCIS2 | gobp mitochondrial respiratory chain complex assembly | |
| BCIS2 | gocc inner mitochondrial membrane protein complex | |
| BCIS2 | gobp oxidative phosphorylation | |
| BCIS2 | kegg parkinsons disease | |
| BCIS2 | gomf molecular carrier activity | |
| BCIS2 | gocc spliceosomal complex | |
| BCIS2 | gocc respirasome | |
| BCIS2 | gobp electron transport chain | |
| BCIS2 | gomf oxidoreduction driven active transmembrane transporter activity | |
| BCIS2 | gobp positive regulation of protein localization to nucleus | |
| BCIS2 | kegg propanoate metabolism | |
| BCIS2 | kegg huntingtons disease | |
| BCIS2 | gobp aerobic respiration | |
| BCIS2 | gobp recombinational repair | |
| BCIS2 | gobp arp2 3 complex mediated actin nucleation | |
| BCIS2 | gocc organelle envelope lumen | |
| BCIS2 | kegg valine leucine and isoleucine degradation | |
| BCIS2 | gomf electron transfer activity | |
| BCIS2 | gobp regulation of dna biosynthetic process | |
| BCIS2 | gomf isomerase activity | |
| BCIS2 | gobp rna localization | |
| BCIS2 | gobp mitochondrial electron transport cytochrome c to oxygen | |
| BCIS2 | gobp mitotic sister chromatid separation | |
| BCIS2 | gomf oxidoreductase activity acting on nad p h | |
| BCIS2 | gomf oxidoreductase activity acting on nad p h quinone or similar compound as acceptor | |
| BCIS2 | gobp mitochondrial transmembrane transport | |
| BCIS2 | gocc cytochrome complex | |
| BCIS2 | gomf metal cluster binding | |
| BCIS2 | gobp actin nucleation | |
| BCIS2 | gobp dna biosynthetic process | |
| BCIS2 | gobp protein export from nucleus | |
| BCIS2 | kegg pathogenic escherichia coli infection | |
| BCIS2 | gobp rna export from nucleus | |
| BCIS2 | gobp metaphase plate congression | |
| BCIS2 | gobp mitotic metaphase plate congression | |
| BCIS2 | kegg alzheimers disease | |
| BCIS2 | gocc clathrin adaptor complex | |
| BCIS2 | gobp spliceosomal complex assembly | |
| BCIS2 | gobp negative regulation of chromosome organization | |
| BCIS2 | gocc respiratory chain complex iv | |
| BCIS2 | gobp dna conformation change | |
| BCIS2 | gocc site of dna damage | |
| BCIS2 | gocc nuclear pore | |
| BCIS2 | gobp chromosome separation | |
| BCIS2 | gobp anaphase promoting complex dependent catabolic process | |
| BCIS2 | gobp regulation of mitotic sister chromatid segregation | |
| BCIS2 | gobp cell cell recognition | |
| BCIS2 | gocc anaphase promoting complex | |
| BCIS2 | hallmark pi3k akt mtor signaling | |
| BCIS2 | hallmark g2m checkpoint | |
| BCIS2 | gobp protein n linked glycosylation | |
| BCIS2 | gobp pyruvate metabolic process | |
| BCIS2 | kegg n glycan biosynthesis | |
| BCIS2 | gobp cytokinetic process | |
| BCIS2 | gobp cellular response to heat | |
| BCIS2 | gomf ribosome binding | |
| BCIS2 | gobp regulation of mrna splicing via spliceosome | |
| BCIS2 | gobp protein modification by small protein removal | |
| BCIS2 | gobp vesicle budding from membrane | |
| BCIS2 | gocc condensed chromosome centromeric region | |
| BCIS2 | gobp regulation of sister chromatid segregation | |
| BCIS2 | gobp quinone metabolic process | |
| BCIS2 | gobp microtubule cytoskeleton organization involved in mitosis | |
| BCIS2 | hallmark fatty acid metabolism | |
| BCIS2 | gobp nuclear export | |
| BCIS2 | gobp ubiquitin dependent erad pathway | |
| BCIS2 | gobp nuclear membrane organization | |
| BCIS2 | gobp regulation of chromosome segregation | |
| BCIS2 | kegg butanoate metabolism | |
| BCIS2 | gobp mitotic spindle organization | |
| BCIS2 | gocc ficolin 1 rich granule lumen | |
| BCIS2 | gobp chromosome localization | |
| BCIS2 | gomf double stranded rna binding | |
| BCIS2 | gomf oxidoreductase activity acting on ch oh group of donors | |
| BCIS2 | gomf oxidoreductase activity acting on the ch ch group of donors | |
| BCIS2 | gocc clathrin vesicle coat | |
| BCIS2 | gobp fatty acid beta oxidation | |
| BCIS2 | gobp protein targeting to mitochondrion | |
| BCIS2 | gobp midbrain development | |
| BCIS2 | gocc cullin ring ubiquitin ligase complex | |
| BCIS2 | gobp regulation of stem cell population maintenance | |
| BCIS2 | gocc vesicle coat | |
| BCIS2 | gobp mitotic cytokinesis | |
| BCIS2 | kegg glycolysis gluconeogenesis | |
| BCIS2 | gobp mitotic spindle assembly | |
| BCIS2 | gobp nucleotide excision repair | |
| BCIS2 | gocc spindle microtubule | |
| BCIS2 | gobp regulation of mitotic nuclear division | |
| BCIS2 | gobp vitamin metabolic process | |
| BCIS2 | gobp positive regulation of cell cycle phase transition | |
| BCIS2 | gobp protein k11 linked ubiquitination | |
| BCIS2 | gobp cytoskeleton dependent cytokinesis | |
| BCIS2 | gocc clathrin coat | |
| BCIS2 | gobp positive regulation of double strand break repair | |
| BCIS2 | gomf translation initiation factor activity | |
| BCIS2 | kegg fatty acid metabolism | |
| BCIS2 | gomf ubiquitin like protein peptidase activity | |
| BCIS2 | gobp protein localization to mitochondrion | |
| BCIS2 | hallmark mtorc1 signaling | |
| BCIS2 | gobp porphyrin containing compound metabolic process | |
| BCIS2 | gocc golgi associated vesicle membrane | |
| BCIS2 | gobp establishment of rna localization | |
| BCIS2 | gobp regulation of protein dephosphorylation | |
| BCIS2 | hallmark adipogenesis | |
| BCIS2 | gocc ap type membrane coat adaptor complex | |
| BCIS2 | gobp regulation of nuclear division | |
| BCIS2 | gobp positive regulation of dna repair | |
| BCIS2 | kegg pyrimidine metabolism | |
| BCIS2 | gobp neural nucleus development | |
| BCIS2 | gobp regulation of mrna processing | |
| BCIS2 | gocc endoplasmic reticulum golgi intermediate compartment | |
| BCIS2 | gocc ficolin 1 rich granule | |
| BCIS2 | gobp spindle assembly | |
| BCIS2 | gomf structural constituent of cytoskeleton | |
| BCIS2 | gomf heat shock protein binding | |
| BCIS2 | gobp activation of cysteine type endopeptidase activity involved in apoptotic process | |
| BCIS2 | gobp monosaccharide biosynthetic process | |
| BCIS2 | gobp erad pathway | |
| BCIS2 | kegg oocyte meiosis | |
| BCIS2 | gobp nuclear envelope organization | |
| BCIS2 | gocc microtubule associated complex | |
| BCIS2 | gomf ribonucleoprotein complex binding | |
| BCIS2 | gomf chaperone binding | |
| BCIS2 | gobp regulation of generation of precursor metabolites and energy | |
| BCIS2 | hallmark protein secretion | |
| BCIS2 | gobp fatty acid catabolic process | |
| BCIS2 | gobp toxin transport | |
| BCIS2 | gobp monocarboxylic acid catabolic process | |
| BCIS2 | gobp regulation of double strand break repair | |
| BCIS2 | gobp post translational protein modification | |
| BCIS2 | gobp negative regulation of protein polymerization | |
| BCIS2 | gobp tetrapyrrole metabolic process | |
| BCIS2 | gocc histone deacetylase complex | |
| BCIS2 | gobp nucleoside bisphosphate biosynthetic process | |
| BCIS2 | kegg progesterone mediated oocyte maturation | |
| BCIS2 | gocc nuclear ubiquitin ligase complex | |
| BCIS2 | gobp dicarboxylic acid metabolic process | |
| BCIS2 | gobp regulation of postsynaptic membrane neurotransmitter receptor levels | |
| BCIS2 | gobp adp metabolic process | |
| BCIS2 | gobp translational initiation | |
| BCIS2 | gocc golgi associated vesicle | |
| BCIS2 | gobp cellular pigmentation | |
| BCIS2 | gobp negative regulation of protein catabolic process | |
| BCIS2 | gobp carbohydrate catabolic process | |
| BCIS2 | gobp protein transmembrane transport | |
| BCIS2 | gobp response to heat | |
| BCIS2 | gobp regulation of cell division | |
| BCIS2 | gobp rna modification | |
| BCIS2 | gocc membrane coat | |
| BCIS2 | gocc nuclear periphery | |
| BCIS2 | gobp pigment granule organization | |
| BCIS2 | gocc trans golgi network transport vesicle | |
| BCIS2 | gobp cytokinesis | |
| BCIS2 | gobp regulation of cytokinesis | |
| BCIS2 | gomf flavin adenine dinucleotide binding | |
| BCIS2 | gobp single fertilization | |
| BCIS2 | gobp regulation of protein localization to nucleus | |
| BCIS2 | gocc extrinsic component of organelle membrane | |
| BCIS2 | gobp regulation of dna replication | |
| BCIS2 | gobp mitotic cell cycle checkpoint signaling | |
| BCIS2 | gobp positive regulation of cell division | |
| BCIS2 | kegg cell cycle | |
| BCIS2 | gobp zymogen activation | |
| BCIS2 | gobp regulation of meiotic cell cycle | |
| BCIS2 | gobp positive regulation of cysteine type endopeptidase activity | |
| BCIS2 | gobp pigment biosynthetic process | |
| BCIS2 | gomf proton transmembrane transporter activity | |
| BCIS2 | gobp meiotic chromosome segregation | |
| BCIS2 | gobp mitochondrial transport | |
| BCIS2 | gocc microbody membrane | |
| BCIS2 | gobp mitochondrial membrane organization | |
| BCIS2 | gobp protein containing complex localization | |
| BCIS2 | gobp dna templated transcription elongation | |
| BCIS2 | hallmark unfolded protein response | |
| BCIS2 | gobp regulation of nucleocytoplasmic transport | |
| BCIS2 | gobp synaptic vesicle cytoskeletal transport | |
| BCIS2 | gobp cell cycle checkpoint signaling | |
| BCIS2 | gobp nucleotide phosphorylation | |
| BCIS2 | gocc mitotic spindle | |
| BCIS2 | gobp trna processing | |
| BCIS2 | gobp mitochondrial outer membrane permeabilization | |
| BCIS2 | gomf oxidoreductase activity acting on the aldehyde or oxo group of donors nad or nadp as acceptor | |
| BCIS2 | gobp maintenance of protein location in cell | |
| BCIS2 | gomf aldehyde dehydrogenase nad p plus activity | |
| BCIS2 | gomf thiolester hydrolase activity | |
| BCIS2 | gobp response to leukemia inhibitory factor | |
| BCIS2 | gobp proton transmembrane transport | |
| BCIS2 | gocc axon cytoplasm | |
| BCIS2 | gobp regulation of dna binding | |
| BCIS2 | gobp positive regulation of peptidase activity | |
| BCIS2 | gobp regulation of centrosome cycle | |
| BCIS2 | kegg ubiquitin mediated proteolysis | |
| BCIS2 | gobp histone h4 acetylation | |
| BCIS2 | gobp organophosphate catabolic process | |
| BCIS2 | hallmark glycolysis | |
| BCIS2 | gobp steroid hormone mediated signaling pathway | |
| BCIS2 | gobp golgi to plasma membrane transport | |
| BCIS2 | gocc rough endoplasmic reticulum | |
| BCIS2 | kegg purine metabolism | |
| BCIS2 | gobp nucleoside diphosphate metabolic process | |
| BCIS2 | gobp axo dendritic transport | |
| BCIS2 | gocc fibrillar center | |
| BCIS2 | gobp pigment metabolic process | |
| BCIS2 | gocc pml body | |
| BCIS2 | gobp trna metabolic process | |
| BCIS2 | gobp nucleus organization | |
| BCIS2 | gobp positive regulation of translation | |
| BCIS2 | gobp vesicle transport along microtubule | |
| BCIS2 | gobp centrosome duplication | |
| BCIS2 | gobp cytosolic transport | |
| BCIS2 | gobp maintenance of protein location | |
| BCIS2 | gobp lipid oxidation | |
| BCIS2 | gomf carbon oxygen lyase activity | |
| BCIS2 | gobp positive regulation of binding | |
| BCIS2 | gobp axonal transport | |
| BCIS2 | gomf oxidoreductase activity acting on the aldehyde or oxo group of donors | |
| BCIS2 | gobp purine nucleoside monophosphate metabolic process | |
| BCIS2 | gocc sarcoplasmic reticulum | |
| BCIS2 | gocc organelle membrane contact site | |
| BCIS2 | gobp organelle transport along microtubule | |
| BCIS2 | gobp dna dealkylation | |
| BCIS2 | gobp amino acid biosynthetic process | |
| BCIS2 | gobp regulation of multicellular organism growth | |
| BCIS2 | gobp cellular aldehyde metabolic process | |
| BCIS2 | gobp nucleoside monophosphate metabolic process | |
| BCIS2 | gobp glucose metabolic process | |
| BCIS2 | gobp fatty acid derivative metabolic process | |
| BCIS2 | gobp peptidyl lysine acetylation | |
| BCIS2 | gobp pigmentation | |
| BCIS2 | gobp nucleoside bisphosphate metabolic process | |
| BCIS2 | gobp transport along microtubule | |
| BCIS2 | gobp negative regulation of transmembrane transport | |
| BCIS2 | gomf acetyltransferase activity | |
| BCIS2 | gobp anterograde axonal transport | |
| BCIS2 | gobp mitochondrial outer membrane permeabilization involved in programmed cell death | |
| BCIS2 | gobp regulation of mitochondrial membrane permeability involved in apoptotic process | |
| BCIS2 | gobp negative regulation of dna metabolic process | |
| BCIS2 | gobp positive regulation of mitochondrion organization | |
| BCIS2 | gobp centriole assembly | |
| BCIS3 | hallmark tnfa signaling via nfkb | |
| BCIS3 | gobp integrated stress response signaling | |
| BCIS3 | hallmark hypoxia | |
| BCIS3 | gobp skeletal muscle cell differentiation | |
| BCIS3 | gobp mirna metabolic process | |
| BCIS3 | gobp positive regulation of fat cell differentiation | |
| BCIS3 | gobp embryonic placenta development | |
| BCIS3 | gobp regulation of epithelial cell differentiation | |
| BCIS3 | gobp osteoclast differentiation | |
| BCIS3 | gobp cellular response to starvation | |
| BCIS3 | gobp negative regulation of leukocyte proliferation | |
| BCIS3 | gobp response to camp | |
| BCIS3 | gobp mesonephros development | |
| BCIS3 | hallmark inflammatory response | |
| BCIS3 | gobp myoblast differentiation | |
| BCIS3 | gobp regulation of fat cell differentiation | |
| BCIS3 | gobp negative regulation of developmental growth | |
| BCIS3 | gobp positive regulation of peptide secretion | |
| BCIS3 | gobp cellular response to osmotic stress | |
| BCIS3 | gobp keratinocyte differentiation | |
| BCIS3 | gobp regulation of osteoblast differentiation | |
| BCIS3 | gobp regulation of nervous system process | |
| BCIS3 | gobp skeletal muscle organ development | |
| BCIS3 | gobp kidney epithelium development | |
| BCIS3 | gobp response to organophosphorus | |
| BCIS3 | gobp negative regulation of leukocyte cell cell adhesion | |
| BCIS3 | hallmark il2 stat5 signaling | |
| BCIS3 | gobp tissue regeneration | |
| BCIS3 | gobp formation of primary germ layer | |
| BCIS3 | gobp regulation of cd4 positive alpha beta t cell activation | |
| BCIS3 | kegg arrhythmogenic right ventricular cardiomyopathy arvc | |
| BCIS3 | gocc actin filament | |
| BCIS3 | hallmark tgf beta signaling | |
| BCIS3 | gobp cellular response to carbohydrate stimulus | |
| BCIS3 | gobp positive regulation of vasculature development | |
| BCIS3 | gobp positive regulation of response to wounding | |
| BCIS3 | gobp positive regulation of hormone secretion | |
| BCIS3 | gobp intrinsic apoptotic signaling pathway in response to endoplasmic reticulum stress | |
| BCIS3 | gobp nephron development | |
| BCIS3 | gobp endoplasmic reticulum unfolded protein response | |
| BCIS3 | gobp nephron epithelium development | |
| BCIS3 | gobp response to osmotic stress | |
| BCIS3 | gobp negative regulation of lymphocyte activation | |
| BCIS3 | kegg leukocyte transendothelial migration | |
| BCIS3 | gobp regulation of axonogenesis | |
| BCIS3 | hallmark p53 pathway | |
| BCIS3 | gobp er nucleus signaling pathway | |
| BCIS3 | hallmark apoptosis | |
| BCIS3 | gobp regulation of myoblast differentiation | |
| BCIS3 | hallmark cholesterol homeostasis | |
| BCIS3 | gocc recycling endosome membrane | |
| BCIS3 | gobp response to bmp | |
| BCIS3 | gobp positive regulation of stress activated protein kinase signaling cascade | |
| BCIS3 | gobp negative regulation of vasculature development | |
| BCIS3 | gobp sprouting angiogenesis | |
| BCIS3 | gocc tight junction | |
| BCIS3 | kegg cell adhesion molecules cams | |
| BCIS3 | gobp positive regulation of biomineral tissue development | |
| BCIS3 | gocc apical junction complex | |
| BCIS3 | hallmark uv response up | |
| BCIS3 | gobp positive regulation of protein secretion | |
| BCIS3 | gobp actin filament based movement | |
| BCIS3 | hallmark myogenesis | |
| BCIS3 | gobp regulation of wound healing | |
| BCIS3 | hallmark il6 jak stat3 signaling | |
| BCIS3 | gobp associative learning | |
| BCIS3 | gobp negative regulation of secretion | |
| BCIS3 | gobp amino acid transport | |
| BCIS3 | gobp placenta development | |
| BCIS3 | gobp cd4 positive alpha beta t cell activation | |
| BCIS3 | gobp regulation of stress activated protein kinase signaling cascade | |
| BCIS3 | gobp notch signaling pathway | |
| BCIS3 | gobp response to purine containing compound | |
| BCIS3 | gobp pathway restricted smad protein phosphorylation | |
| BCIS3 | gobp columnar cuboidal epithelial cell differentiation | |
| BCIS3 | gocc lateral plasma membrane | |
| BCIS3 | hallmark epithelial mesenchymal transition | |
| BCIS3 | gobp myotube differentiation | |
| BCIS3 | gobp regulation of insulin secretion | |
| BCIS3 | gobp intracellular glucose homeostasis | |
| BCIS3 | gobp negative regulation of extrinsic apoptotic signaling pathway | |
| BCIS3 | gomf kinase activator activity | |
| BCIS3 | gobp regulation of myeloid leukocyte differentiation | |
| BCIS3 | gobp regulation of cell shape | |
| BCIS3 | kegg focal adhesion | |
| BCIS3 | gobp regulation of response to wounding | |
| BCIS3 | gobp regulation of peptide transport | |
| BCIS3 | gocc early endosome membrane | |
| BCIS3 | gobp regulation of epithelial cell apoptotic process | |
| BCIS3 | kegg hypertrophic cardiomyopathy hcm | |
| BCIS3 | gobp response to interleukin 1 | |
| BCIS3 | gobp cyclic nucleotide mediated signaling | |
| BCIS3 | gobp actin mediated cell contraction | |
| BCIS3 | kegg axon guidance | |
| BCIS3 | gobp negative regulation of mapk cascade | |
| BCIS3 | gomf cargo receptor activity | |
| BCIS3 | gobp negative regulation of immune effector process | |
| BCIS3 | kegg tight junction | |
| BCIS3 | gocc adherens junction | |
| BCIS3 | gobp cellular component assembly involved in morphogenesis | |
| BCIS3 | gobp positive regulation of t cell proliferation | |
| BCIS3 | gomf exogenous protein binding | |
| BCIS3 | gobp gland morphogenesis | |
| BCIS3 | gobp lipid storage | |
| BCIS3 | gobp regulation of steroid biosynthetic process | |
| BCIS3 | gobp insulin secretion | |
| BCIS3 | gobp regulation of alpha beta t cell activation | |
| BCIS3 | gobp positive regulation of epithelial cell migration | |
| BCIS3 | gobp smooth muscle cell proliferation | |
| BCIS3 | hallmark kras signaling up | |
| BCIS3 | gobp neural tube development | |
| BCIS3 | gobp positive regulation of endothelial cell migration | |
| BCIS3 | gobp regulation of b cell activation | |
| BCIS3 | gocc schaffer collateral ca1 synapse | |
| BCIS3 | hallmark apical junction | |
| BCIS3 | gobp development of primary female sexual characteristics | |
| BCIS3 | gobp regulation of biomineral tissue development | |
| BCIS3 | gomf growth factor receptor binding | |
| BCIS3 | gobp positive regulation of developmental growth | |
| BCIS3 | gobp regeneration | |
| BCIS3 | gobp blood vessel endothelial cell migration | |
| BCIS3 | gobp regulation of ras protein signal transduction | |
| BCIS3 | gobp morphogenesis of a polarized epithelium | |
| BCIS3 | gomf smad binding | |
| BCIS3 | gobp jnk cascade | |
| BCIS3 | gobp negative regulation of protein serine threonine kinase activity | |
| BCIS3 | gobp muscle cell development | |
| BCIS3 | gobp actin cytoskeleton reorganization | |
| BCIS3 | gocc neuron projection terminus | |
| BCIS3 | gobp cell cell junction assembly | |
| BCIS3 | hallmark heme metabolism | |
| BCIS3 | gobp actin filament bundle organization | |
| BCIS3 | kegg b cell receptor signaling pathway | |
| BCIS3 | gobp muscle cell apoptotic process | |
| BCIS3 | gobp positive regulation of transmembrane receptor protein serine threonine kinase signaling pathway | |
| BCIS4 | gocc nadh dehydrogenase complex | |
| BCIS4 | gomf nad p h dehydrogenase quinone activity | |
| BCIS4 | gobp proton motive force driven atp synthesis | |
| BCIS4 | gomf oxidoreductase activity acting on peroxide as acceptor | |
| BCIS4 | gomf antioxidant activity | |
| BCIS4 | gocc endoplasmic reticulum protein containing complex | |
| BCIS4 | gobp atp biosynthetic process | |
| BCIS4 | hallmark reactive oxygen species pathway | |
| BCIS4 | gomf calcium dependent protein binding | |
| BCIS4 | gocc phagocytic vesicle membrane | |
| BCIS4 | gobp cellular oxidant detoxification | |
| BCIS4 | gobp nucleoside triphosphate biosynthetic process | |
| BCIS4 | kegg oxidative phosphorylation | |
| BCIS4 | gocc azurophil granule membrane | |
| BCIS4 | gobp vacuolar acidification | |
| BCIS4 | gobp positive regulation of interleukin 8 production | |
| BCIS4 | gocc proton transporting v type atpase complex | |
| BCIS4 | gocc azurophil granule | |
| BCIS4 | gomf serine type endopeptidase inhibitor activity | |
| BCIS4 | gobp regulation of mitochondrial membrane potential | |
| BCIS4 | gocc ficolin 1 rich granule membrane | |
| BCIS4 | gomf copper ion binding | |
| BCIS4 | gocc proton transporting two sector atpase complex | |
| BCIS4 | gomf pyrophosphate hydrolysis driven proton transmembrane transporter activity | |
| BCIS4 | gobp negative regulation of viral process | |
| BCIS4 | gocc tertiary granule membrane | |
| BCIS4 | gocc tertiary granule | |
| BCIS4 | gobp intracellular ph reduction | |
| BCIS4 | gomf endopeptidase regulator activity | |
| BCIS4 | gobp interleukin 8 production | |
| BCIS4 | gobp nucleoside metabolic process | |
| BCIS4 | gobp regulation of ph | |
| BCIS4 | gocc blood microparticle | |
| BCIS4 | gocc synaptic vesicle membrane | |
| BCIS4 | gobp neutrophil chemotaxis | |
| BCIS4 | gobp positive regulation of phagocytosis | |
| BCIS4 | hallmark complement | |
| BCIS4 | gocc endocytic vesicle membrane | |
| BCIS4 | gocc specific granule membrane | |
| BCIS4 | gobp sterol transport | |
| BCIS4 | gobp neutrophil migration | |
| BCIS4 | gomf hydrolase activity acting on carbon nitrogen but not peptide bonds in linear amides | |
| BCIS4 | gobp biological process involved in interaction with symbiont | |
| BCIS4 | gomf primary active transmembrane transporter activity | |
| BCIS4 | gomf channel regulator activity | |
| BCIS4 | gobp regulation of sterol transport | |
| BCIS4 | gobp sterol homeostasis | |
| BCIS4 | gobp positive regulation of lipid localization | |
| BCIS4 | gobp positive regulation of lipid transport | |
| BCIS4 | gobp regulation of oxidoreductase activity | |
| BCIS4 | gomf phospholipase activity | |
| BCIS4 | gocc 9plus0 non motile cilium | |
| BCIS4 | gobp regulation of lipid transport | |
| BCIS4 | gobp regulation of lipid localization | |
| BCIS4 | gobp endocrine process | |

Table S2: Specific pathways of each BCIS subgroup

| **Table S3. Signature genes of each subgroup for NTP analysis** | | | | | **Table S2. Signature genes of each subgroup for NTP analysis** | | |
| --- | --- | --- | --- | --- | --- | --- | --- |
| **Cluster Probe** | |  |  |  | |  |  |
| C1 | PTN |  |  |  | |  |  |
| C1 | UPK2 |  |  |  | |  |  |
| C1 | UPK3A |  |  |  | |  |  |
| C1 | PPP1CB |  |  |  | |  |  |
| C1 | SPTSSB |  |  |  | |  |  |
| C1 | SCNN1B |  |  |  | |  |  |
| C1 | GRHL3 |  |  |  | |  |  |
| C1 | EIF5A |  |  |  | |  |  |
| C1 | TFF2 |  |  |  | |  |  |
| C1 | CDKN2A |  |  |  | |  |  |
| C1 | CAMK2N1 |  |  |  | |  |  |
| C1 | BHMT |  |  |  | |  |  |
| C1 | RCN1 |  |  |  | |  |  |
| C1 | HNRNPH1 |  |  |  | |  |  |
| C1 | MRPS21 |  |  |  | |  |  |
| C1 | PADI3 |  |  |  | |  |  |
| C1 | TBX2 |  |  |  | |  |  |
| C1 | IGF2 |  |  |  | |  |  |
| C1 | SNX31 |  |  |  | |  |  |
| C1 | QSOX1 |  |  |  | |  |  |
| C1 | S100A14 |  |  |  | |  |  |
| C1 | KRT80 |  |  |  | |  |  |
| C1 | CTNNB1 |  |  |  | |  |  |
| C1 | KDELR2 |  |  |  | |  |  |
| C1 | GLO1 |  |  |  | |  |  |
| C1 | CALR |  |  |  | |  |  |
| C1 | S100A9 |  |  |  | |  |  |
| C1 | CKB |  |  |  | |  |  |
| C1 | EIF5B |  |  |  | |  |  |
| C1 | CDC42SE1 |  |  |  | |  |  |
| C1 | IL1RN |  |  |  | |  |  |
| C1 | SRM |  |  |  | |  |  |
| C1 | LGALS1 |  |  |  | |  |  |
| C1 | KSR2 |  |  |  | |  |  |
| C1 | RRBP1 |  |  |  | |  |  |
| C1 | NBL1 |  |  |  | |  |  |
| C1 | NME4 |  |  |  | |  |  |
| C1 | CTTN |  |  |  | |  |  |
| C1 | MAL2 |  |  |  | |  |  |
| C1 | GPX3 |  |  |  | |  |  |
| C1 | PLA2G2A |  |  |  | |  |  |
| C1 | CRCT1 |  |  |  | |  |  |
| C1 | AGRN |  |  |  | |  |  |
| C1 | CDH1 |  |  |  | |  |  |
| C1 | SLC25A1 |  |  |  | |  |  |
| C1 | MYCL |  |  |  | |  |  |
| C1 | TFF1 |  |  |  | |  |  |
| C1 | NRIP1 |  |  |  | |  |  |
| C1 | APOBEC3C |  |  |  | |  |  |
| C1 | PHF14 |  |  |  | |  |  |
| C1 | LY6E |  |  |  | |  |  |
| C1 | MAL |  |  |  | |  |  |
| C1 | SDC4 |  |  |  | |  |  |
| C1 | GSTM5 |  |  |  | |  |  |
| C1 | MRPL36 |  |  |  | |  |  |
| C1 | HMGA1 |  |  |  | |  |  |
| C1 | TIMM13 |  |  |  | |  |  |
| C1 | SET |  |  |  | |  |  |
| C1 | C1orf56 |  |  |  | |  |  |
| C1 | TRA2A |  |  |  | |  |  |
| C1 | TOP2B |  |  |  | |  |  |
| C1 | GOLPH3 |  |  |  | |  |  |
| C1 | SCOC |  |  |  | |  |  |
| C1 | ACTR2 |  |  |  | |  |  |
| C1 | UHMK1 |  |  |  | |  |  |
| C1 | SLC38A2 |  |  |  | |  |  |
| C1 | SDF4 |  |  |  | |  |  |
| C1 | POGK |  |  |  | |  |  |
| C1 | TAGLN2 |  |  |  | |  |  |
| C1 | TCEA1 |  |  |  | |  |  |
| C1 | SRSF11 |  |  |  | |  |  |
| C1 | CRIM1 |  |  |  | |  |  |
| C1 | IVL |  |  |  | |  |  |
| C1 | GJB6 |  |  |  | |  |  |
| C1 | ARPC4 |  |  |  | |  |  |
| C1 | MED10 |  |  |  | |  |  |
| C1 | CMTM6 |  |  |  | |  |  |
| C1 | PGD |  |  |  | |  |  |
| C1 | CHMP4B |  |  |  | |  |  |
| C1 | TTC14 |  |  |  | |  |  |
| C1 | ANO10 |  |  |  | |  |  |
| C1 | MAN1A1 |  |  |  | |  |  |
| C1 | HEBP2 |  |  |  | |  |  |
| C1 | UPF2 |  |  |  | |  |  |
| C1 | PNCK |  |  |  | |  |  |
| C1 | NDUFA4L2 |  |  |  | |  |  |
| C1 | TMEM54 |  |  |  | |  |  |
| C1 | TOX3 |  |  |  | |  |  |
| C1 | DEK |  |  |  | |  |  |
| C1 | IDH1 |  |  |  | |  |  |
| C1 | ELP5 |  |  |  | |  |  |
| C1 | SMS |  |  |  | |  |  |
| C1 | BTF3L4 |  |  |  | |  |  |
| C1 | HIGD1A |  |  |  | |  |  |
| C1 | SMCO4 |  |  |  | |  |  |
| C1 | OS9 |  |  |  | |  |  |
| C1 | SLC26A2 |  |  |  | |  |  |
| C1 | TMPRSS2 |  |  |  | |  |  |
| C1 | KRT20 |  |  |  | |  |  |
| C1 | COX17 |  |  |  | |  |  |
| C1 | SPOPL |  |  |  | |  |  |
| C1 | PODXL2 |  |  |  | |  |  |
| C1 | TMEM167A |  |  |  | |  |  |
| C1 | TBL1XR1 |  |  |  | |  |  |
| C1 | EPHX3 |  |  |  | |  |  |
| C1 | YWHAE |  |  |  | |  |  |
| C1 | SYNC |  |  |  | |  |  |
| C1 | ZNF217 |  |  |  | |  |  |
| C1 | HDGF |  |  |  | |  |  |
| C1 | DYNC1I2 |  |  |  | |  |  |
| C1 | CD99 |  |  |  | |  |  |
| C1 | TCN1 |  |  |  | |  |  |
| C1 | CMPK1 |  |  |  | |  |  |
| C1 | PRPF4B |  |  |  | |  |  |
| C1 | SLC6A8 |  |  |  | |  |  |
| C1 | RCC2 |  |  |  | |  |  |
| C1 | CLCA2 |  |  |  | |  |  |
| C1 | PDIA4 |  |  |  | |  |  |
| C1 | UPK1A |  |  |  | |  |  |
| C1 | POLR2K |  |  |  | |  |  |
| C1 | SMYD3 |  |  |  | |  |  |
| C1 | ZNF117 |  |  |  | |  |  |
| C1 | CYP4F22 |  |  |  | |  |  |
| C1 | NUCKS1 |  |  |  | |  |  |
| C1 | YWHAH |  |  |  | |  |  |
| C1 | RANBP1 |  |  |  | |  |  |
| C1 | BRD7 |  |  |  | |  |  |
| C1 | RNMT |  |  |  | |  |  |
| C1 | TRAM1 |  |  |  | |  |  |
| C1 | SPINT1 |  |  |  | |  |  |
| C1 | TRAK1 |  |  |  | |  |  |
| C1 | ATOX1 |  |  |  | |  |  |
| C1 | HMGCS2 |  |  |  | |  |  |
| C1 | MARCKS |  |  |  | |  |  |
| C1 | HMGB3 |  |  |  | |  |  |
| C1 | ACBD3 |  |  |  | |  |  |
| C1 | CTBP1 |  |  |  | |  |  |
| C1 | SRSF6 |  |  |  | |  |  |
| C1 | ACSL1 |  |  |  | |  |  |
| C1 | ZRANB2 |  |  |  | |  |  |
| C1 | INA |  |  |  | |  |  |
| C1 | PDCD5 |  |  |  | |  |  |
| C1 | SLC25A24 |  |  |  | |  |  |
| C1 | CCT2 |  |  |  | |  |  |
| C1 | MUC1 |  |  |  | |  |  |
| C1 | UQCR10 |  |  |  | |  |  |
| C1 | PA2G4 |  |  |  | |  |  |
| C1 | FNBP1L |  |  |  | |  |  |
| C1 | INPP4A |  |  |  | |  |  |
| C1 | GPD1L |  |  |  | |  |  |
| C1 | COX6C |  |  |  | |  |  |
| C1 | EIF1AX |  |  |  | |  |  |
| C1 | MORF4L2 |  |  |  | |  |  |
| C1 | FASN |  |  |  | |  |  |
| C1 | TMEM147 |  |  |  | |  |  |
| C1 | AMFR |  |  |  | |  |  |
| C1 | SRSF1 |  |  |  | |  |  |
| C1 | DDT |  |  |  | |  |  |
| C1 | MX2 |  |  |  | |  |  |
| C1 | C6orf62 |  |  |  | |  |  |
| C1 | FCRLB |  |  |  | |  |  |
| C1 | SLC25A36 |  |  |  | |  |  |
| C1 | SUMO3 |  |  |  | |  |  |
| C1 | ABHD12 |  |  |  | |  |  |
| C1 | CSTF3 |  |  |  | |  |  |
| C1 | RBM17 |  |  |  | |  |  |
| C1 | PPP3CA |  |  |  | |  |  |
| C1 | SERBP1 |  |  |  | |  |  |
| C1 | FAM89B |  |  |  | |  |  |
| C1 | TMCO1 |  |  |  | |  |  |
| C1 | GIPC1 |  |  |  | |  |  |
| C1 | TBX3 |  |  |  | |  |  |
| C1 | LAMTOR2 |  |  |  | |  |  |
| C1 | IER3IP1 |  |  |  | |  |  |
| C1 | CCNG2 |  |  |  | |  |  |
| C1 | DDX18 |  |  |  | |  |  |
| C1 | CCDC14 |  |  |  | |  |  |
| C1 | SERINC2 |  |  |  | |  |  |
| C1 | PMM1 |  |  |  | |  |  |
| C1 | TSPAN6 |  |  |  | |  |  |
| C1 | TMEM9 |  |  |  | |  |  |
| C1 | WDR24 |  |  |  | |  |  |
| C1 | EVPL |  |  |  | |  |  |
| C1 | EIF4EBP1 |  |  |  | |  |  |
| C1 | ATP6V0B |  |  |  | |  |  |
| C1 | LSM4 |  |  |  | |  |  |
| C1 | TMEM259 |  |  |  | |  |  |
| C1 | LMAN1 |  |  |  | |  |  |
| C1 | ITSN2 |  |  |  | |  |  |
| C1 | ARMCX6 |  |  |  | |  |  |
| C1 | HSPB8 |  |  |  | |  |  |
| C1 | KRT33B |  |  |  | |  |  |
| C1 | DCUN1D1 |  |  |  | |  |  |
| C1 | IRF2BP2 |  |  |  | |  |  |
| C1 | ADNP |  |  |  | |  |  |
| C1 | NR2F6 |  |  |  | |  |  |
| C1 | UBXN4 |  |  |  | |  |  |
| C1 | ZNF706 |  |  |  | |  |  |
| C1 | FXYD4 |  |  |  | |  |  |
| C1 | CBX3 |  |  |  | |  |  |
| C1 | COX6B1 |  |  |  | |  |  |
| C1 | MRPS26 |  |  |  | |  |  |
| C1 | PAK2 |  |  |  | |  |  |
| C1 | DUSP23 |  |  |  | |  |  |
| C1 | DDX17 |  |  |  | |  |  |
| C1 | URI1 |  |  |  | |  |  |
| C1 | MRPS34 |  |  |  | |  |  |
| C1 | GPD2 |  |  |  | |  |  |
| C1 | SPCS2 |  |  |  | |  |  |
| C1 | COX7B |  |  |  | |  |  |
| C1 | SUMO1 |  |  |  | |  |  |
| C1 | PLCD1 |  |  |  | |  |  |
| C1 | AGTRAP |  |  |  | |  |  |
| C1 | CRIP2 |  |  |  | |  |  |
| C1 | CERS6 |  |  |  | |  |  |
| C1 | RAB10 |  |  |  | |  |  |
| C1 | RTN3 |  |  |  | |  |  |
| C1 | RAP1GAP |  |  |  | |  |  |
| C1 | RAB25 |  |  |  | |  |  |
| C1 | ARF4 |  |  |  | |  |  |
| C1 | CSRP2 |  |  |  | |  |  |
| C1 | ELOVL1 |  |  |  | |  |  |
| C1 | KRCC1 |  |  |  | |  |  |
| C1 | UBE2E2 |  |  |  | |  |  |
| C1 | CCDC34 |  |  |  | |  |  |
| C1 | ARL5A |  |  |  | |  |  |
| C1 | AP3D1 |  |  |  | |  |  |
| C1 | PEG10 |  |  |  | |  |  |
| C1 | POLR2I |  |  |  | |  |  |
| C1 | BRD9 |  |  |  | |  |  |
| C1 | ROMO1 |  |  |  | |  |  |
| C1 | METRNL |  |  |  | |  |  |
| C1 | TMEM64 |  |  |  | |  |  |
| C1 | COX14 |  |  |  | |  |  |
| C1 | SCNN1G |  |  |  | |  |  |
| C1 | CAMTA1 |  |  |  | |  |  |
| C1 | YWHAQ |  |  |  | |  |  |
| C1 | UNC93B1 |  |  |  | |  |  |
| C1 | EFHD2 |  |  |  | |  |  |
| C1 | TINAGL1 |  |  |  | |  |  |
| C1 | CALU |  |  |  | |  |  |
| C1 | ACLY |  |  |  | |  |  |
| C1 | RNF19A |  |  |  | |  |  |
| C1 | CDYL |  |  |  | |  |  |
| C1 | ALDH1A3 |  |  |  | |  |  |
| C1 | LPCAT1 |  |  |  | |  |  |
| C1 | RIN2 |  |  |  | |  |  |
| C1 | JTB |  |  |  | |  |  |
| C1 | CISD3 |  |  |  | |  |  |
| C1 | HNRNPD |  |  |  | |  |  |
| C1 | RAB6A |  |  |  | |  |  |
| C1 | AP1B1 |  |  |  | |  |  |
| C1 | SNRPB2 |  |  |  | |  |  |
| C1 | SLC39A7 |  |  |  | |  |  |
| C1 | CTSD |  |  |  | |  |  |
| C1 | PRPS2 |  |  |  | |  |  |
| C1 | LGALS3BP |  |  |  | |  |  |
| C1 | PTMA |  |  |  | |  |  |
| C1 | TMA7 |  |  |  | |  |  |
| C1 | HAS3 |  |  |  | |  |  |
| C1 | SOX4 |  |  |  | |  |  |
| C1 | TMEM79 |  |  |  | |  |  |
| C1 | SPRR3 |  |  |  | |  |  |
| C1 | FAM3D |  |  |  | |  |  |
| C1 | PAFAH1B3 |  |  |  | |  |  |
| C1 | ATP6V1C1 |  |  |  | |  |  |
| C1 | PDCD6 |  |  |  | |  |  |
| C1 | ADAM15 |  |  |  | |  |  |
| C1 | ALOX15B |  |  |  | |  |  |
| C1 | FXYD3 |  |  |  | |  |  |
| C1 | PLOD2 |  |  |  | |  |  |
| C1 | LAD1 |  |  |  | |  |  |
| C1 | PSAP |  |  |  | |  |  |
| C1 | WDR1 |  |  |  | |  |  |
| C1 | GALNT1 |  |  |  | |  |  |
| C1 | CDV3 |  |  |  | |  |  |
| C1 | PPM1N |  |  |  | |  |  |
| C1 | GADD45GIP1 |  |  |  | |  |  |
| C1 | RAB2A |  |  |  | |  |  |
| C1 | PSMD7 |  |  |  | |  |  |
| C1 | UFM1 |  |  |  | |  |  |
| C1 | COX7A2 |  |  |  | |  |  |
| C1 | ESRP1 |  |  |  | |  |  |
| C1 | YOD1 |  |  |  | |  |  |
| C1 | PGRMC1 |  |  |  | |  |  |
| C1 | HNRNPAB |  |  |  | |  |  |
| C1 | SRRM2 |  |  |  | |  |  |
| C1 | FANCL |  |  |  | |  |  |
| C1 | GLRX5 |  |  |  | |  |  |
| C1 | NDUFA1 |  |  |  | |  |  |
| C1 | UBE2K |  |  |  | |  |  |
| C1 | MTCH1 |  |  |  | |  |  |
| C1 | CAPNS1 |  |  |  | |  |  |
| C1 | GMNN |  |  |  | |  |  |
| C1 | RPL39 |  |  |  | |  |  |
| C1 | GNL3 |  |  |  | |  |  |
| C1 | FAM78B |  |  |  | |  |  |
| C1 | ANKS1A |  |  |  | |  |  |
| C1 | PFDN2 |  |  |  | |  |  |
| C1 | TMEM87A |  |  |  | |  |  |
| C1 | C12orf75 |  |  |  | |  |  |
| C1 | CLPTM1L |  |  |  | |  |  |
| C1 | PTBP3 |  |  |  | |  |  |
| C1 | HNRNPL |  |  |  | |  |  |
| C1 | LSR |  |  |  | |  |  |
| C1 | SMC1A |  |  |  | |  |  |
| C1 | ZC3H11A |  |  |  | |  |  |
| C1 | DAZAP1 |  |  |  | |  |  |
| C1 | NDUFA11 |  |  |  | |  |  |
| C1 | IRF2BPL |  |  |  | |  |  |
| C1 | C1orf131 |  |  |  | |  |  |
| C1 | DHX40 |  |  |  | |  |  |
| C1 | COX8A |  |  |  | |  |  |
| C1 | SLC9A2 |  |  |  | |  |  |
| C1 | RPP25 |  |  |  | |  |  |
| C1 | MGST1 |  |  |  | |  |  |
| C1 | LSM7 |  |  |  | |  |  |
| C1 | GDI2 |  |  |  | |  |  |
| C1 | MT1X |  |  |  | |  |  |
| C1 | TFF3 |  |  |  | |  |  |
| C1 | SDF2L1 |  |  |  | |  |  |
| C1 | IMP4 |  |  |  | |  |  |
| C1 | SOCS2 |  |  |  | |  |  |
| C1 | CCT5 |  |  |  | |  |  |
| C1 | GDPD3 |  |  |  | |  |  |
| C1 | CAPN1 |  |  |  | |  |  |
| C1 | WTAP |  |  |  | |  |  |
| C1 | PHGDH |  |  |  | |  |  |
| C1 | ST3GAL4 |  |  |  | |  |  |
| C1 | BCAP31 |  |  |  | |  |  |
| C1 | STK24 |  |  |  | |  |  |
| C1 | TXN |  |  |  | |  |  |
| C1 | YY1 |  |  |  | |  |  |
| C1 | TUG1 |  |  |  | |  |  |
| C1 | SLTM |  |  |  | |  |  |
| C1 | DRAP1 |  |  |  | |  |  |
| C1 | GOLIM4 |  |  |  | |  |  |
| C1 | RNF2 |  |  |  | |  |  |
| C1 | UPF3B |  |  |  | |  |  |
| C1 | POLR2G |  |  |  | |  |  |
| C1 | SUB1 |  |  |  | |  |  |
| C1 | TEPP |  |  |  | |  |  |
| C1 | EIF3J |  |  |  | |  |  |
| C1 | ZFC3H1 |  |  |  | |  |  |
| C1 | SSR4 |  |  |  | |  |  |
| C1 | XAB2 |  |  |  | |  |  |
| C1 | DBI |  |  |  | |  |  |
| C1 | TNIP2 |  |  |  | |  |  |
| C1 | RER1 |  |  |  | |  |  |
| C1 | DENR |  |  |  | |  |  |
| C1 | NDUFB11 |  |  |  | |  |  |
| C1 | CDC34 |  |  |  | |  |  |
| C1 | SNCG |  |  |  | |  |  |
| C1 | CCDC167 |  |  |  | |  |  |
| C1 | TCEAL8 |  |  |  | |  |  |
| C1 | SMG7 |  |  |  | |  |  |
| C1 | ASCC2 |  |  |  | |  |  |
| C1 | MAD2L2 |  |  |  | |  |  |
| C1 | EIF6 |  |  |  | |  |  |
| C1 | TJP1 |  |  |  | |  |  |
| C1 | HMGN1 |  |  |  | |  |  |
| C1 | TAF11 |  |  |  | |  |  |
| C1 | HNRNPR |  |  |  | |  |  |
| C1 | GOT2 |  |  |  | |  |  |
| C1 | PLS1 |  |  |  | |  |  |
| C1 | SRPRB |  |  |  | |  |  |
| C1 | DHX36 |  |  |  | |  |  |
| C1 | POLE3 |  |  |  | |  |  |
| C1 | EIF2S2 |  |  |  | |  |  |
| C1 | TMEM109 |  |  |  | |  |  |
| C1 | RPS4Y1 |  |  |  | |  |  |
| C1 | OSTC |  |  |  | |  |  |
| C1 | AP2S1 |  |  |  | |  |  |
| C1 | ST14 |  |  |  | |  |  |
| C1 | TIA1 |  |  |  | |  |  |
| C1 | TSC22D2 |  |  |  | |  |  |
| C1 | PTBP1 |  |  |  | |  |  |
| C1 | DDA1 |  |  |  | |  |  |
| C1 | DYRK2 |  |  |  | |  |  |
| C1 | RALY |  |  |  | |  |  |
| C1 | GJA1 |  |  |  | |  |  |
| C1 | APH1A |  |  |  | |  |  |
| C1 | CPNE3 |  |  |  | |  |  |
| C1 | RPP38 |  |  |  | |  |  |
| C1 | ARL5B |  |  |  | |  |  |
| C1 | PVALB |  |  |  | |  |  |
| C1 | CST3 |  |  |  | |  |  |
| C1 | SLC4A7 |  |  |  | |  |  |
| C1 | CLCN3 |  |  |  | |  |  |
| C1 | MAP4K3 |  |  |  | |  |  |
| C1 | RAB27B |  |  |  | |  |  |
| C1 | SPSB3 |  |  |  | |  |  |
| C1 | USP31 |  |  |  | |  |  |
| C1 | YWHAB |  |  |  | |  |  |
| C1 | CCK |  |  |  | |  |  |
| C1 | UBE2G2 |  |  |  | |  |  |
| C1 | RBM25 |  |  |  | |  |  |
| C1 | ARL6IP5 |  |  |  | |  |  |
| C1 | FLYWCH2 |  |  |  | |  |  |
| C1 | ARMCX3 |  |  |  | |  |  |
| C1 | RPS10 |  |  |  | |  |  |
| C1 | FOXA1 |  |  |  | |  |  |
| C1 | CST6 |  |  |  | |  |  |
| C1 | UBE2G1 |  |  |  | |  |  |
| C1 | CIB1 |  |  |  | |  |  |
| C1 | EIF3B |  |  |  | |  |  |
| C1 | RPN2 |  |  |  | |  |  |
| C1 | MRPL12 |  |  |  | |  |  |
| C1 | YIF1A |  |  |  | |  |  |
| C1 | SMARCC1 |  |  |  | |  |  |
| C1 | MED13 |  |  |  | |  |  |
| C1 | PCMTD1 |  |  |  | |  |  |
| C1 | RABAC1 |  |  |  | |  |  |
| C1 | CAMK2G |  |  |  | |  |  |
| C1 | POLE4 |  |  |  | |  |  |
| C1 | YWHAG |  |  |  | |  |  |
| C1 | AXDND1 |  |  |  | |  |  |
| C1 | SNRPB |  |  |  | |  |  |
| C1 | METAP2 |  |  |  | |  |  |
| C1 | SEMA3C |  |  |  | |  |  |
| C1 | BPTF |  |  |  | |  |  |
| C1 | LAPTM4A |  |  |  | |  |  |
| C1 | DAP |  |  |  | |  |  |
| C1 | RAC2 |  |  |  | |  |  |
| C1 | DDAH1 |  |  |  | |  |  |
| C1 | WDR45B |  |  |  | |  |  |
| C1 | FAM210A |  |  |  | |  |  |
| C1 | DAB2 |  |  |  | |  |  |
| C1 | SRP9 |  |  |  | |  |  |
| C1 | RHOQ |  |  |  | |  |  |
| C1 | PFDN6 |  |  |  | |  |  |
| C1 | RAI14 |  |  |  | |  |  |
| C1 | UBE2W |  |  |  | |  |  |
| C1 | CDS1 |  |  |  | |  |  |
| C1 | PDIA6 |  |  |  | |  |  |
| C1 | GNAQ |  |  |  | |  |  |
| C1 | GOLT1B |  |  |  | |  |  |
| C1 | SRSF9 |  |  |  | |  |  |
| C1 | RPL37A |  |  |  | |  |  |
| C1 | HSPA1A |  |  |  | |  |  |
| C1 | PGRMC2 |  |  |  | |  |  |
| C1 | PHLDA3 |  |  |  | |  |  |
| C1 | PRMT2 |  |  |  | |  |  |
| C1 | RPS28 |  |  |  | |  |  |
| C1 | GJB2 |  |  |  | |  |  |
| C1 | STIM2 |  |  |  | |  |  |
| C1 | TBC1D1 |  |  |  | |  |  |
| C1 | ARL8B |  |  |  | |  |  |
| C1 | LSM14A |  |  |  | |  |  |
| C1 | TXN2 |  |  |  | |  |  |
| C1 | PKN2 |  |  |  | |  |  |
| C1 | IKZF2 |  |  |  | |  |  |
| C1 | PSMD10 |  |  |  | |  |  |
| C1 | DGUOK |  |  |  | |  |  |
| C1 | CLDND1 |  |  |  | |  |  |
| C1 | ALDH3B2 |  |  |  | |  |  |
| C1 | LMNB1 |  |  |  | |  |  |
| C1 | PFN1 |  |  |  | |  |  |
| C1 | VGLL1 |  |  |  | |  |  |
| C1 | CDCA7 |  |  |  | |  |  |
| C1 | RNF128 |  |  |  | |  |  |
| C1 | DNAJA2 |  |  |  | |  |  |
| C1 | RBMS1 |  |  |  | |  |  |
| C1 | RBBP6 |  |  |  | |  |  |
| C1 | COX7C |  |  |  | |  |  |
| C1 | DSP |  |  |  | |  |  |
| C1 | RPN1 |  |  |  | |  |  |
| C1 | CTNNBIP1 |  |  |  | |  |  |
| C1 | ALCAM |  |  |  | |  |  |
| C1 | PSMB4 |  |  |  | |  |  |
| C1 | RAB1B |  |  |  | |  |  |
| C1 | FOSL2 |  |  |  | |  |  |
| C1 | DDX46 |  |  |  | |  |  |
| C1 | GALNT14 |  |  |  | |  |  |
| C1 | YAF2 |  |  |  | |  |  |
| C1 | DENND1B |  |  |  | |  |  |
| C1 | RPS27 |  |  |  | |  |  |
| C1 | RPL22 |  |  |  | |  |  |
| C1 | TCEAL4 |  |  |  | |  |  |
| C1 | IFI44L |  |  |  | |  |  |
| C1 | CD164 |  |  |  | |  |  |
| C1 | ARGLU1 |  |  |  | |  |  |
| C1 | BASP1 |  |  |  | |  |  |
| C1 | ZNF322 |  |  |  | |  |  |
| C1 | GUK1 |  |  |  | |  |  |
| C1 | ARL6IP1 |  |  |  | |  |  |
| C1 | SNRPG |  |  |  | |  |  |
| C1 | HMGXB4 |  |  |  | |  |  |
| C1 | FAM50A |  |  |  | |  |  |
| C1 | MIIP |  |  |  | |  |  |
| C1 | SCCPDH |  |  |  | |  |  |
| C1 | MAPK1 |  |  |  | |  |  |
| C1 | GATA3 |  |  |  | |  |  |
| C1 | SCAMP3 |  |  |  | |  |  |
| C1 | SCP2 |  |  |  | |  |  |
| C1 | BRD4 |  |  |  | |  |  |
| C1 | UXS1 |  |  |  | |  |  |
| C1 | RNF181 |  |  |  | |  |  |
| C1 | INSR |  |  |  | |  |  |
| C1 | UBALD2 |  |  |  | |  |  |
| C1 | MTMR4 |  |  |  | |  |  |
| C1 | DSTN |  |  |  | |  |  |
| C1 | DAPK1 |  |  |  | |  |  |
| C1 | DPM2 |  |  |  | |  |  |
| C1 | C1D |  |  |  | |  |  |
| C1 | RAN |  |  |  | |  |  |
| C1 | NDUFB10 |  |  |  | |  |  |
| C1 | RASSF3 |  |  |  | |  |  |
| C1 | UBXN6 |  |  |  | |  |  |
| C1 | ITGAV |  |  |  | |  |  |
| C1 | PLEKHA3 |  |  |  | |  |  |
| C1 | IFT27 |  |  |  | |  |  |
| C1 | MRPL14 |  |  |  | |  |  |
| C1 | NEK7 |  |  |  | |  |  |
| C1 | CITED2 |  |  |  | |  |  |
| C1 | ARPC5L |  |  |  | |  |  |
| C1 | CTDNEP1 |  |  |  | |  |  |
| C1 | GSTM4 |  |  |  | |  |  |
| C1 | MRPL55 |  |  |  | |  |  |
| C1 | AFG3L2 |  |  |  | |  |  |
| C1 | EMC6 |  |  |  | |  |  |
| C1 | SURF6 |  |  |  | |  |  |
| C1 | APOL4 |  |  |  | |  |  |
| C1 | YTHDF2 |  |  |  | |  |  |
| C1 | TMEM50A |  |  |  | |  |  |
| C1 | SUN2 |  |  |  | |  |  |
| C1 | CYP2J2 |  |  |  | |  |  |
| C1 | RPL11 |  |  |  | |  |  |
| C1 | DCTPP1 |  |  |  | |  |  |
| C1 | XPOT |  |  |  | |  |  |
| C1 | GNG5 |  |  |  | |  |  |
| C1 | UBE2A |  |  |  | |  |  |
| C1 | CCNE1 |  |  |  | |  |  |
| C1 | ACP1 |  |  |  | |  |  |
| C1 | CEP350 |  |  |  | |  |  |
| C1 | RPL41 |  |  |  | |  |  |
| C1 | S100A8 |  |  |  | |  |  |
| C1 | COX6A1 |  |  |  | |  |  |
| C1 | CDC37 |  |  |  | |  |  |
| C1 | NDRG1 |  |  |  | |  |  |
| C1 | AZIN1 |  |  |  | |  |  |
| C1 | ACOT7 |  |  |  | |  |  |
| C1 | NOTCH2 |  |  |  | |  |  |
| C1 | SHC1 |  |  |  | |  |  |
| C1 | ADIRF |  |  |  | |  |  |
| C1 | CHD7 |  |  |  | |  |  |
| C1 | FRS2 |  |  |  | |  |  |
| C1 | BST1 |  |  |  | |  |  |
| C1 | CSNK1G2 |  |  |  | |  |  |
| C1 | ZNF518A |  |  |  | |  |  |
| C2 | DEFB1 |  |  |  | |  |  |
| C2 | ZFAND2A |  |  |  | |  |  |
| C2 | INSIG1 |  |  |  | |  |  |
| C2 | CCND1 |  |  |  | |  |  |
| C2 | SERPINE1 |  |  |  | |  |  |
| C2 | IFI27 |  |  |  | |  |  |
| C2 | SULT1E1 |  |  |  | |  |  |
| C2 | TM4SF1 |  |  |  | |  |  |
| C2 | LEAP2 |  |  |  | |  |  |
| C2 | BMP2 |  |  |  | |  |  |
| C2 | RHOV |  |  |  | |  |  |
| C2 | PHLDA2 |  |  |  | |  |  |
| C2 | IFI6 |  |  |  | |  |  |
| C2 | PLAU |  |  |  | |  |  |
| C2 | S100A8 |  |  |  | |  |  |
| C2 | LY96 |  |  |  | |  |  |
| C2 | CITED4 |  |  |  | |  |  |
| C2 | CRABP2 |  |  |  | |  |  |
| C2 | IDI1 |  |  |  | |  |  |
| C2 | HSPA1B |  |  |  | |  |  |
| C2 | CA2 |  |  |  | |  |  |
| C2 | SQLE |  |  |  | |  |  |
| C2 | RNF39 |  |  |  | |  |  |
| C2 | BAG3 |  |  |  | |  |  |
| C2 | CYP3A5 |  |  |  | |  |  |
| C2 | HMGCS1 |  |  |  | |  |  |
| C2 | PI3 |  |  |  | |  |  |
| C2 | MT1X |  |  |  | |  |  |
| C2 | TMEM45A |  |  |  | |  |  |
| C2 | EPSTI1 |  |  |  | |  |  |
| C2 | MYC |  |  |  | |  |  |
| C2 | TYMP |  |  |  | |  |  |
| C2 | BPGM |  |  |  | |  |  |
| C2 | SPTSSA |  |  |  | |  |  |
| C2 | BLVRB |  |  |  | |  |  |
| C2 | SFN |  |  |  | |  |  |
| C2 | TUBA4A |  |  |  | |  |  |
| C2 | RAB34 |  |  |  | |  |  |
| C2 | S100A7 |  |  |  | |  |  |
| C2 | RGS16 |  |  |  | |  |  |
| C2 | LY6K |  |  |  | |  |  |
| C2 | RPA3 |  |  |  | |  |  |
| C2 | ACAT2 |  |  |  | |  |  |
| C2 | FOXN1 |  |  |  | |  |  |
| C2 | IL1A |  |  |  | |  |  |
| C2 | DUSP6 |  |  |  | |  |  |
| C2 | DNAJB4 |  |  |  | |  |  |
| C2 | TMEM97 |  |  |  | |  |  |
| C2 | EDN1 |  |  |  | |  |  |
| C2 | CXCL8 |  |  |  | |  |  |
| C2 | S100A9 |  |  |  | |  |  |
| C2 | KRT6A |  |  |  | |  |  |
| C2 | HSPB1 |  |  |  | |  |  |
| C2 | MAP2K3 |  |  |  | |  |  |
| C2 | SAT1 |  |  |  | |  |  |
| C2 | CDKN1A |  |  |  | |  |  |
| C2 | IFRD1 |  |  |  | |  |  |
| C2 | CSTA |  |  |  | |  |  |
| C2 | DBI |  |  |  | |  |  |
| C2 | GSTM4 |  |  |  | |  |  |
| C2 | CD40 |  |  |  | |  |  |
| C2 | FDFT1 |  |  |  | |  |  |
| C2 | CDC42EP4 |  |  |  | |  |  |
| C2 | C3orf14 |  |  |  | |  |  |
| C2 | PLK2 |  |  |  | |  |  |
| C2 | HSD17B6 |  |  |  | |  |  |
| C2 | SCD |  |  |  | |  |  |
| C2 | ARL4D |  |  |  | |  |  |
| C2 | DDIT3 |  |  |  | |  |  |
| C2 | HES1 |  |  |  | |  |  |
| C2 | SLPI |  |  |  | |  |  |
| C2 | TPCN2 |  |  |  | |  |  |
| C2 | CNN3 |  |  |  | |  |  |
| C2 | RHOB |  |  |  | |  |  |
| C2 | CALR |  |  |  | |  |  |
| C2 | PLTP |  |  |  | |  |  |
| C2 | PM20D1 |  |  |  | |  |  |
| C2 | CAPG |  |  |  | |  |  |
| C2 | ZC3H12A |  |  |  | |  |  |
| C2 | IFI16 |  |  |  | |  |  |
| C2 | DNAJC15 |  |  |  | |  |  |
| C2 | FOXJ1 |  |  |  | |  |  |
| C2 | WTAP |  |  |  | |  |  |
| C2 | GPX1 |  |  |  | |  |  |
| C2 | RIN2 |  |  |  | |  |  |
| C2 | SDC4 |  |  |  | |  |  |
| C2 | METTL1 |  |  |  | |  |  |
| C2 | TSPAN13 |  |  |  | |  |  |
| C2 | AP2S1 |  |  |  | |  |  |
| C2 | THAP2 |  |  |  | |  |  |
| C2 | AMFR |  |  |  | |  |  |
| C2 | SDF2L1 |  |  |  | |  |  |
| C2 | CD83 |  |  |  | |  |  |
| C2 | GCHFR |  |  |  | |  |  |
| C2 | SERTAD4 |  |  |  | |  |  |
| C2 | S100A13 |  |  |  | |  |  |
| C2 | KLF6 |  |  |  | |  |  |
| C2 | TMEM208 |  |  |  | |  |  |
| C2 | MKNK2 |  |  |  | |  |  |
| C2 | HSPA8 |  |  |  | |  |  |
| C2 | GLRX |  |  |  | |  |  |
| C2 | LGALS1 |  |  |  | |  |  |
| C2 | SERPINB4 |  |  |  | |  |  |
| C2 | MRPS6 |  |  |  | |  |  |
| C2 | MGLL |  |  |  | |  |  |
| C2 | CKLF |  |  |  | |  |  |
| C2 | FABP6 |  |  |  | |  |  |
| C2 | FAM3C |  |  |  | |  |  |
| C2 | MICALL2 |  |  |  | |  |  |
| C2 | POLR2J |  |  |  | |  |  |
| C2 | UQCRC1 |  |  |  | |  |  |
| C2 | FTL |  |  |  | |  |  |
| C2 | DEDD2 |  |  |  | |  |  |
| C2 | TSC22D1 |  |  |  | |  |  |
| C2 | TRA2A |  |  |  | |  |  |
| C2 | TRAF4 |  |  |  | |  |  |
| C2 | SLC20A1 |  |  |  | |  |  |
| C2 | LRRC61 |  |  |  | |  |  |
| C2 | RAC1 |  |  |  | |  |  |
| C2 | FXYD5 |  |  |  | |  |  |
| C2 | C1orf56 |  |  |  | |  |  |
| C2 | ID1 |  |  |  | |  |  |
| C2 | PSME2 |  |  |  | |  |  |
| C2 | CEBPB |  |  |  | |  |  |
| C2 | VTCN1 |  |  |  | |  |  |
| C2 | FA2H |  |  |  | |  |  |
| C2 | DSTN |  |  |  | |  |  |
| C2 | SERPINB3 |  |  |  | |  |  |
| C2 | FOS |  |  |  | |  |  |
| C2 | PRSS8 |  |  |  | |  |  |
| C2 | IRX3 |  |  |  | |  |  |
| C2 | DNAJA4 |  |  |  | |  |  |
| C2 | DUSP14 |  |  |  | |  |  |
| C2 | NUDT1 |  |  |  | |  |  |
| C2 | SCCPDH |  |  |  | |  |  |
| C2 | MAFB |  |  |  | |  |  |
| C2 | JOSD2 |  |  |  | |  |  |
| C2 | TMEM160 |  |  |  | |  |  |
| C2 | NPC2 |  |  |  | |  |  |
| C2 | IFI27L2 |  |  |  | |  |  |
| C2 | FRS2 |  |  |  | |  |  |
| C2 | MAP1LC3B |  |  |  | |  |  |
| C2 | RAB32 |  |  |  | |  |  |
| C2 | ZFC3H1 |  |  |  | |  |  |
| C2 | LGALS9 |  |  |  | |  |  |
| C2 | BTG2 |  |  |  | |  |  |
| C2 | LAMTOR4 |  |  |  | |  |  |
| C2 | FXYD3 |  |  |  | |  |  |
| C2 | PSCA |  |  |  | |  |  |
| C2 | PLCXD2 |  |  |  | |  |  |
| C2 | NUCKS1 |  |  |  | |  |  |
| C2 | BUD31 |  |  |  | |  |  |
| C2 | FABP5 |  |  |  | |  |  |
| C2 | SGK2 |  |  |  | |  |  |
| C2 | CCDC12 |  |  |  | |  |  |
| C2 | TNFRSF12A |  |  |  | |  |  |
| C2 | CST3 |  |  |  | |  |  |
| C2 | SRSF2 |  |  |  | |  |  |
| C2 | GADD45A |  |  |  | |  |  |
| C2 | KRT19 |  |  |  | |  |  |
| C2 | CHMP2B |  |  |  | |  |  |
| C2 | GMNN |  |  |  | |  |  |
| C2 | ZG16B |  |  |  | |  |  |
| C2 | KDELR2 |  |  |  | |  |  |
| C2 | MMP1 |  |  |  | |  |  |
| C2 | RHEB |  |  |  | |  |  |
| C2 | IGFBP7 |  |  |  | |  |  |
| C2 | MMP13 |  |  |  | |  |  |
| C2 | CDC42SE1 |  |  |  | |  |  |
| C2 | ICAM1 |  |  |  | |  |  |
| C2 | TMSB4X |  |  |  | |  |  |
| C2 | HSPH1 |  |  |  | |  |  |
| C2 | JUN |  |  |  | |  |  |
| C2 | SNX3 |  |  |  | |  |  |
| C2 | SRSF7 |  |  |  | |  |  |
| C2 | S100A14 |  |  |  | |  |  |
| C2 | LAGE3 |  |  |  | |  |  |
| C2 | SRSF6 |  |  |  | |  |  |
| C2 | SDF2 |  |  |  | |  |  |
| C2 | POMP |  |  |  | |  |  |
| C2 | NFKBIA |  |  |  | |  |  |
| C2 | EIF2S2 |  |  |  | |  |  |
| C2 | HSPE1 |  |  |  | |  |  |
| C2 | PTMS |  |  |  | |  |  |
| C2 | GNPDA1 |  |  |  | |  |  |
| C2 | PSMB3 |  |  |  | |  |  |
| C2 | PPIC |  |  |  | |  |  |
| C2 | SCAMP5 |  |  |  | |  |  |
| C2 | CALM1 |  |  |  | |  |  |
| C2 | ERRFI1 |  |  |  | |  |  |
| C2 | ERF |  |  |  | |  |  |
| C2 | ELOVL5 |  |  |  | |  |  |
| C2 | BAMBI |  |  |  | |  |  |
| C2 | GLRX3 |  |  |  | |  |  |
| C2 | MIDN |  |  |  | |  |  |
| C2 | MMP28 |  |  |  | |  |  |
| C2 | KRT10 |  |  |  | |  |  |
| C2 | KTN1 |  |  |  | |  |  |
| C2 | HMGB2 |  |  |  | |  |  |
| C2 | EGLN3 |  |  |  | |  |  |
| C2 | HEXB |  |  |  | |  |  |
| C2 | IFT140 |  |  |  | |  |  |
| C2 | POLDIP2 |  |  |  | |  |  |
| C2 | GTF3A |  |  |  | |  |  |
| C2 | CTNNB1 |  |  |  | |  |  |
| C2 | PSMD8 |  |  |  | |  |  |
| C2 | TAGLN2 |  |  |  | |  |  |
| C2 | PRDX5 |  |  |  | |  |  |
| C2 | RALA |  |  |  | |  |  |
| C2 | FIS1 |  |  |  | |  |  |
| C2 | AKR1C3 |  |  |  | |  |  |
| C2 | MUC1 |  |  |  | |  |  |
| C2 | TMEM37 |  |  |  | |  |  |
| C2 | CROT |  |  |  | |  |  |
| C2 | TRADD |  |  |  | |  |  |
| C2 | S100A11 |  |  |  | |  |  |
| C2 | RRAS |  |  |  | |  |  |
| C2 | LITAF |  |  |  | |  |  |
| C2 | WFDC2 |  |  |  | |  |  |
| C2 | ANXA4 |  |  |  | |  |  |
| C2 | ARF4 |  |  |  | |  |  |
| C2 | MPC2 |  |  |  | |  |  |
| C2 | UQCC2 |  |  |  | |  |  |
| C2 | TPP1 |  |  |  | |  |  |
| C2 | ADRM1 |  |  |  | |  |  |
| C2 | RPS4Y1 |  |  |  | |  |  |
| C2 | PPP3CA |  |  |  | |  |  |
| C2 | TPI1 |  |  |  | |  |  |
| C2 | LGALS3 |  |  |  | |  |  |
| C2 | MRPL27 |  |  |  | |  |  |
| C2 | CHCHD2 |  |  |  | |  |  |
| C2 | HSP90AB1 |  |  |  | |  |  |
| C2 | LMO4 |  |  |  | |  |  |
| C2 | PIFO |  |  |  | |  |  |
| C2 | KRT7 |  |  |  | |  |  |
| C2 | EGR1 |  |  |  | |  |  |
| C2 | SPRR1B |  |  |  | |  |  |
| C2 | HNRNPH1 |  |  |  | |  |  |
| C2 | BTG1 |  |  |  | |  |  |
| C2 | COX5A |  |  |  | |  |  |
| C2 | IL23A |  |  |  | |  |  |
| C2 | PDIA6 |  |  |  | |  |  |
| C2 | CCL20 |  |  |  | |  |  |
| C2 | MALSU1 |  |  |  | |  |  |
| C2 | TRIP6 |  |  |  | |  |  |
| C2 | SOD3 |  |  |  | |  |  |
| C2 | FTH1 |  |  |  | |  |  |
| C2 | MRPL18 |  |  |  | |  |  |
| C2 | CACYBP |  |  |  | |  |  |
| C2 | YWHAG |  |  |  | |  |  |
| C2 | MCL1 |  |  |  | |  |  |
| C2 | DRAP1 |  |  |  | |  |  |
| C2 | GPX4 |  |  |  | |  |  |
| C2 | JUP |  |  |  | |  |  |
| C2 | YWHAB |  |  |  | |  |  |
| C2 | ONECUT2 |  |  |  | |  |  |
| C2 | DYNLRB1 |  |  |  | |  |  |
| C2 | RIT1 |  |  |  | |  |  |
| C2 | PDZK1IP1 |  |  |  | |  |  |
| C2 | QPCT |  |  |  | |  |  |
| C2 | TMEM120A |  |  |  | |  |  |
| C2 | CCT2 |  |  |  | |  |  |
| C2 | GRN |  |  |  | |  |  |
| C2 | NAA20 |  |  |  | |  |  |
| C2 | SLC35B1 |  |  |  | |  |  |
| C2 | CUTA |  |  |  | |  |  |
| C2 | COX7A2 |  |  |  | |  |  |
| C2 | COPE |  |  |  | |  |  |
| C2 | ATP6V1F |  |  |  | |  |  |
| C2 | KIAA0040 |  |  |  | |  |  |
| C2 | KLF10 |  |  |  | |  |  |
| C2 | ZDHHC4 |  |  |  | |  |  |
| C2 | TEX264 |  |  |  | |  |  |
| C2 | BLVRA |  |  |  | |  |  |
| C2 | MIEN1 |  |  |  | |  |  |
| C2 | GAPDH |  |  |  | |  |  |
| C2 | FOSB |  |  |  | |  |  |
| C2 | PSMC2 |  |  |  | |  |  |
| C2 | COPRS |  |  |  | |  |  |
| C2 | NXT1 |  |  |  | |  |  |
| C2 | RBCK1 |  |  |  | |  |  |
| C2 | DUSP1 |  |  |  | |  |  |
| C2 | PSMC3 |  |  |  | |  |  |
| C2 | GUK1 |  |  |  | |  |  |
| C2 | CYP4B1 |  |  |  | |  |  |
| C2 | ARPC2 |  |  |  | |  |  |
| C2 | EFNA1 |  |  |  | |  |  |
| C2 | S100P |  |  |  | |  |  |
| C2 | LSM2 |  |  |  | |  |  |
| C2 | ANKRD22 |  |  |  | |  |  |
| C2 | SLBP |  |  |  | |  |  |
| C2 | CENPW |  |  |  | |  |  |
| C2 | ATP1B3 |  |  |  | |  |  |
| C2 | EIF4EBP1 |  |  |  | |  |  |
| C2 | MAFF |  |  |  | |  |  |
| C2 | CDK5 |  |  |  | |  |  |
| C2 | CMC2 |  |  |  | |  |  |
| C2 | ADM |  |  |  | |  |  |
| C2 | ZFP36L1 |  |  |  | |  |  |
| C2 | TLCD1 |  |  |  | |  |  |
| C2 | MKKS |  |  |  | |  |  |
| C2 | HSD17B14 |  |  |  | |  |  |
| C2 | SPAG4 |  |  |  | |  |  |
| C2 | DUSP23 |  |  |  | |  |  |
| C2 | PHYH |  |  |  | |  |  |
| C2 | SRSF3 |  |  |  | |  |  |
| C2 | ID3 |  |  |  | |  |  |
| C2 | ZFP36 |  |  |  | |  |  |
| C2 | TMEM14A |  |  |  | |  |  |
| C2 | ZYX |  |  |  | |  |  |
| C2 | RPLP1 |  |  |  | |  |  |
| C2 | FBLN1 |  |  |  | |  |  |
| C2 | SNRPB |  |  |  | |  |  |
| C2 | SHISA5 |  |  |  | |  |  |
| C2 | RPLP2 |  |  |  | |  |  |
| C2 | ATP6V0B |  |  |  | |  |  |
| C2 | TRAPPC2L |  |  |  | |  |  |
| C2 | CCDC167 |  |  |  | |  |  |
| C2 | YEATS4 |  |  |  | |  |  |
| C2 | CDK2AP2 |  |  |  | |  |  |
| C2 | PRDX1 |  |  |  | |  |  |
| C2 | CEBPD |  |  |  | |  |  |
| C2 | UBC |  |  |  | |  |  |
| C2 | TMA7 |  |  |  | |  |  |
| C2 | RABAC1 |  |  |  | |  |  |
| C2 | HEXIM1 |  |  |  | |  |  |
| C2 | ATF4 |  |  |  | |  |  |
| C2 | RPL19 |  |  |  | |  |  |
| C2 | RIPK4 |  |  |  | |  |  |
| C2 | EBP |  |  |  | |  |  |
| C2 | VASP |  |  |  | |  |  |
| C2 | TMED10 |  |  |  | |  |  |
| C2 | SRP14 |  |  |  | |  |  |
| C2 | MRPL55 |  |  |  | |  |  |
| C2 | MPHOSPH6 |  |  |  | |  |  |
| C2 | CYB5R1 |  |  |  | |  |  |
| C2 | NSFL1C |  |  |  | |  |  |
| C2 | SLC25A39 |  |  |  | |  |  |
| C2 | PTTG1 |  |  |  | |  |  |
| C2 | SPATA2L |  |  |  | |  |  |
| C2 | RPL23A |  |  |  | |  |  |
| C2 | BCAS4 |  |  |  | |  |  |
| C2 | THBD |  |  |  | |  |  |
| C2 | TSFM |  |  |  | |  |  |
| C2 | CDKN2C |  |  |  | |  |  |
| C2 | ETHE1 |  |  |  | |  |  |
| C2 | TMEM60 |  |  |  | |  |  |
| C2 | KIF5B |  |  |  | |  |  |
| C2 | PSMB1 |  |  |  | |  |  |
| C2 | DHRS13 |  |  |  | |  |  |
| C2 | ATP6V0E1 |  |  |  | |  |  |
| C2 | SF3B5 |  |  |  | |  |  |
| C2 | NFKBIZ |  |  |  | |  |  |
| C2 | COMMD7 |  |  |  | |  |  |
| C2 | FCGRT |  |  |  | |  |  |
| C2 | CLDN1 |  |  |  | |  |  |
| C2 | CSTB |  |  |  | |  |  |
| C2 | FAU |  |  |  | |  |  |
| C2 | RPL8 |  |  |  | |  |  |
| C2 | MOSPD3 |  |  |  | |  |  |
| C2 | HOXD1 |  |  |  | |  |  |
| C2 | JUNB |  |  |  | |  |  |
| C2 | RPS5 |  |  |  | |  |  |
| C2 | UNC119 |  |  |  | |  |  |
| C2 | ROMO1 |  |  |  | |  |  |
| C2 | SLC25A5 |  |  |  | |  |  |
| C2 | BATF |  |  |  | |  |  |
| C2 | PFDN4 |  |  |  | |  |  |
| C2 | OSER1 |  |  |  | |  |  |
| C2 | PIK3R3 |  |  |  | |  |  |
| C2 | TIMM10B |  |  |  | |  |  |
| C2 | COX7C |  |  |  | |  |  |
| C2 | COX6B1 |  |  |  | |  |  |
| C2 | PRSS22 |  |  |  | |  |  |
| C2 | RAB7A |  |  |  | |  |  |
| C2 | KIAA1217 |  |  |  | |  |  |
| C2 | SET |  |  |  | |  |  |
| C2 | PSMD7 |  |  |  | |  |  |
| C2 | ERAL1 |  |  |  | |  |  |
| C2 | SNRPE |  |  |  | |  |  |
| C2 | CCDC25 |  |  |  | |  |  |
| C2 | DAD1 |  |  |  | |  |  |
| C2 | ALAS1 |  |  |  | |  |  |
| C2 | UQCR10 |  |  |  | |  |  |
| C2 | TCEA3 |  |  |  | |  |  |
| C2 | MGMT |  |  |  | |  |  |
| C2 | RHBDD2 |  |  |  | |  |  |
| C2 | CLEC2B |  |  |  | |  |  |
| C2 | TOB1 |  |  |  | |  |  |
| C2 | PLA2G2F |  |  |  | |  |  |
| C2 | TMEM147 |  |  |  | |  |  |
| C2 | MRPL54 |  |  |  | |  |  |
| C2 | EFNB1 |  |  |  | |  |  |
| C2 | ETF1 |  |  |  | |  |  |
| C2 | BCL2L12 |  |  |  | |  |  |
| C2 | MAP3K8 |  |  |  | |  |  |
| C2 | MSX2 |  |  |  | |  |  |
| C2 | SRP72 |  |  |  | |  |  |
| C2 | CLCA2 |  |  |  | |  |  |
| C2 | CDC42EP1 |  |  |  | |  |  |
| C2 | SUB1 |  |  |  | |  |  |
| C2 | DGCR6L |  |  |  | |  |  |
| C2 | NDUFB10 |  |  |  | |  |  |
| C2 | IRF2BPL |  |  |  | |  |  |
| C2 | CLIC1 |  |  |  | |  |  |
| C2 | OS9 |  |  |  | |  |  |
| C2 | VKORC1 |  |  |  | |  |  |
| C2 | CCDC124 |  |  |  | |  |  |
| C2 | COX17 |  |  |  | |  |  |
| C2 | BAX |  |  |  | |  |  |
| C2 | CXXC5 |  |  |  | |  |  |
| C2 | PSMA1 |  |  |  | |  |  |
| C2 | COX8A |  |  |  | |  |  |
| C2 | SERTAD3 |  |  |  | |  |  |
| C2 | SNRPD2 |  |  |  | |  |  |
| C2 | RPL18A |  |  |  | |  |  |
| C2 | PSMA3 |  |  |  | |  |  |
| C2 | NDUFA13 |  |  |  | |  |  |
| C2 | CD63 |  |  |  | |  |  |
| C2 | SUMO1 |  |  |  | |  |  |
| C2 | MYL6 |  |  |  | |  |  |
| C2 | LAMTOR2 |  |  |  | |  |  |
| C2 | TIMP3 |  |  |  | |  |  |
| C2 | ACTG1 |  |  |  | |  |  |
| C2 | TRIB1 |  |  |  | |  |  |
| C2 | RPL10 |  |  |  | |  |  |
| C2 | RBX1 |  |  |  | |  |  |
| C2 | RPL27 |  |  |  | |  |  |
| C2 | TFAP2C |  |  |  | |  |  |
| C2 | COX7B |  |  |  | |  |  |
| C2 | TMEM126A |  |  |  | |  |  |
| C2 | ERP27 |  |  |  | |  |  |
| C2 | DYNLT1 |  |  |  | |  |  |
| C2 | BAZ1A |  |  |  | |  |  |
| C2 | RPS13 |  |  |  | |  |  |
| C2 | CISD1 |  |  |  | |  |  |
| C2 | DSP |  |  |  | |  |  |
| C2 | BABAM1 |  |  |  | |  |  |
| C2 | CENPH |  |  |  | |  |  |
| C2 | NDUFA11 |  |  |  | |  |  |
| C2 | HMGCS2 |  |  |  | |  |  |
| C2 | NDUFB3 |  |  |  | |  |  |
| C2 | PPP1R7 |  |  |  | |  |  |
| C2 | RPS14 |  |  |  | |  |  |
| C2 | MTCH2 |  |  |  | |  |  |
| C2 | PAFAH1B3 |  |  |  | |  |  |
| C2 | C12orf57 |  |  |  | |  |  |
| C2 | CBX3 |  |  |  | |  |  |
| C2 | SAP18 |  |  |  | |  |  |
| C2 | SNRPG |  |  |  | |  |  |
| C2 | RNF114 |  |  |  | |  |  |
| C2 | CTSD |  |  |  | |  |  |
| C2 | AMZ2 |  |  |  | |  |  |
| C2 | RPS16 |  |  |  | |  |  |
| C2 | UBE2T |  |  |  | |  |  |
| C2 | SFT2D1 |  |  |  | |  |  |
| C2 | CISD2 |  |  |  | |  |  |
| C2 | USP16 |  |  |  | |  |  |
| C2 | TUSC2 |  |  |  | |  |  |
| C2 | SERP1 |  |  |  | |  |  |
| C2 | RPS28 |  |  |  | |  |  |
| C2 | CCNL1 |  |  |  | |  |  |
| C2 | ITM2B |  |  |  | |  |  |
| C2 | RPL34 |  |  |  | |  |  |
| C2 | BZW2 |  |  |  | |  |  |
| C2 | SPRY1 |  |  |  | |  |  |
| C2 | MARCKS |  |  |  | |  |  |
| C2 | MAPRE1 |  |  |  | |  |  |
| C2 | PTP4A1 |  |  |  | |  |  |
| C2 | RIPK2 |  |  |  | |  |  |
| C2 | CLDN4 |  |  |  | |  |  |
| C2 | MFSD2A |  |  |  | |  |  |
| C2 | HYAL2 |  |  |  | |  |  |
| C2 | EFHD2 |  |  |  | |  |  |
| C2 | UBE2L6 |  |  |  | |  |  |
| C2 | PSMC5 |  |  |  | |  |  |
| C2 | GABARAPL2 |  |  |  | |  |  |
| C2 | TMUB1 |  |  |  | |  |  |
| C2 | RPS12 |  |  |  | |  |  |
| C2 | NDUFS5 |  |  |  | |  |  |
| C2 | TSPAN15 |  |  |  | |  |  |
| C2 | RPL18 |  |  |  | |  |  |
| C2 | POLR3GL |  |  |  | |  |  |
| C2 | ARRDC3 |  |  |  | |  |  |
| C2 | KMT2E |  |  |  | |  |  |
| C2 | RALY |  |  |  | |  |  |
| C2 | SNRPB2 |  |  |  | |  |  |
| C2 | PLSCR1 |  |  |  | |  |  |
| C2 | MPV17 |  |  |  | |  |  |
| C2 | GADD45GIP1 |  |  |  | |  |  |
| C2 | PSMF1 |  |  |  | |  |  |
| C2 | MRPS12 |  |  |  | |  |  |
| C2 | JTB |  |  |  | |  |  |
| C2 | EMC9 |  |  |  | |  |  |
| C2 | SSR3 |  |  |  | |  |  |
| C2 | ANP32E |  |  |  | |  |  |
| C2 | TIMM13 |  |  |  | |  |  |
| C2 | DNPH1 |  |  |  | |  |  |
| C2 | TNFAIP1 |  |  |  | |  |  |
| C2 | EMC7 |  |  |  | |  |  |
| C2 | BCL7B |  |  |  | |  |  |
| C2 | NDUFB11 |  |  |  | |  |  |
| C2 | PMVK |  |  |  | |  |  |
| C2 | UBE2B |  |  |  | |  |  |
| C2 | ADNP |  |  |  | |  |  |
| C2 | BTG3 |  |  |  | |  |  |
| C2 | MRPL12 |  |  |  | |  |  |
| C2 | SPINT2 |  |  |  | |  |  |
| C2 | EHF |  |  |  | |  |  |
| C2 | PERP |  |  |  | |  |  |
| C2 | MRPL17 |  |  |  | |  |  |
| C2 | DNAJB11 |  |  |  | |  |  |
| C2 | ARL6IP1 |  |  |  | |  |  |
| C2 | PTMA |  |  |  | |  |  |
| C2 | PMEPA1 |  |  |  | |  |  |
| C2 | EIF6 |  |  |  | |  |  |
| C2 | SULT2A1 |  |  |  | |  |  |
| C2 | RPL37A |  |  |  | |  |  |
| C2 | MYEOV |  |  |  | |  |  |
| C2 | SF3B6 |  |  |  | |  |  |
| C2 | DPY30 |  |  |  | |  |  |
| C2 | FAM110A |  |  |  | |  |  |
| C2 | SIKE1 |  |  |  | |  |  |
| C2 | PSMB5 |  |  |  | |  |  |
| C2 | DDX5 |  |  |  | |  |  |
| C2 | RPS2 |  |  |  | |  |  |
| C2 | OSTC |  |  |  | |  |  |
| C2 | HOMER3 |  |  |  | |  |  |
| C2 | PDIA4 |  |  |  | |  |  |
| C2 | CHPF |  |  |  | |  |  |
| C2 | ATRAID |  |  |  | |  |  |
| C2 | RPS27 |  |  |  | |  |  |
| C2 | CYC1 |  |  |  | |  |  |
| C2 | ISOC2 |  |  |  | |  |  |
| C2 | TUBA1B |  |  |  | |  |  |
| C2 | TPT1 |  |  |  | |  |  |
| C2 | PLP2 |  |  |  | |  |  |
| C2 | SYPL1 |  |  |  | |  |  |
| C2 | CYSTM1 |  |  |  | |  |  |
| C2 | PSMB8 |  |  |  | |  |  |
| C2 | PEPD |  |  |  | |  |  |
| C2 | PTPRS |  |  |  | |  |  |
| C2 | KRT8 |  |  |  | |  |  |
| C2 | PUF60 |  |  |  | |  |  |
| C2 | PFDN2 |  |  |  | |  |  |
| C2 | RTN4 |  |  |  | |  |  |
| C2 | PHLDA3 |  |  |  | |  |  |
| C2 | RPL36AL |  |  |  | |  |  |
| C2 | ERH |  |  |  | |  |  |
| C2 | SMDT1 |  |  |  | |  |  |
| C3 | SCN11A |  |  |  | |  |  |
| C3 | CRTAC1 |  |  |  | |  |  |
| C3 | GLRX |  |  |  | |  |  |
| C3 | ANXA10 |  |  |  | |  |  |
| C3 | CYP24A1 |  |  |  | |  |  |
| C3 | CTSE |  |  |  | |  |  |
| C3 | BST2 |  |  |  | |  |  |
| C3 | ZC3H12A |  |  |  | |  |  |
| C3 | TIMP3 |  |  |  | |  |  |
| C3 | KYNU |  |  |  | |  |  |
| C3 | IGFBP7 |  |  |  | |  |  |
| C3 | NSG1 |  |  |  | |  |  |
| C3 | TMEM92 |  |  |  | |  |  |
| C3 | AKR1C3 |  |  |  | |  |  |
| C3 | DNAJC15 |  |  |  | |  |  |
| C3 | TSC22D1 |  |  |  | |  |  |
| C3 | NFKBIA |  |  |  | |  |  |
| C3 | PLTP |  |  |  | |  |  |
| C3 | IGFBP5 |  |  |  | |  |  |
| C3 | TESC |  |  |  | |  |  |
| C3 | TALDO1 |  |  |  | |  |  |
| C3 | GSTO1 |  |  |  | |  |  |
| C3 | SRGN |  |  |  | |  |  |
| C3 | BMP2 |  |  |  | |  |  |
| C3 | ONECUT2 |  |  |  | |  |  |
| C3 | LEAP2 |  |  |  | |  |  |
| C3 | LGALS9 |  |  |  | |  |  |
| C3 | RAB34 |  |  |  | |  |  |
| C3 | PDLIM1 |  |  |  | |  |  |
| C3 | BTBD16 |  |  |  | |  |  |
| C3 | ARG2 |  |  |  | |  |  |
| C3 | HS6ST3 |  |  |  | |  |  |
| C3 | CEBPB |  |  |  | |  |  |
| C3 | SLC3A2 |  |  |  | |  |  |
| C3 | ID3 |  |  |  | |  |  |
| C3 | SRD5A2 |  |  |  | |  |  |
| C3 | ADH1C |  |  |  | |  |  |
| C3 | FCGRT |  |  |  | |  |  |
| C3 | HOXB2 |  |  |  | |  |  |
| C3 | ERRFI1 |  |  |  | |  |  |
| C3 | ODC1 |  |  |  | |  |  |
| C3 | CBR1 |  |  |  | |  |  |
| C3 | TMEM47 |  |  |  | |  |  |
| C3 | RNF39 |  |  |  | |  |  |
| C3 | NPM3 |  |  |  | |  |  |
| C3 | EFNB1 |  |  |  | |  |  |
| C3 | UTRN |  |  |  | |  |  |
| C3 | NDUFS4 |  |  |  | |  |  |
| C3 | AIM2 |  |  |  | |  |  |
| C3 | CYP1B1 |  |  |  | |  |  |
| C3 | ANKRD37 |  |  |  | |  |  |
| C3 | VSIG2 |  |  |  | |  |  |
| C3 | ARHGDIB |  |  |  | |  |  |
| C3 | CDC42EP4 |  |  |  | |  |  |
| C3 | RHOBTB3 |  |  |  | |  |  |
| C3 | HSPA8 |  |  |  | |  |  |
| C3 | PLAU |  |  |  | |  |  |
| C3 | SULT1E1 |  |  |  | |  |  |
| C3 | CYP4F11 |  |  |  | |  |  |
| C3 | BAG3 |  |  |  | |  |  |
| C3 | BTG2 |  |  |  | |  |  |
| C3 | TAF9 |  |  |  | |  |  |
| C3 | GSTO2 |  |  |  | |  |  |
| C3 | MBIP |  |  |  | |  |  |
| C3 | FABP6 |  |  |  | |  |  |
| C3 | TRAF4 |  |  |  | |  |  |
| C3 | GNPDA1 |  |  |  | |  |  |
| C3 | HOXB6 |  |  |  | |  |  |
| C3 | INSIG1 |  |  |  | |  |  |
| C3 | DLX5 |  |  |  | |  |  |
| C3 | CA12 |  |  |  | |  |  |
| C3 | CITED4 |  |  |  | |  |  |
| C3 | CYBA |  |  |  | |  |  |
| C3 | TBXAS1 |  |  |  | |  |  |
| C3 | BPGM |  |  |  | |  |  |
| C3 | BIRC3 |  |  |  | |  |  |
| C3 | EGFL6 |  |  |  | |  |  |
| C3 | GMPR2 |  |  |  | |  |  |
| C3 | RASD1 |  |  |  | |  |  |
| C3 | AVPI1 |  |  |  | |  |  |
| C3 | ZNF331 |  |  |  | |  |  |
| C3 | FTL |  |  |  | |  |  |
| C3 | CYP4B1 |  |  |  | |  |  |
| C3 | MRPL18 |  |  |  | |  |  |
| C3 | FAIM |  |  |  | |  |  |
| C3 | PSMB1 |  |  |  | |  |  |
| C3 | TNFRSF21 |  |  |  | |  |  |
| C3 | HIGD2A |  |  |  | |  |  |
| C3 | CHMP2B |  |  |  | |  |  |
| C3 | THYN1 |  |  |  | |  |  |
| C3 | EEF1A1 |  |  |  | |  |  |
| C3 | SLIT3 |  |  |  | |  |  |
| C3 | MGMT |  |  |  | |  |  |
| C3 | FBP1 |  |  |  | |  |  |
| C3 | RAB32 |  |  |  | |  |  |
| C3 | PSME2 |  |  |  | |  |  |
| C3 | DECR1 |  |  |  | |  |  |
| C3 | VDAC2 |  |  |  | |  |  |
| C3 | CCND1 |  |  |  | |  |  |
| C3 | TPGS2 |  |  |  | |  |  |
| C3 | ETS2 |  |  |  | |  |  |
| C3 | PKM |  |  |  | |  |  |
| C3 | PGAM1 |  |  |  | |  |  |
| C3 | CASP1 |  |  |  | |  |  |
| C3 | DDX5 |  |  |  | |  |  |
| C3 | DAD1 |  |  |  | |  |  |
| C3 | MAFF |  |  |  | |  |  |
| C3 | DNPH1 |  |  |  | |  |  |
| C3 | MPC2 |  |  |  | |  |  |
| C3 | EEF2 |  |  |  | |  |  |
| C3 | CEBPD |  |  |  | |  |  |
| C3 | APEX1 |  |  |  | |  |  |
| C3 | BCAS2 |  |  |  | |  |  |
| C3 | UGT2B7 |  |  |  | |  |  |
| C3 | HOXA5 |  |  |  | |  |  |
| C3 | AP2M1 |  |  |  | |  |  |
| C3 | AGR2 |  |  |  | |  |  |
| C3 | HNRNPA0 |  |  |  | |  |  |
| C3 | FABP5 |  |  |  | |  |  |
| C3 | DUSP5 |  |  |  | |  |  |
| C3 | PPA2 |  |  |  | |  |  |
| C3 | RPS13 |  |  |  | |  |  |
| C3 | CDKN1A |  |  |  | |  |  |
| C3 | TSEN34 |  |  |  | |  |  |
| C3 | GLRX3 |  |  |  | |  |  |
| C3 | RDX |  |  |  | |  |  |
| C3 | LRRC61 |  |  |  | |  |  |
| C3 | TXNL1 |  |  |  | |  |  |
| C3 | SNRPD2 |  |  |  | |  |  |
| C3 | CLDN1 |  |  |  | |  |  |
| C3 | ARID5B |  |  |  | |  |  |
| C3 | ID1 |  |  |  | |  |  |
| C3 | SRSF7 |  |  |  | |  |  |
| C3 | UNC5B |  |  |  | |  |  |
| C3 | ZSCAN18 |  |  |  | |  |  |
| C3 | PCBD1 |  |  |  | |  |  |
| C3 | COX5A |  |  |  | |  |  |
| C3 | PSMB3 |  |  |  | |  |  |
| C3 | HSPB1 |  |  |  | |  |  |
| C3 | ANXA4 |  |  |  | |  |  |
| C3 | RPL7 |  |  |  | |  |  |
| C3 | CXCL8 |  |  |  | |  |  |
| C3 | NAA20 |  |  |  | |  |  |
| C3 | NPC2 |  |  |  | |  |  |
| C3 | CASP4 |  |  |  | |  |  |
| C3 | POLR2J |  |  |  | |  |  |
| C3 | MAP2K3 |  |  |  | |  |  |
| C3 | PSMD8 |  |  |  | |  |  |
| C3 | CUTA |  |  |  | |  |  |
| C3 | PSMC5 |  |  |  | |  |  |
| C3 | TMEM97 |  |  |  | |  |  |
| C3 | CA2 |  |  |  | |  |  |
| C3 | SDC2 |  |  |  | |  |  |
| C3 | ADK |  |  |  | |  |  |
| C3 | PSMB5 |  |  |  | |  |  |
| C3 | CISD1 |  |  |  | |  |  |
| C3 | PHLDA2 |  |  |  | |  |  |
| C3 | TPT1 |  |  |  | |  |  |
| C3 | JUNB |  |  |  | |  |  |
| C3 | CSTB |  |  |  | |  |  |
| C3 | CHCHD2 |  |  |  | |  |  |
| C3 | RPL9 |  |  |  | |  |  |
| C3 | SDF2 |  |  |  | |  |  |
| C3 | PDCD2 |  |  |  | |  |  |
| C3 | PIFO |  |  |  | |  |  |
| C3 | ARL3 |  |  |  | |  |  |
| C3 | TRIP6 |  |  |  | |  |  |
| C3 | NFU1 |  |  |  | |  |  |
| C3 | ALDH3A1 |  |  |  | |  |  |
| C3 | COPE |  |  |  | |  |  |
| C3 | SRP14 |  |  |  | |  |  |
| C3 | TIMM9 |  |  |  | |  |  |
| C3 | LY6K |  |  |  | |  |  |
| C3 | RPL8 |  |  |  | |  |  |
| C3 | PSMA1 |  |  |  | |  |  |
| C3 | DPY30 |  |  |  | |  |  |
| C3 | ATP1B3 |  |  |  | |  |  |
| C3 | TPI1 |  |  |  | |  |  |
| C3 | PSMA3 |  |  |  | |  |  |
| C3 | RASIP1 |  |  |  | |  |  |
| C3 | RPL17 |  |  |  | |  |  |
| C3 | BAX |  |  |  | |  |  |
| C3 | ZNF503 |  |  |  | |  |  |
| C3 | MIEN1 |  |  |  | |  |  |
| C3 | ACTG1 |  |  |  | |  |  |
| C3 | NDUFB8 |  |  |  | |  |  |
| C3 | RPL19 |  |  |  | |  |  |
| C3 | HTATIP2 |  |  |  | |  |  |
| C3 | ZNF428 |  |  |  | |  |  |
| C3 | GAPDH |  |  |  | |  |  |
| C3 | FDX1 |  |  |  | |  |  |
| C3 | MYL6 |  |  |  | |  |  |
| C3 | RPL27 |  |  |  | |  |  |
| C3 | ZFP36L1 |  |  |  | |  |  |
| C3 | CP |  |  |  | |  |  |
| C3 | WFDC2 |  |  |  | |  |  |
| C3 | BRK1 |  |  |  | |  |  |
| C3 | BDH2 |  |  |  | |  |  |
| C3 | RPL23A |  |  |  | |  |  |
| C3 | PSMB8 |  |  |  | |  |  |
| C3 | HMGN3 |  |  |  | |  |  |
| C3 | PEPD |  |  |  | |  |  |
| C3 | QPCT |  |  |  | |  |  |
| C3 | IGBP1 |  |  |  | |  |  |
| C3 | MRPL32 |  |  |  | |  |  |
| C3 | ARPC2 |  |  |  | |  |  |
| C3 | EDN1 |  |  |  | |  |  |
| C3 | RPS25 |  |  |  | |  |  |
| C3 | IDI1 |  |  |  | |  |  |
| C3 | ADH5 |  |  |  | |  |  |
| C3 | ANXA7 |  |  |  | |  |  |
| C3 | SEL1L3 |  |  |  | |  |  |
| C3 | LITAF |  |  |  | |  |  |
| C3 | SEC61B |  |  |  | |  |  |
| C3 | NUTF2 |  |  |  | |  |  |
| C3 | RPL3 |  |  |  | |  |  |
| C3 | ZFP36 |  |  |  | |  |  |
| C3 | ARPC3 |  |  |  | |  |  |
| C3 | RPS10 |  |  |  | |  |  |
| C3 | BTG3 |  |  |  | |  |  |
| C3 | PTOV1 |  |  |  | |  |  |
| C3 | MRPL43 |  |  |  | |  |  |
| C3 | RPL10A |  |  |  | |  |  |
| C3 | KRT7 |  |  |  | |  |  |
| C3 | SAT1 |  |  |  | |  |  |
| C3 | GTF3A |  |  |  | |  |  |
| C3 | CFAP36 |  |  |  | |  |  |
| C3 | SAP18 |  |  |  | |  |  |
| C3 | HNRNPA1 |  |  |  | |  |  |
| C3 | MAGOH |  |  |  | |  |  |
| C3 | MTX2 |  |  |  | |  |  |
| C3 | RPS16 |  |  |  | |  |  |
| C3 | ATOH8 |  |  |  | |  |  |
| C3 | CDC26 |  |  |  | |  |  |
| C3 | AMZ2 |  |  |  | |  |  |
| C3 | XRCC5 |  |  |  | |  |  |
| C3 | UBE2D2 |  |  |  | |  |  |
| C3 | KRT19 |  |  |  | |  |  |
| C3 | HSPE1 |  |  |  | |  |  |
| C3 | ASS1 |  |  |  | |  |  |
| C3 | IL1A |  |  |  | |  |  |
| C3 | RPS2 |  |  |  | |  |  |
| C3 | UBXN1 |  |  |  | |  |  |
| C3 | BMP3 |  |  |  | |  |  |
| C3 | SLC25A4 |  |  |  | |  |  |
| C3 | FTH1 |  |  |  | |  |  |
| C3 | PSMC2 |  |  |  | |  |  |
| C3 | RPL31 |  |  |  | |  |  |
| C3 | NDUFB5 |  |  |  | |  |  |
| C3 | EPHA2 |  |  |  | |  |  |
| C3 | RPS4X |  |  |  | |  |  |
| C3 | DYNLT1 |  |  |  | |  |  |
| C3 | GPX2 |  |  |  | |  |  |
| C3 | CNBP |  |  |  | |  |  |
| C3 | NAPA |  |  |  | |  |  |
| C3 | GADD45B |  |  |  | |  |  |
| C3 | MAP1LC3B |  |  |  | |  |  |
| C3 | RPS5 |  |  |  | |  |  |
| C3 | GADD45A |  |  |  | |  |  |
| C3 | SARAF |  |  |  | |  |  |
| C3 | TFPT |  |  |  | |  |  |
| C3 | POLDIP2 |  |  |  | |  |  |
| C3 | MDH2 |  |  |  | |  |  |
| C3 | CROT |  |  |  | |  |  |
| C3 | S100A13 |  |  |  | |  |  |
| C3 | CD63 |  |  |  | |  |  |
| C3 | RPL5 |  |  |  | |  |  |
| C3 | ATP6V1G1 |  |  |  | |  |  |
| C3 | PSMA5 |  |  |  | |  |  |
| C3 | PRDX1 |  |  |  | |  |  |
| C3 | MYL12B |  |  |  | |  |  |
| C3 | GRN |  |  |  | |  |  |
| C3 | PLCXD2 |  |  |  | |  |  |
| C3 | RPS14 |  |  |  | |  |  |
| C3 | MPZL2 |  |  |  | |  |  |
| C3 | RPL10 |  |  |  | |  |  |
| C3 | CARD16 |  |  |  | |  |  |
| C3 | CUEDC2 |  |  |  | |  |  |
| C3 | TMSB4X |  |  |  | |  |  |
| C3 | HIBADH |  |  |  | |  |  |
| C3 | TMEM37 |  |  |  | |  |  |
| C3 | TMEM14C |  |  |  | |  |  |
| C3 | DUSP14 |  |  |  | |  |  |
| C3 | LSM2 |  |  |  | |  |  |
| C3 | RPLP2 |  |  |  | |  |  |
| C3 | RPS12 |  |  |  | |  |  |
| C3 | RNF5 |  |  |  | |  |  |
| C3 | RPL18A |  |  |  | |  |  |
| C3 | KRT10 |  |  |  | |  |  |
| C3 | HOXB8 |  |  |  | |  |  |
| C3 | PNKD |  |  |  | |  |  |
| C3 | PTP4A1 |  |  |  | |  |  |
| C3 | PCDH7 |  |  |  | |  |  |
| C3 | RPL18 |  |  |  | |  |  |
| C3 | RPL12 |  |  |  | |  |  |
| C3 | RPLP1 |  |  |  | |  |  |
| C3 | EPSTI1 |  |  |  | |  |  |
| C3 | MSX2 |  |  |  | |  |  |
| C3 | ATP6V0E1 |  |  |  | |  |  |
| C3 | EMC7 |  |  |  | |  |  |
| C3 | CLIC1 |  |  |  | |  |  |
| C3 | UXT |  |  |  | |  |  |
| C3 | MLF2 |  |  |  | |  |  |
| C3 | FIS1 |  |  |  | |  |  |
| C3 | TNFAIP3 |  |  |  | |  |  |
| C3 | MRPL13 |  |  |  | |  |  |
| C3 | FUCA2 |  |  |  | |  |  |
| C3 | DUSP1 |  |  |  | |  |  |
| C3 | PSMB7 |  |  |  | |  |  |
| C3 | RFXANK |  |  |  | |  |  |
| C3 | FOXJ1 |  |  |  | |  |  |
| C3 | FLNA |  |  |  | |  |  |
| C3 | RPS27A |  |  |  | |  |  |
| C3 | RPL34 |  |  |  | |  |  |
| C3 | MRPL54 |  |  |  | |  |  |
| C3 | ADM |  |  |  | |  |  |
| C3 | EDF1 |  |  |  | |  |  |
| C3 | SLC25A5 |  |  |  | |  |  |
| C3 | PYCARD |  |  |  | |  |  |
| C3 | TXN |  |  |  | |  |  |
| C3 | NOB1 |  |  |  | |  |  |
| C3 | UQCRC2 |  |  |  | |  |  |
| C3 | CYC1 |  |  |  | |  |  |
| C3 | CCNI |  |  |  | |  |  |
| C3 | ATXN10 |  |  |  | |  |  |
| C3 | MRPL11 |  |  |  | |  |  |
| C3 | GPX4 |  |  |  | |  |  |
| C3 | ATF3 |  |  |  | |  |  |
| C3 | G6PD |  |  |  | |  |  |
| C3 | KIAA0040 |  |  |  | |  |  |
| C3 | PSCA |  |  |  | |  |  |
| C3 | HSPA1B |  |  |  | |  |  |
| C3 | COMMD7 |  |  |  | |  |  |
| C3 | KLF5 |  |  |  | |  |  |
| C3 | OVOL1 |  |  |  | |  |  |
| C3 | PLBD1 |  |  |  | |  |  |
| C3 | REXO2 |  |  |  | |  |  |
| C3 | KRT8 |  |  |  | |  |  |
| C3 | RPL11 |  |  |  | |  |  |
| C3 | FOSL1 |  |  |  | |  |  |
| C3 | CCL20 |  |  |  | |  |  |
| C3 | ATRAID |  |  |  | |  |  |
| C3 | CCT6A |  |  |  | |  |  |
| C3 | FBLN1 |  |  |  | |  |  |
| C3 | CHID1 |  |  |  | |  |  |
| C3 | ZNF511 |  |  |  | |  |  |
| C3 | NFE2L2 |  |  |  | |  |  |
| C3 | PPP2R2B |  |  |  | |  |  |
| C3 | MFF |  |  |  | |  |  |
| C3 | SPINT2 |  |  |  | |  |  |
| C3 | BTG1 |  |  |  | |  |  |
| C3 | HSF1 |  |  |  | |  |  |
| C3 | FAU |  |  |  | |  |  |
| C3 | ACAT2 |  |  |  | |  |  |
| C3 | IFRD1 |  |  |  | |  |  |
| C3 | EIF4G2 |  |  |  | |  |  |
| C3 | TCEAL1 |  |  |  | |  |  |
| C3 | UCHL3 |  |  |  | |  |  |
| C3 | JUP |  |  |  | |  |  |
| C3 | MAF1 |  |  |  | |  |  |
| C3 | CLDN4 |  |  |  | |  |  |
| C3 | RBPMS |  |  |  | |  |  |
| C3 | TIMM8B |  |  |  | |  |  |
| C3 | EHF |  |  |  | |  |  |
| C3 | PSMF1 |  |  |  | |  |  |
| C3 | ILF2 |  |  |  | |  |  |
| C3 | RPL36AL |  |  |  | |  |  |
| C3 | LDHB |  |  |  | |  |  |
| C3 | MPC1 |  |  |  | |  |  |
| C3 | TMEM243 |  |  |  | |  |  |
| C3 | RGS16 |  |  |  | |  |  |
| C3 | FAM3C |  |  |  | |  |  |
| C3 | DUSP6 |  |  |  | |  |  |
| C3 | NDUFAB1 |  |  |  | |  |  |
| C3 | RAB9A |  |  |  | |  |  |
| C3 | NDUFS5 |  |  |  | |  |  |
| C3 | PEBP1 |  |  |  | |  |  |
| C3 | SMDT1 |  |  |  | |  |  |
| C3 | PABPC1 |  |  |  | |  |  |
| C3 | TMEM219 |  |  |  | |  |  |
| C3 | HNRNPF |  |  |  | |  |  |
| C3 | EIF4A2 |  |  |  | |  |  |
| C3 | SNRPC |  |  |  | |  |  |
| C3 | SERPINE1 |  |  |  | |  |  |
| C3 | S100P |  |  |  | |  |  |
| C3 | RPL22 |  |  |  | |  |  |
| C3 | EID1 |  |  |  | |  |  |
| C3 | FUNDC1 |  |  |  | |  |  |
| C3 | MEST |  |  |  | |  |  |
| C3 | HEXB |  |  |  | |  |  |
| C3 | UQCRFS1 |  |  |  | |  |  |
| C3 | COMMD1 |  |  |  | |  |  |
| C3 | CSRNP1 |  |  |  | |  |  |
| C3 | CSTA |  |  |  | |  |  |
| C3 | PTS |  |  |  | |  |  |
| C3 | NDUFB3 |  |  |  | |  |  |
| C3 | NET1 |  |  |  | |  |  |
| C3 | ATP6V1F |  |  |  | |  |  |
| C3 | COMMD6 |  |  |  | |  |  |
| C3 | ELL2 |  |  |  | |  |  |
| C3 | PRDX5 |  |  |  | |  |  |
| C3 | ADRB2 |  |  |  | |  |  |
| C3 | RHOB |  |  |  | |  |  |
| C3 | SNRPE |  |  |  | |  |  |
| C3 | ARL4D |  |  |  | |  |  |
| C3 | MFSD2A |  |  |  | |  |  |
| C3 | NRBF2 |  |  |  | |  |  |
| C3 | ADIRF |  |  |  | |  |  |
| C3 | NFKBIE |  |  |  | |  |  |
| C3 | OSER1 |  |  |  | |  |  |
| C3 | IFI6 |  |  |  | |  |  |
| C3 | SNX3 |  |  |  | |  |  |
| C3 | ERH |  |  |  | |  |  |
| C3 | HSP90AB1 |  |  |  | |  |  |
| C3 | SRSF2 |  |  |  | |  |  |
| C3 | MRPS36 |  |  |  | |  |  |
| C3 | PRDX4 |  |  |  | |  |  |
| C3 | REEP5 |  |  |  | |  |  |
| C3 | TOB2 |  |  |  | |  |  |
| C3 | DNAJB4 |  |  |  | |  |  |
| C3 | SNRPB |  |  |  | |  |  |
| C3 | NXT1 |  |  |  | |  |  |
| C3 | TUFM |  |  |  | |  |  |
| C3 | BLVRB |  |  |  | |  |  |
| C3 | DYNLRB1 |  |  |  | |  |  |
| C3 | IGFBP3 |  |  |  | |  |  |
| C3 | SMIM19 |  |  |  | |  |  |
| C3 | RPL37A |  |  |  | |  |  |
| C3 | PSMB9 |  |  |  | |  |  |
| C3 | MRPL51 |  |  |  | |  |  |
| C3 | IFT57 |  |  |  | |  |  |
| C3 | C12orf57 |  |  |  | |  |  |
| C3 | CNN3 |  |  |  | |  |  |
| C3 | SERP1 |  |  |  | |  |  |
| C3 | SSR4 |  |  |  | |  |  |
| C3 | MYL12A |  |  |  | |  |  |
| C3 | IFI16 |  |  |  | |  |  |
| C3 | DEDD2 |  |  |  | |  |  |
| C3 | THEM6 |  |  |  | |  |  |
| C3 | EPCAM |  |  |  | |  |  |
| C3 | GMDS |  |  |  | |  |  |
| C3 | MRPL22 |  |  |  | |  |  |
| C3 | RNF114 |  |  |  | |  |  |
| C3 | NFIL3 |  |  |  | |  |  |
| C3 | GBP2 |  |  |  | |  |  |
| C3 | ILK |  |  |  | |  |  |
| C3 | XRCC6 |  |  |  | |  |  |
| C3 | NSMCE1 |  |  |  | |  |  |
| C3 | SH3BGRL |  |  |  | |  |  |
| C3 | MGST1 |  |  |  | |  |  |
| C3 | CYP3A5 |  |  |  | |  |  |
| C3 | TSPO |  |  |  | |  |  |
| C3 | HOXD1 |  |  |  | |  |  |
| C3 | SLC35B1 |  |  |  | |  |  |
| C3 | PUF60 |  |  |  | |  |  |
| C3 | TMEM60 |  |  |  | |  |  |
| C3 | PLSCR1 |  |  |  | |  |  |
| C3 | PSMG2 |  |  |  | |  |  |
| C3 | IL23A |  |  |  | |  |  |
| C3 | BNIP3L |  |  |  | |  |  |
| C3 | PFN2 |  |  |  | |  |  |
| C3 | ADRM1 |  |  |  | |  |  |
| C3 | ZFAND2A |  |  |  | |  |  |
| C3 | TRAPPC6A |  |  |  | |  |  |
| C3 | CACYBP |  |  |  | |  |  |
| C3 | ARPC1A |  |  |  | |  |  |
| C3 | MRPL15 |  |  |  | |  |  |
| C3 | TSPAN13 |  |  |  | |  |  |
| C3 | BABAM1 |  |  |  | |  |  |
| C3 | NDUFA8 |  |  |  | |  |  |
| C3 | BLVRA |  |  |  | |  |  |
| C3 | RTP4 |  |  |  | |  |  |
| C3 | BIK |  |  |  | |  |  |
| C3 | S100A11 |  |  |  | |  |  |
| C3 | NDUFA13 |  |  |  | |  |  |
| C3 | SF3B6 |  |  |  | |  |  |
| C3 | RBCK1 |  |  |  | |  |  |
| C3 | HDDC2 |  |  |  | |  |  |
| C3 | PRPF31 |  |  |  | |  |  |
| C3 | RPL41 |  |  |  | |  |  |
| C3 | ECH1 |  |  |  | |  |  |
| C3 | SF3B5 |  |  |  | |  |  |
| C3 | EDN2 |  |  |  | |  |  |
| C3 | AP1M2 |  |  |  | |  |  |
| C3 | COX7C |  |  |  | |  |  |
| C3 | NDFIP1 |  |  |  | |  |  |
| C3 | LYPLAL1 |  |  |  | |  |  |
| C3 | BBX |  |  |  | |  |  |
| C3 | GPX1 |  |  |  | |  |  |
| C3 | PROC |  |  |  | |  |  |
| C3 | HIBCH |  |  |  | |  |  |
| C3 | TPCN2 |  |  |  | |  |  |
| C3 | CAT |  |  |  | |  |  |
| C3 | IGFBP4 |  |  |  | |  |  |
| C3 | SNRPG |  |  |  | |  |  |
| C3 | ZCRB1 |  |  |  | |  |  |
| C3 | NDUFC1 |  |  |  | |  |  |
| C3 | FA2H |  |  |  | |  |  |
| C3 | MPV17 |  |  |  | |  |  |
| C3 | RAC1 |  |  |  | |  |  |
| C3 | BAMBI |  |  |  | |  |  |
| C3 | UQCC2 |  |  |  | |  |  |
| C3 | KLHDC2 |  |  |  | |  |  |
| C3 | HNRNPH3 |  |  |  | |  |  |
| C3 | TMEM18 |  |  |  | |  |  |
| C3 | COMMD3 |  |  |  | |  |  |
| C3 | LAMTOR4 |  |  |  | |  |  |
| C3 | RPS28 |  |  |  | |  |  |
| C3 | NDUFB11 |  |  |  | |  |  |
| C3 | TMEM120A |  |  |  | |  |  |
| C3 | BAZ1A |  |  |  | |  |  |
| C3 | GNG5 |  |  |  | |  |  |
| C3 | BCL2L12 |  |  |  | |  |  |
| C3 | SF3B4 |  |  |  | |  |  |
| C3 | RPL39 |  |  |  | |  |  |
| C3 | FRA10AC1 |  |  |  | |  |  |
| C3 | GATA3 |  |  |  | |  |  |
| C3 | HNRNPK |  |  |  | |  |  |
| C3 | MYL9 |  |  |  | |  |  |
| C3 | UBC |  |  |  | |  |  |
| C3 | CMC2 |  |  |  | |  |  |
| C3 | SLC25A39 |  |  |  | |  |  |
| C3 | LY96 |  |  |  | |  |  |
| C3 | NT5DC1 |  |  |  | |  |  |
| C3 | CHCHD3 |  |  |  | |  |  |
| C3 | AIFM2 |  |  |  | |  |  |
| C3 | LMAN2 |  |  |  | |  |  |
| C3 | WDR83OS |  |  |  | |  |  |
| C3 | MRPL27 |  |  |  | |  |  |
| C3 | ARHGAP6 |  |  |  | |  |  |
| C3 | TIMM10 |  |  |  | |  |  |
| C3 | NANS |  |  |  | |  |  |
| C3 | CNPY2 |  |  |  | |  |  |
| C3 | ISCU |  |  |  | |  |  |
| C3 | UBE2L6 |  |  |  | |  |  |
| C3 | PFN1 |  |  |  | |  |  |
| C3 | RAB7A |  |  |  | |  |  |
| C3 | ITM2B |  |  |  | |  |  |
| C3 | RIPK4 |  |  |  | |  |  |
| C3 | RPS27 |  |  |  | |  |  |
| C3 | AAMP |  |  |  | |  |  |
| C3 | BCL3 |  |  |  | |  |  |
| C3 | PRDX6 |  |  |  | |  |  |
| C3 | VASP |  |  |  | |  |  |
| C3 | ZNF580 |  |  |  | |  |  |
| C3 | TGFB1 |  |  |  | |  |  |
| C3 | SHKBP1 |  |  |  | |  |  |
| C3 | EI24 |  |  |  | |  |  |
| C3 | SRSF3 |  |  |  | |  |  |
| C3 | CDC42EP5 |  |  |  | |  |  |
| C4 | CFD |  |  |  | |  |  |
| C4 | EMP1 |  |  |  | |  |  |
| C4 | FABP4 |  |  |  | |  |  |
| C4 | PPP1R1B |  |  |  | |  |  |
| C4 | IGFBP6 |  |  |  | |  |  |
| C4 | AGR3 |  |  |  | |  |  |
| C4 | ZDHHC2 |  |  |  | |  |  |
| C4 | PRAC1 |  |  |  | |  |  |
| C4 | OLFM4 |  |  |  | |  |  |
| C4 | SOX15 |  |  |  | |  |  |
| C4 | SPOCK1 |  |  |  | |  |  |
| C4 | MTUS2 |  |  |  | |  |  |
| C4 | RIC3 |  |  |  | |  |  |
| C4 | VAMP5 |  |  |  | |  |  |
| C4 | ANXA1 |  |  |  | |  |  |
| C4 | CYSTM1 |  |  |  | |  |  |
| C4 | MSMB |  |  |  | |  |  |
| C4 | TSPAN8 |  |  |  | |  |  |
| C4 | ATF3 |  |  |  | |  |  |
| C4 | RGCC |  |  |  | |  |  |
| C4 | CCND2 |  |  |  | |  |  |
| C4 | LDHB |  |  |  | |  |  |
| C4 | EGR1 |  |  |  | |  |  |
| C4 | MEF2C |  |  |  | |  |  |
| C4 | SHH |  |  |  | |  |  |
| C4 | CFH |  |  |  | |  |  |
| C4 | LPCAT4 |  |  |  | |  |  |
| C4 | CCDC60 |  |  |  | |  |  |
| C4 | ZFP36 |  |  |  | |  |  |
| C4 | ANG |  |  |  | |  |  |
| C4 | TRIM31 |  |  |  | |  |  |
| C4 | FBP1 |  |  |  | |  |  |
| C4 | PLA2G10 |  |  |  | |  |  |
| C4 | ISCU |  |  |  | |  |  |
| C4 | AQP3 |  |  |  | |  |  |
| C4 | KRT13 |  |  |  | |  |  |
| C4 | SLC16A4 |  |  |  | |  |  |
| C4 | TGFBI |  |  |  | |  |  |
| C4 | ITM2C |  |  |  | |  |  |
| C4 | FOSB |  |  |  | |  |  |
| C4 | MDFI |  |  |  | |  |  |
| C4 | GABARAPL1 |  |  |  | |  |  |
| C4 | SIGLEC15 |  |  |  | |  |  |
| C4 | ASCL2 |  |  |  | |  |  |
| C4 | EPS8 |  |  |  | |  |  |
| C4 | DUSP1 |  |  |  | |  |  |
| C4 | ESD |  |  |  | |  |  |
| C4 | JUNB |  |  |  | |  |  |
| C4 | PDLIM1 |  |  |  | |  |  |
| C4 | UPK3B |  |  |  | |  |  |
| C4 | BTBD16 |  |  |  | |  |  |
| C4 | IL18 |  |  |  | |  |  |
| C4 | LAMB3 |  |  |  | |  |  |
| C4 | SLITRK6 |  |  |  | |  |  |
| C4 | MAP3K5 |  |  |  | |  |  |
| C4 | MGST2 |  |  |  | |  |  |
| C4 | LMO7 |  |  |  | |  |  |
| C4 | SPINK4 |  |  |  | |  |  |
| C4 | LGALS3 |  |  |  | |  |  |
| C4 | RSL24D1 |  |  |  | |  |  |
| C4 | EIF4A2 |  |  |  | |  |  |
| C4 | CD44 |  |  |  | |  |  |
| C4 | GAMT |  |  |  | |  |  |
| C4 | TOB1 |  |  |  | |  |  |
| C4 | MAGED2 |  |  |  | |  |  |
| C4 | SERTAD3 |  |  |  | |  |  |
| C4 | UBC |  |  |  | |  |  |
| C4 | EGR3 |  |  |  | |  |  |
| C4 | FOS |  |  |  | |  |  |
| C4 | MPZL2 |  |  |  | |  |  |
| C4 | CLIC6 |  |  |  | |  |  |
| C4 | CCDC28A |  |  |  | |  |  |
| C4 | LPAR6 |  |  |  | |  |  |
| C4 | ST6GALNAC2 |  |  |  | |  |  |
| C4 | SNCG |  |  |  | |  |  |
| C4 | PLAUR |  |  |  | |  |  |
| C4 | NFIB |  |  |  | |  |  |
| C4 | APOD |  |  |  | |  |  |
| C4 | CIRBP |  |  |  | |  |  |
| C4 | DHRS2 |  |  |  | |  |  |
| C4 | TXNIP |  |  |  | |  |  |
| C4 | MAP3K6 |  |  |  | |  |  |
| C4 | CDKN1C |  |  |  | |  |  |
| C4 | TSPAN1 |  |  |  | |  |  |
| C4 | GADD45B |  |  |  | |  |  |
| C4 | COMMD6 |  |  |  | |  |  |
| C4 | DEGS2 |  |  |  | |  |  |
| C4 | DDX24 |  |  |  | |  |  |
| C4 | FRY |  |  |  | |  |  |
| C4 | PARP4 |  |  |  | |  |  |
| C4 | WNT10A |  |  |  | |  |  |
| C4 | HNRNPA1 |  |  |  | |  |  |
| C4 | CLDN4 |  |  |  | |  |  |
| C4 | CLEC2B |  |  |  | |  |  |
| C4 | FOSL1 |  |  |  | |  |  |
| C4 | AVPI1 |  |  |  | |  |  |
| C4 | ITM2B |  |  |  | |  |  |
| C4 | PERP |  |  |  | |  |  |
| C4 | PCBP2 |  |  |  | |  |  |
| C4 | TMBIM6 |  |  |  | |  |  |
| C4 | GSTP1 |  |  |  | |  |  |
| C4 | SARAF |  |  |  | |  |  |
| C4 | MED21 |  |  |  | |  |  |
| C4 | SMDT1 |  |  |  | |  |  |
| C4 | TMEM9B |  |  |  | |  |  |
| C4 | KIZ |  |  |  | |  |  |
| C4 | ASS1 |  |  |  | |  |  |
| C4 | TPT1 |  |  |  | |  |  |
| C4 | SH3BGRL |  |  |  | |  |  |
| C4 | CHMP5 |  |  |  | |  |  |
| C4 | GPX2 |  |  |  | |  |  |
| C4 | MMP2 |  |  |  | |  |  |
| C4 | ANO6 |  |  |  | |  |  |
| C4 | AGR2 |  |  |  | |  |  |
| C4 | SIPA1 |  |  |  | |  |  |
| C4 | MCL1 |  |  |  | |  |  |
| C4 | CAPG |  |  |  | |  |  |
| C4 | EEF1A1 |  |  |  | |  |  |
| C4 | MRPS31 |  |  |  | |  |  |
| C4 | GSTO1 |  |  |  | |  |  |
| C4 | JUN |  |  |  | |  |  |
| C4 | TRAPPC1 |  |  |  | |  |  |
| C4 | NDUFB8 |  |  |  | |  |  |
| C4 | SAP18 |  |  |  | |  |  |
| C4 | SPAG7 |  |  |  | |  |  |
| C4 | MIDN |  |  |  | |  |  |
| C4 | ELF1 |  |  |  | |  |  |
| C4 | BTG2 |  |  |  | |  |  |
| C4 | ZNF385A |  |  |  | |  |  |
| C4 | BEX4 |  |  |  | |  |  |
| C4 | NT5E |  |  |  | |  |  |
| C4 | MYOF |  |  |  | |  |  |
| C4 | MPPED2 |  |  |  | |  |  |
| C4 | GATA2 |  |  |  | |  |  |
| C4 | RPL36AL |  |  |  | |  |  |
| C4 | AEBP2 |  |  |  | |  |  |
| C4 | DGKA |  |  |  | |  |  |
| C4 | ZFAND1 |  |  |  | |  |  |
| C4 | RPL10A |  |  |  | |  |  |
| C4 | GJB3 |  |  |  | |  |  |
| C4 | ARPC3 |  |  |  | |  |  |
| C4 | TRIM29 |  |  |  | |  |  |
| C4 | SDCBP2 |  |  |  | |  |  |
| C4 | BCL7A |  |  |  | |  |  |
| C4 | RHOC |  |  |  | |  |  |
| C4 | KLF5 |  |  |  | |  |  |
| C4 | RPS25 |  |  |  | |  |  |
| C4 | VILL |  |  |  | |  |  |
| C4 | YPEL5 |  |  |  | |  |  |
| C4 | CARD16 |  |  |  | |  |  |
| C4 | TUBA1A |  |  |  | |  |  |
| C4 | PEBP1 |  |  |  | |  |  |
| C4 | PPP1R21 |  |  |  | |  |  |
| C4 | RPS13 |  |  |  | |  |  |
| C4 | RPL5 |  |  |  | |  |  |
| C4 | ZMYM2 |  |  |  | |  |  |
| C4 | RBPMS |  |  |  | |  |  |
| C4 | RTN4 |  |  |  | |  |  |
| C4 | RILPL2 |  |  |  | |  |  |
| C4 | MTA1 |  |  |  | |  |  |
| C4 | ACADVL |  |  |  | |  |  |
| C4 | ERGIC2 |  |  |  | |  |  |
| C4 | EDF1 |  |  |  | |  |  |
| C4 | DRAM2 |  |  |  | |  |  |
| C4 | TXNDC17 |  |  |  | |  |  |
| C4 | SLC40A1 |  |  |  | |  |  |
| C4 | UQCRC2 |  |  |  | |  |  |
| C4 | HIGD2A |  |  |  | |  |  |
| C4 | EIF4G2 |  |  |  | |  |  |
| C4 | RPL41 |  |  |  | |  |  |
| C4 | TACC1 |  |  |  | |  |  |
| C4 | DHRS3 |  |  |  | |  |  |
| C4 | MTIF3 |  |  |  | |  |  |
| C4 | ID1 |  |  |  | |  |  |
| C4 | PRDX5 |  |  |  | |  |  |
| C4 | NAA38 |  |  |  | |  |  |
| C4 | NDFIP1 |  |  |  | |  |  |
| C4 | CCNG1 |  |  |  | |  |  |
| C4 | DUSP4 |  |  |  | |  |  |
| C4 | TMEM256 |  |  |  | |  |  |
| C4 | EEF2 |  |  |  | |  |  |
| C4 | PKM |  |  |  | |  |  |
| C4 | CAT |  |  |  | |  |  |
| C4 | PRKCD |  |  |  | |  |  |
| C4 | VDAC2 |  |  |  | |  |  |
| C4 | RPL7 |  |  |  | |  |  |
| C4 | SNAI2 |  |  |  | |  |  |
| C4 | MYL6 |  |  |  | |  |  |
| C4 | MYL12B |  |  |  | |  |  |
| C4 | C1orf21 |  |  |  | |  |  |
| C4 | ASF1A |  |  |  | |  |  |
| C4 | ARHGAP6 |  |  |  | |  |  |
| C4 | JUND |  |  |  | |  |  |
| C4 | TRIB1 |  |  |  | |  |  |
| C4 | IGBP1 |  |  |  | |  |  |
| C4 | DDX5 |  |  |  | |  |  |
| C4 | RIOK3 |  |  |  | |  |  |
| C4 | COL9A2 |  |  |  | |  |  |
| C4 | EPHA2 |  |  |  | |  |  |
| C4 | KLHDC2 |  |  |  | |  |  |
| C4 | TSPO |  |  |  | |  |  |
| C4 | PLP2 |  |  |  | |  |  |
| C4 | CWF19L2 |  |  |  | |  |  |
| C4 | RPL34 |  |  |  | |  |  |
| C4 | METAP1 |  |  |  | |  |  |
| C4 | RPS16 |  |  |  | |  |  |
| C4 | TESC |  |  |  | |  |  |
| C4 | UPK1A |  |  |  | |  |  |
| C4 | PPP1R10 |  |  |  | |  |  |
| C4 | TM9SF2 |  |  |  | |  |  |
| C4 | RPS27A |  |  |  | |  |  |
| C4 | RPL17 |  |  |  | |  |  |
| C4 | RAPGEFL1 |  |  |  | |  |  |
| C4 | RPL31 |  |  |  | |  |  |
| C4 | RPS27 |  |  |  | |  |  |
| C4 | PABPC1 |  |  |  | |  |  |
| C4 | HNRNPA0 |  |  |  | |  |  |
| C4 | FABP5 |  |  |  | |  |  |
| C4 | DAAM1 |  |  |  | |  |  |
| C4 | COX6A1 |  |  |  | |  |  |
| C4 | RPS4X |  |  |  | |  |  |
| C4 | ZNF552 |  |  |  | |  |  |
| C4 | M6PR |  |  |  | |  |  |
| C4 | KCNN4 |  |  |  | |  |  |
| C4 | RPL12 |  |  |  | |  |  |
| C4 | AMIGO2 |  |  |  | |  |  |
| C4 | SPINT2 |  |  |  | |  |  |
| C4 | RPL18 |  |  |  | |  |  |
| C4 | LRP1 |  |  |  | |  |  |
| C4 | ISOC1 |  |  |  | |  |  |
| C4 | RPS4Y1 |  |  |  | |  |  |
| C4 | RPL9 |  |  |  | |  |  |
| C4 | THG1L |  |  |  | |  |  |
| C4 | ITPKC |  |  |  | |  |  |
| C4 | LITAF |  |  |  | |  |  |
| C4 | UCHL3 |  |  |  | |  |  |
| C4 | DUSP5 |  |  |  | |  |  |
| C4 | OVOL1 |  |  |  | |  |  |
| C4 | PTPRN2 |  |  |  | |  |  |
| C4 | PSMG2 |  |  |  | |  |  |
| C4 | PELI1 |  |  |  | |  |  |
| C4 | RPL3 |  |  |  | |  |  |
| C4 | XRCC5 |  |  |  | |  |  |
| C4 | SLPI |  |  |  | |  |  |
| C4 | RPS14 |  |  |  | |  |  |
| C4 | RPL23A |  |  |  | |  |  |
| C4 | ATP8B1 |  |  |  | |  |  |
| C4 | SATB1 |  |  |  | |  |  |
| C4 | HNRNPK |  |  |  | |  |  |
| C4 | BLOC1S1 |  |  |  | |  |  |
| C4 | SRP14 |  |  |  | |  |  |
| C4 | RPL8 |  |  |  | |  |  |
| C4 | RPLP2 |  |  |  | |  |  |
| C4 | HNRNPC |  |  |  | |  |  |
| C4 | RPS28 |  |  |  | |  |  |
| C4 | RPS5 |  |  |  | |  |  |
| C4 | PSCA |  |  |  | |  |  |
| C4 | SNRPD2 |  |  |  | |  |  |
| C4 | RPLP1 |  |  |  | |  |  |
| C4 | IRF6 |  |  |  | |  |  |
| C4 | NDUFS5 |  |  |  | |  |  |
| C4 | NDUFC1 |  |  |  | |  |  |
| C4 | FUCA1 |  |  |  | |  |  |
| C4 | PLEC |  |  |  | |  |  |
| C4 | DKK1 |  |  |  | |  |  |
| C4 | MAX |  |  |  | |  |  |
| C4 | ARGLU1 |  |  |  | |  |  |
| C4 | NFE2L2 |  |  |  | |  |  |
| C4 | ADIRF |  |  |  | |  |  |
| C4 | PFN1 |  |  |  | |  |  |
| C4 | MYL12A |  |  |  | |  |  |
| C4 | POMP |  |  |  | |  |  |
| C4 | LAPTM4A |  |  |  | |  |  |
| C4 | PDLIM4 |  |  |  | |  |  |
| C4 | EIF1B |  |  |  | |  |  |
| C4 | OAT |  |  |  | |  |  |
| C4 | ARL6IP4 |  |  |  | |  |  |
| C4 | NDUFA1 |  |  |  | |  |  |
| C4 | PTPN13 |  |  |  | |  |  |
| C4 | TWF1 |  |  |  | |  |  |
| C4 | CLIC1 |  |  |  | |  |  |
| C4 | RPL22 |  |  |  | |  |  |
| C4 | HOXB7 |  |  |  | |  |  |
| C4 | CD63 |  |  |  | |  |  |
| C4 | IRF2 |  |  |  | |  |  |
| C4 | TMA7 |  |  |  | |  |  |
| C4 | KLF10 |  |  |  | |  |  |
| C4 | ANXA7 |  |  |  | |  |  |
| C4 | ABLIM1 |  |  |  | |  |  |
| C4 | PTMA |  |  |  | |  |  |
| C4 | ARPC2 |  |  |  | |  |  |
| C4 | ODC1 |  |  |  | |  |  |
| C4 | GABARAPL2 |  |  |  | |  |  |
| C4 | DTNB |  |  |  | |  |  |
| C4 | LY6D |  |  |  | |  |  |
| C4 | ATP2B1 |  |  |  | |  |  |
| C4 | ZC3H13 |  |  |  | |  |  |
| C4 | IK |  |  |  | |  |  |
| C4 | ZMIZ1 |  |  |  | |  |  |
| C4 | ATF1 |  |  |  | |  |  |
| C4 | RPL11 |  |  |  | |  |  |
| C4 | HOXA5 |  |  |  | |  |  |
| C4 | LSM6 |  |  |  | |  |  |
| C4 | RNF145 |  |  |  | |  |  |
| C4 | VASP |  |  |  | |  |  |
| C4 | SYPL1 |  |  |  | |  |  |
| C4 | IGFBP5 |  |  |  | |  |  |
| C4 | RPL39 |  |  |  | |  |  |
| C4 | DCTN3 |  |  |  | |  |  |
| C4 | IQGAP1 |  |  |  | |  |  |
| C4 | RAB18 |  |  |  | |  |  |
| C4 | LAMA3 |  |  |  | |  |  |
| C4 | SLC35A1 |  |  |  | |  |  |
| C4 | AHNAK |  |  |  | |  |  |
| C4 | SEMA4A |  |  |  | |  |  |
| C4 | PNISR |  |  |  | |  |  |
| C4 | PIM1 |  |  |  | |  |  |
| C4 | ABHD13 |  |  |  | |  |  |
| C4 | NCOA4 |  |  |  | |  |  |
| C4 | GBP2 |  |  |  | |  |  |
| C4 | FAT1 |  |  |  | |  |  |
| C4 | TMBIM4 |  |  |  | |  |  |
| C4 | CHID1 |  |  |  | |  |  |
| C4 | RPS12 |  |  |  | |  |  |
| C4 | ETS2 |  |  |  | |  |  |
| C4 | LYPD3 |  |  |  | |  |  |
| C4 | UBXN1 |  |  |  | |  |  |
| C4 | USP11 |  |  |  | |  |  |
| C4 | KLF2 |  |  |  | |  |  |
| C4 | RPL37A |  |  |  | |  |  |
| C4 | TBC1D4 |  |  |  | |  |  |
| C4 | CCNI |  |  |  | |  |  |
| C4 | RPS2 |  |  |  | |  |  |
| C4 | CLSTN3 |  |  |  | |  |  |
| C4 | ANAPC4 |  |  |  | |  |  |
| C4 | RPL10 |  |  |  | |  |  |
| C4 | S100A11 |  |  |  | |  |  |
| C4 | PLCG2 |  |  |  | |  |  |
| C4 | ECH1 |  |  |  | |  |  |
| C4 | UNC5B |  |  |  | |  |  |
| C4 | S100P |  |  |  | |  |  |
| C4 | RRAGA |  |  |  | |  |  |
| C4 | FAU |  |  |  | |  |  |
| C4 | ZBTB44 |  |  |  | |  |  |
| C4 | HEBP1 |  |  |  | |  |  |
| C4 | TCEAL8 |  |  |  | |  |  |
| C4 | MRPL51 |  |  |  | |  |  |
| C4 | FLOT1 |  |  |  | |  |  |
| C4 | CLK1 |  |  |  | |  |  |
| C4 | ERH |  |  |  | |  |  |
| C4 | COX7C |  |  |  | |  |  |
| C4 | WBP11 |  |  |  | |  |  |
| C4 | PSAP |  |  |  | |  |  |
| C4 | WDR83OS |  |  |  | |  |  |
| C4 | SSNA1 |  |  |  | |  |  |
| C4 | TMEM245 |  |  |  | |  |  |
| C4 | RGP1 |  |  |  | |  |  |
| C4 | BNIP3L |  |  |  | |  |  |
| C4 | VSIG2 |  |  |  | |  |  |
| C4 | PPP2R2A |  |  |  | |  |  |
| C4 | BST2 |  |  |  | |  |  |
| C4 | CYBA |  |  |  | |  |  |
| C4 | CNFN |  |  |  | |  |  |
| C4 | GTF2F2 |  |  |  | |  |  |
| C4 | ACTG1 |  |  |  | |  |  |
| C4 | HMGN3 |  |  |  | |  |  |
| C4 | TUSC3 |  |  |  | |  |  |
| C4 | MBNL2 |  |  |  | |  |  |
| C4 | TP63 |  |  |  | |  |  |
| C4 | HES1 |  |  |  | |  |  |
| C4 | ARHGDIB |  |  |  | |  |  |
| C4 | C12orf57 |  |  |  | |  |  |
| C4 | ID3 |  |  |  | |  |  |
| C4 | RPL19 |  |  |  | |  |  |
| C4 | FTH1 |  |  |  | |  |  |
| C4 | CNOT7 |  |  |  | |  |  |
| C4 | CNBP |  |  |  | |  |  |
| C4 | WBP4 |  |  |  | |  |  |
| C4 | TMEM219 |  |  |  | |  |  |
| C4 | NFKBIA |  |  |  | |  |  |
| C4 | CMYA5 |  |  |  | |  |  |
| C4 | SNRPE |  |  |  | |  |  |
| C4 | HDAC1 |  |  |  | |  |  |
| C4 | HNRNPH2 |  |  |  | |  |  |
| C4 | PSMB1 |  |  |  | |  |  |
| C4 | TAX1BP1 |  |  |  | |  |  |
| C4 | TANK |  |  |  | |  |  |
| C4 | RILP |  |  |  | |  |  |
| C4 | RPL18A |  |  |  | |  |  |
| C4 | MAOA |  |  |  | |  |  |
| C4 | BRK1 |  |  |  | |  |  |
| C4 | KRT19 |  |  |  | |  |  |
| C4 | MRPS36 |  |  |  | |  |  |
| C4 | GABARAP |  |  |  | |  |  |
| C4 | ATAD1 |  |  |  | |  |  |
| C4 | NDUFAF3 |  |  |  | |  |  |
| C4 | CEP131 |  |  |  | |  |  |
| C4 | CD58 |  |  |  | |  |  |
| C4 | METAP2 |  |  |  | |  |  |
| C4 | ZC2HC1A |  |  |  | |  |  |
| C4 | KLF6 |  |  |  | |  |  |
| C4 | NDUFS4 |  |  |  | |  |  |
| C4 | HNRNPDL |  |  |  | |  |  |
| C4 | SPRYD3 |  |  |  | |  |  |
| C4 | HMGN1 |  |  |  | |  |  |
| C4 | CASP4 |  |  |  | |  |  |
| C4 | DDX50 |  |  |  | |  |  |
| C4 | VTI1B |  |  |  | |  |  |
| C4 | NCOR2 |  |  |  | |  |  |
| C4 | ACSL5 |  |  |  | |  |  |
| C4 | PPL |  |  |  | |  |  |
| C4 | CHMP3 |  |  |  | |  |  |
| C4 | PSPC1 |  |  |  | |  |  |
| C4 | ARRDC1 |  |  |  | |  |  |
| C4 | PCNP |  |  |  | |  |  |
| C4 | CTNNA1 |  |  |  | |  |  |
| C4 | AKR1C3 |  |  |  | |  |  |
| C4 | TMEM123 |  |  |  | |  |  |
| C4 | WDR77 |  |  |  | |  |  |
| C4 | BCAS2 |  |  |  | |  |  |
| C4 | QPCT |  |  |  | |  |  |
| C4 | MGST1 |  |  |  | |  |  |
| C4 | BAZ2B |  |  |  | |  |  |
| C4 | FRMD8 |  |  |  | |  |  |
| C4 | PPIL4 |  |  |  | |  |  |
| C4 | COX14 |  |  |  | |  |  |
| C4 | STRAP |  |  |  | |  |  |
| C4 | GSTO2 |  |  |  | |  |  |
| C4 | GPX4 |  |  |  | |  |  |
| C4 | PPA2 |  |  |  | |  |  |
| C4 | HMGN2 |  |  |  | |  |  |
| C4 | PA2G4 |  |  |  | |  |  |
| C4 | BTG1 |  |  |  | |  |  |
| C4 | MSRA |  |  |  | |  |  |
| C4 | DDX17 |  |  |  | |  |  |
| C4 | ZFP36L1 |  |  |  | |  |  |
| C4 | THYN1 |  |  |  | |  |  |
| C4 | PAK6 |  |  |  | |  |  |
| C4 | KIAA2026 |  |  |  | |  |  |
| C4 | ATN1 |  |  |  | |  |  |
| C4 | SNX7 |  |  |  | |  |  |
| C4 | HNRNPF |  |  |  | |  |  |
| C4 | FBXO25 |  |  |  | |  |  |
| C4 | MRPL33 |  |  |  | |  |  |
| C4 | TUBG2 |  |  |  | |  |  |
| C4 | ATP6V1F |  |  |  | |  |  |
| C4 | VDAC3 |  |  |  | |  |  |
| C4 | ZCRB1 |  |  |  | |  |  |
| C4 | EPHX1 |  |  |  | |  |  |
| C4 | TRNP1 |  |  |  | |  |  |
| C4 | LMBR1L |  |  |  | |  |  |
| C4 | PTPN6 |  |  |  | |  |  |
| C4 | DYNLT1 |  |  |  | |  |  |
| C4 | LMO4 |  |  |  | |  |  |
| C4 | SMAD3 |  |  |  | |  |  |
| C4 | RAN |  |  |  | |  |  |
| C4 | MMADHC |  |  |  | |  |  |
| C4 | SLU7 |  |  |  | |  |  |
| C4 | TSKU |  |  |  | |  |  |
| C4 | SMARCA5 |  |  |  | |  |  |
| C4 | CREBL2 |  |  |  | |  |  |
| C4 | SSH3 |  |  |  | |  |  |
| C4 | KTN1 |  |  |  | |  |  |
| C4 | EIF3A |  |  |  | |  |  |
| C4 | ARL3 |  |  |  | |  |  |
| C4 | ATXN10 |  |  |  | |  |  |
| C4 | TSC22D3 |  |  |  | |  |  |
| C4 | SDC2 |  |  |  | |  |  |
| C4 | PLBD1 |  |  |  | |  |  |
| C4 | UXT |  |  |  | |  |  |
| C4 | CCDC12 |  |  |  | |  |  |
| C4 | CPQ |  |  |  | |  |  |
| C4 | KRT8 |  |  |  | |  |  |
| C4 | MED31 |  |  |  | |  |  |
| C4 | ACTN4 |  |  |  | |  |  |
| C4 | NDUFA13 |  |  |  | |  |  |
| C4 | BNIP2 |  |  |  | |  |  |
| C4 | NPC2 |  |  |  | |  |  |
| C4 | PCID2 |  |  |  | |  |  |
| C4 | ORMDL2 |  |  |  | |  |  |
| C4 | ITGB1BP1 |  |  |  | |  |  |
| C4 | MARCKSL1 |  |  |  | |  |  |
| C4 | PLXDC2 |  |  |  | |  |  |
| C4 | CALM1 |  |  |  | |  |  |
| C4 | SPSB2 |  |  |  | |  |  |
| C4 | NMT1 |  |  |  | |  |  |
| C4 | PPIL3 |  |  |  | |  |  |
| C4 | GAPDH |  |  |  | |  |  |
| C4 | ARF3 |  |  |  | |  |  |
| C4 | ATP6V1G1 |  |  |  | |  |  |
| C4 | ATP6V0E1 |  |  |  | |  |  |
| C4 | RBX1 |  |  |  | |  |  |
| C4 | ANP32A |  |  |  | |  |  |
| C4 | RBBP8 |  |  |  | |  |  |
| C4 | CCDC91 |  |  |  | |  |  |
| C4 | PLRG1 |  |  |  | |  |  |
| C4 | TNFRSF12A |  |  |  | |  |  |
| C4 | ESYT1 |  |  |  | |  |  |
| C4 | KBTBD3 |  |  |  | |  |  |
| C4 | CIB1 |  |  |  | |  |  |
| C4 | PHF1 |  |  |  | |  |  |
| C4 | YWHAE |  |  |  | |  |  |
| C4 | ADH5 |  |  |  | |  |  |
| C4 | G0S2 |  |  |  | |  |  |
| C4 | ARL6IP1 |  |  |  | |  |  |
| C4 | NMRK1 |  |  |  | |  |  |
| C4 | CSRNP1 |  |  |  | |  |  |
| C4 | PFN2 |  |  |  | |  |  |
| C4 | XRCC6 |  |  |  | |  |  |
| C4 | NET1 |  |  |  | |  |  |
| C4 | PRKAR1A |  |  |  | |  |  |
| C4 | GTF3A |  |  |  | |  |  |
| C4 | CASP1 |  |  |  | |  |  |
| C4 | RNF167 |  |  |  | |  |  |
| C4 | GNG5 |  |  |  | |  |  |
| C4 | CDC16 |  |  |  | |  |  |
| C4 | STOML2 |  |  |  | |  |  |
| C4 | CDKN1A |  |  |  | |  |  |
| C4 | SCP2 |  |  |  | |  |  |
| C4 | CAP1 |  |  |  | |  |  |
| C4 | CDC42BPA |  |  |  | |  |  |
| C4 | UBE2B |  |  |  | |  |  |
| C4 | RAB20 |  |  |  | |  |  |
| C4 | DSTN |  |  |  | |  |  |
| C4 | NDUFB3 |  |  |  | |  |  |
| C4 | CTBP2 |  |  |  | |  |  |
| C4 | HOXA10 |  |  |  | |  |  |
| C4 | CRYZ |  |  |  | |  |  |
| C4 | ARL14 |  |  |  | |  |  |
| C4 | COX7A2 |  |  |  | |  |  |
| C4 | AP1G2 |  |  |  | |  |  |
| C4 | TPI1 |  |  |  | |  |  |
| C4 | HIBADH |  |  |  | |  |  |
| C4 | COPZ1 |  |  |  | |  |  |
| C4 | SUPT20H |  |  |  | |  |  |
| C4 | PHLDA2 |  |  |  | |  |  |
| C4 | ANAPC13 |  |  |  | |  |  |
| C4 | MLPH |  |  |  | |  |  |
| C4 | PROC |  |  |  | |  |  |
| C4 | MMRN2 |  |  |  | |  |  |
| C4 | SERP1 |  |  |  | |  |  |
| C4 | CD164 |  |  |  | |  |  |
| C4 | COX6B1 |  |  |  | |  |  |
| C4 | RPP25L |  |  |  | |  |  |
| C4 | ACAA1 |  |  |  | |  |  |
| C4 | CCDC59 |  |  |  | |  |  |
| C4 | HNRNPH3 |  |  |  | |  |  |
| C4 | PPP2R2B |  |  |  | |  |  |
| C4 | KRT10 |  |  |  | |  |  |
| C4 | SIAH2 |  |  |  | |  |  |

Table S3: Signature genes of each subgroup for NTP analysis.

| **Table S4. Clinical data summary and statistical analysis of four subgroups in TCGA BLCA cohort** | | | | | |
| --- | --- | --- | --- | --- | --- |
|  | **C1** | **C2** | **C3** | **C4** | **p** |
| n | 119 | 74 | 40 | 56 |  |
| Age = ≤65 (%) | 36 (31.6) | 36 (31.6) | 12 (10.5) | 30 (26.3) | **0** |
| Gender = Male (%) | 95 (44.0) | 53 (24.5) | 25 (11.6) | 43 (19.9) | **0.0046** |
| Stage (%) |  |  |  |  | **0.1502** |
| Stage I | 0 | 0 | 0 | 1 (100.0) |  |
| Stage II | 38 (39.6) | 19 (19.8) | 15 (15.6) | 24 (25.0) |  |
| Stage III | 31 (33.0) | 30 (32.0) | 15 (15.9) | 18 (19.1) |  |
| Stage IV | 48 (50.0) | 25 (26.0) | 10 (10.4) | 13 (13.6) |  |
| Grade = High (%) | 115 (42.6) | 74 (27.4) | 37 (13.7) | 44 (16.3) | **0.4396** |
| T (%) |  |  |  |  | **0.0954** |
| T1 | 0 | 0 | 0 | 1(100.0) |  |
| T2 | 37 (39.8) | 19 (20.4) | 13 (14.0) | 24 (25.8) |  |
| T3 | 55 (40.7) | 38 (28.1) | 21 (15.6) | 21 (15.6) |  |
| T4 | 14 (36.8) | 12 (31.6) | 4 (10.5) | 8 (21.1) |  |
| N (%) |  |  |  |  | **0.4089** |
| N0 | 57 (34.3) | 42 (25.3) | 26 (15.7) | 41 (24.7) |  |
| N1 | 17 (48.6) | 9 (25.7) | 6 (17.1) | 3 (8.6) |  |
| N2 | 28 (53.8) | 12 (23.1) | 3 (5.8) | 9 (17.3) |  |
| N3 | 3 (60.0) | 2 (40.0) | 0 | 0 |  |
| M = M1 (%) | 5 (62.5) | 2 (25) | 1 (12.5) | 0 | **0.1092** |
| Growth Pattern = Papillary (%) | 40 (42.5) | 12 (12.8) | 14 (14.9) | 28 (29.8) | **0** |

Table S4: Clinical data summary and statistical analysis of four subtypes in TCGA cohort.

| **Table S5. Cell death signaling pathways** | | |
| --- | --- | --- |
| **Cell death types** | **Pathway** | **Source** |
| Apoptosis | REACTOME_APOPTOSIS | <http://www.gsea-msigdb.org/gsea/msigdb/human/search.jsp> |
| Apoptosis | REACTOME_INTRINSIC_PATHWAY_FOR_APOPTOSIS | <http://www.gsea-msigdb.org/gsea/msigdb/human/search.jsp> |
| Apoptosis | REACTOME_APOPTOSIS_INDUCED_DNA_FRAGMENTATION | <http://www.gsea-msigdb.org/gsea/msigdb/human/search.jsp> |
| Apoptosis | REACTOME_TP53_REGULATES_TRANSCRIPTION_OF_SEVERAL_ADDITIONAL_CELL_DEATH_GENES_WHOSE_SPECIFIC_ROLES_IN_P53_DEPENDENT_APOPTOSIS_REMAIN_UNCERTAIN | <http://www.gsea-msigdb.org/gsea/msigdb/human/search.jsp> |
| Apoptosis | REACTOME_DEFECTIVE_INTRINSIC_PATHWAY_FOR_APOPTOSIS | <http://www.gsea-msigdb.org/gsea/msigdb/human/search.jsp> |
| Apoptosis | KEGG_APOPTOSIS | <http://www.gsea-msigdb.org/gsea/msigdb/human/search.jsp> |
| Apoptosis | HALLMARK_APOPTOSIS | <http://www.gsea-msigdb.org/gsea/msigdb/human/search.jsp> |
| Apoptosis | GOBP_MEIOTIC_SPINDLE_ASSEMBLY | <http://www.gsea-msigdb.org/gsea/msigdb/human/search.jsp> |
| Apoptosis | GOBP_CARDIAC_MUSCLE_MYOBLAST_PROLIFERATION | <http://www.gsea-msigdb.org/gsea/msigdb/human/search.jsp> |
| Apoptosis | GOBP_REGULATION_OF_ACTIN_FILAMENT_ORGANIZATION | <http://www.gsea-msigdb.org/gsea/msigdb/human/search.jsp> |
| Apoptosis | GOBP_HIPPOCAMPAL_NEURON_APOPTOTIC_PROCESS | <http://www.gsea-msigdb.org/gsea/msigdb/human/search.jsp> |
| Apoptosis | GOBP_NEGATIVE_REGULATION_OF_STEM_CELL_PROLIFERATION | <http://www.gsea-msigdb.org/gsea/msigdb/human/search.jsp> |
| Autophagy | REACTOME_CHAPERONE_MEDIATED_AUTOPHAGY | <http://www.gsea-msigdb.org/gsea/msigdb/human/search.jsp> |
| Autophagy | REACTOME_LATE_ENDOSOMAL_MICROAUTOPHAGY | <http://www.gsea-msigdb.org/gsea/msigdb/human/search.jsp> |
| Autophagy | KEGG_REGULATION_OF_AUTOPHAGY | <http://www.gsea-msigdb.org/gsea/msigdb/human/search.jsp> |
| Autophagy | BIOCARTA_CARDIACEGF_PATHWAY | <http://www.gsea-msigdb.org/gsea/msigdb/human/search.jsp> |
| Autophagy | GOBP_RESPONSE_TO_MECHANICAL_STIMULUS | <http://www.gsea-msigdb.org/gsea/msigdb/human/search.jsp> |
| Autophagy | GOBP_RESPONSE_TO_VIRUS | <http://www.gsea-msigdb.org/gsea/msigdb/human/search.jsp> |
| Autophagy | GOBP_RESPONSE_TO_BACTERIUM | <http://www.gsea-msigdb.org/gsea/msigdb/human/search.jsp> |
| Autophagy | GOBP_BILE_ACID_AND_BILE_SALT_TRANSPORT | <http://www.gsea-msigdb.org/gsea/msigdb/human/search.jsp> |
| Autophagy | GOBP_OXALOACETATE_TRANSPORT | <http://www.gsea-msigdb.org/gsea/msigdb/human/search.jsp> |
| Autophagy | GOBP_PROSTAGLANDIN_TRANSPORT | <http://www.gsea-msigdb.org/gsea/msigdb/human/search.jsp> |
| Autophagy | GOBP_SIALIC_ACID_TRANSPORT | <http://www.gsea-msigdb.org/gsea/msigdb/human/search.jsp> |
| Autophagy | GOBP_REGULATION_OF_CEREBELLAR_GRANULE_CELL_PRECURSOR_PROLIFERATION | <http://www.gsea-msigdb.org/gsea/msigdb/human/search.jsp> |
| Autophagy | GOBP_TOLL_LIKE_RECEPTOR_4_SIGNALING_PATHWAY | <http://www.gsea-msigdb.org/gsea/msigdb/human/search.jsp> |
| Autophagy | GOBP_NCRNA_3_END_PROCESSING | <http://www.gsea-msigdb.org/gsea/msigdb/human/search.jsp> |
| Autophagy | GOBP_REGULATION_OF_ORGANELLE_TRANSPORT_ALONG_MICROTUBULE | <http://www.gsea-msigdb.org/gsea/msigdb/human/search.jsp> |
| Autophagy | GOBP_POSITIVE_REGULATION_OF_PROTEIN_K63_LINKED_UBIQUITINATION | <http://www.gsea-msigdb.org/gsea/msigdb/human/search.jsp> |
| Autophagy | GOBP_NEGATIVE_REGULATION_OF_LEUKOCYTE_CELL_CELL_ADHESION | <http://www.gsea-msigdb.org/gsea/msigdb/human/search.jsp> |
| Autophagy | GOBP_REGULATION_OF_DENDRITE_EXTENSION | <http://www.gsea-msigdb.org/gsea/msigdb/human/search.jsp> |
| Autophagy | GOBP_POSITIVE_REGULATION_OF_VASCULATURE_DEVELOPMENT | <http://www.gsea-msigdb.org/gsea/msigdb/human/search.jsp> |
| Necrosis | REACTOME_RIPK1_MEDIATED_REGULATED_NECROSIS | <http://www.gsea-msigdb.org/gsea/msigdb/human/search.jsp> |
| Necrosis | REACTOME_REGULATED_NECROSIS | <http://www.gsea-msigdb.org/gsea/msigdb/human/search.jsp> |
| Necrosis | REACTOME_DEFECTIVE_RIPK1_MEDIATED_REGULATED_NECROSIS | <http://www.gsea-msigdb.org/gsea/msigdb/human/search.jsp> |
| Necrosis | GOBP_POSITIVE_REGULATION_OF_PHOSPHOLIPASE_ACTIVITY | <http://www.gsea-msigdb.org/gsea/msigdb/human/search.jsp> |
| Necrosis | GOBP_NEGATIVE_REGULATION_OF_PHOSPHOLIPASE_ACTIVITY | <http://www.gsea-msigdb.org/gsea/msigdb/human/search.jsp> |
| Necrosis | GOBP_POSITIVE_REGULATION_OF_CREB_TRANSCRIPTION_FACTOR_ACTIVITY | <http://www.gsea-msigdb.org/gsea/msigdb/human/search.jsp> |
| Necrosis | GOBP_POSITIVE_REGULATION_OF_MAINTENANCE_OF_SISTER_CHROMATID_COHESION | <http://www.gsea-msigdb.org/gsea/msigdb/human/search.jsp> |
| Necrosis | GOBP_CELLULAR_RESPONSE_TO_ETHANOL | <http://www.gsea-msigdb.org/gsea/msigdb/human/search.jsp> |
| Necrosis | GOBP_PROTON_TRANSMEMBRANE_TRANSPORT | <http://www.gsea-msigdb.org/gsea/msigdb/human/search.jsp> |
| Necrosis | GOBP_POSITIVE_REGULATION_OF_PROTEIN_DEUBIQUITINATION | <http://www.gsea-msigdb.org/gsea/msigdb/human/search.jsp> |
| lysosome | BIOCARTA_AMAN_PATHWAY | <http://www.gsea-msigdb.org/gsea/msigdb/human/search.jsp> |
| lysosome | GOBP_DNA_CATABOLIC_PROCESS_ENDONUCLEOLYTIC | <http://www.gsea-msigdb.org/gsea/msigdb/human/search.jsp> |
| lysosome | GOBP_NEGATIVE_REGULATION_OF_RESPONSE_TO_EXTERNAL_STIMULUS | <http://www.gsea-msigdb.org/gsea/msigdb/human/search.jsp> |
| lysosome | GOBP_CARDIAC_MUSCLE_CELL_FATE_COMMITMENT | <http://www.gsea-msigdb.org/gsea/msigdb/human/search.jsp> |
| lysosome | GOBP_NEGATIVE_REGULATION_OF_CELL_GROWTH_INVOLVED_IN_CARDIAC_MUSCLE_CELL_DEVELOPMENT | <http://www.gsea-msigdb.org/gsea/msigdb/human/search.jsp> |
| lysosome | GOBP_T_CELL_MIGRATION | <http://www.gsea-msigdb.org/gsea/msigdb/human/search.jsp> |
| lysosome | GOBP_REGULATION_OF_INCLUSION_BODY_ASSEMBLY | <http://www.gsea-msigdb.org/gsea/msigdb/human/search.jsp> |
| lysosome | GOBP_PROTEIN_MATURATION_BY_IRON_SULFUR_CLUSTER_TRANSFER | <http://www.gsea-msigdb.org/gsea/msigdb/human/search.jsp> |
| lysosome | GOBP_REGULATION_OF_NADP_METABOLIC_PROCESS | <http://www.gsea-msigdb.org/gsea/msigdb/human/search.jsp> |
| lysosome | GOBP_POSITIVE_REGULATION_OF_VASCULAR_ASSOCIATED_SMOOTH_MUSCLE_CELL_MIGRATION | <http://www.gsea-msigdb.org/gsea/msigdb/human/search.jsp> |
| lysosome | REACTOME_LYSOSOME_VESICLE_BIOGENESIS | <http://www.gsea-msigdb.org/gsea/msigdb/human/search.jsp> |
| pyroptosis | REACTOME_PYROPTOSIS | <http://www.gsea-msigdb.org/gsea/msigdb/human/search.jsp> |
| pyroptosis | GOBP_HISTONE_H3_K9_MODIFICATION | <http://www.gsea-msigdb.org/gsea/msigdb/human/search.jsp> |
| Necroptosis | GOBP_NECROPTOTIC_SIGNALING_PATHWAY | <http://www.gsea-msigdb.org/gsea/msigdb/human/search.jsp> |

Table S5: Cell death signaling pathways.
